# Time to diagnosis and treatment of obstructive sleep apnoea using mandibular jaw movement monitoring versus polysomnography: an open-label, multicentre, randomised, controlled trial

**DOI:** 10.1016/j.lanepe.2026.101637

**Published:** 2026-03-17

**Authors:** Jean-Louis Pépin, Renaud Tamisier, Marc Manceau, Katleen Denoncin, Arnaud Prigent, Maxime Patout, Hervé Pégliasco, Frédéric Gagnadoux, Jean-Benoît Martinot, Matthieu Roustit, SUNSAS Study Investigators, Sarah Alexandre, Sébastien Baillieul, Lucie Barateau, Clara Bianquis, Yvan Bellanger, Hélène Benzaquen, Laurent Boyer, Nicolas Carpentier, Elena Charbonnier, Ari Chaouat, Rita Clin, Julien Coelho, Ala Covali, Yves Dauvilliers, Claire Denis, Marie Destors, Marie Pia d’Ortho, Antoine Dumazet, Justine Frija, Sacha Gaillard, Thibaut Gentina, Maëlle Guellerin, Kelly Guichard, Laurence Hertert-Gandolfo, Benjamin Huret, Antoine Jaffiol, François Jounieaux, Philippe Lang, Claire Launois, Damien Léger, Smaranda Leu-Semenescu, Rim Leymarie, Cécile Londner, Quentin Lorber, Marie-Noëlle Lothe-Cartier, Régis Luraine, Guillaume Marchand, Nicole Meslier, Jean-Arthur Micoulaud-Franchi, Pierre-Jean Monteyrol, Pauline Mulette, Marina Ogier, Cécile Olivier, Caroline Pagniez, Albert Pajon, Yasmina Pascaud-Mansour, Laure Peter-Derex, Pierre Philip, Carole Planès, Sandrine Pontier-Marchandise, Vincent Puel, Nathalie Raymond, Bruno Ribeiro-Baptista, Rodrigue Ribereau, François Ricordeau, Cécile Ropars, Marc Sapène, Kamila Sedkaoui, Emeric Stauffer, Jonathan Taieb, Marion Tailland, Annie Tangtakoun, Pierre Tankere, Wojciech Trzepizur, Camille Valery, Olivier Varnet, Anne Wittenberg

**Affiliations:** a Université Grenoble Alpes, Laboratoire HP2, INSERM U1300, Grenoble, France; b Service Pneumologie Physiologie, CHU Grenoble Alpes, Grenoble, France; c Sunrise, Namur, Belgium; d Polyclinique Saint-Laurent, Rennes, France; e La Pitié Salpêtrière University Hospital, Pulmonology and Sleep Department, FHU UMANHYS, Sorbonne University, Paris, France; f Hôpital Européen, Marseille, France; g Department of Respiratory and Sleep Medicine, Angers University Hospital, Angers, France; h Sleep Laboratory, CHU Université catholique de Louvain (UCL), Namur Site Sainte-Elisabeth, Namur, Belgium; i Institute of Experimental and Clinical Research, UCL Bruxelles Woluwe, Brussels, Belgium; j Université Grenoble Alpes, INSERM CIC1406, CHU Grenoble Alpes, Grenoble, France

**Keywords:** Obstructive sleep apnoea, Diagnosis, Mandibular jaw movements, Polysomnography, Home sleep test, Sleepiness, Quality of life, Work productivity

## Abstract

**Background:**

Obstructive sleep apnoea (OSA) is often underdiagnosed, highlighting the need for scalable diagnostic alternatives. The SUNSAS study compared a new device for at-home diagnosis of OSA (artificial intelligence [AI]-supported analysis of mandibular jaw movements [MJM]) with polysomnography (PSG) for time to diagnosis and treatment, and patient-reported outcomes.

**Methods:**

This prospective, multicentre, randomised, controlled, open-label study was conducted in France (October 2021–October 2024). Adults aged 18–80 years with suspected OSA were randomised (1:1) to undergo diagnostic testing using MJM monitoring (Sunrise) or PSG. Primary endpoints were assessed using hierarchical testing: 1. daytime sleepiness (Epworth Sleepiness Scale [ESS] score) at 3 months post-diagnosis and time to diagnosis; 2. time to treatment; and 3. daytime sleepiness at 3 months post-randomisation. Secondary endpoints included quality of life (Short Form-36, Quebec Sleep Questionnaire), work productivity (Work Productivity and Activity Impairment questionnaire), and positive airway pressure therapy adherence at 3 months after treatment initiation.

**Findings:**

Of 849 participants randomised (58·7% male, median age 50 years, body mass index 28·0 kg/m^2^, apnoea-hypopnoea index 15·2/h), 774 received a diagnosis: 133 no OSA, 239 mild OSA, 220 moderate OSA, and 182 severe OSA. Median time to diagnosis (15 vs. 106 days) and to treatment initiation (50 vs. 124 days) were significantly shorter with MJM analysis versus PSG (both p < 0·01). MJM-based diagnosis was noninferior to PSG in reducing ESS at 3 months after diagnosis (−2·26 vs. −2·29; 95% confidence interval [CI] for difference −0·85, 0·79; p = 0·01), and superior at 3 months post-randomisation (between-group difference: −1·51 (95% CI −2·17, −0·85); p < 0·01). Secondary endpoints also favoured the MJM group.

**Interpretation:**

OSA diagnosis based on MJM monitoring with AI-supported analysis is noninferior to PSG in reducing daytime sleepiness at 3 months after diagnosis, while significantly accelerating time to diagnosis and treatment initiation, resulting in earlier improvement in daytime sleepiness.

**Funding:**

Sunrise, with support from the French Ministry of Health through the *Forfait Innovation* programme.

**Research in context**

**Evidence before this study**

Obstructive sleep apnoea (OSA) is a highly prevalent disorder associated with cardiometabolic comorbidities, alterations in quality of life, and early mortality. Polysomnography (PSG) remains the gold standard for OSA diagnosis but is resource-intensive, costly, and limited in availability, creating major bottlenecks in access to care. Monitoring of mandibular jaw movements (MJM) has recently emerged as an alternative for detecting OSA. We systematically searched PubMed and Embase between Jan 1, 2010, and Oct 6, 2025, with no language restrictions, using the terms (“sleep apnoea” OR “sleep apnea”) AND “diagnosis” AND “home sleep testing” AND (“mandibular movement” OR “mandibular jaw movement^∗^”). Our search found that validation of new OSA diagnostic tools has primarily focused on replicating indices provided by the ‘gold-standard’ PSG. In that context, robust evidence shows that MJM monitoring with artificial intelligence-supported analysis achieves diagnostic performance comparable to PSG in both laboratory and home settings. However, diagnostic performance alone does not establish clinical utility. It remains crucial to determine whether such approaches speed up and expand access to treatment, and improve clinical outcomes. In addition, for a new diagnostic tool to have real-world impact, it must be easy to use and scalable across populations.

**Added value of this study**

This randomised controlled trial of 849 patients across 18 centres in France is the largest to date comparing an at-home, end-to-end diagnostic solution for OSA based on artificial intelligence-supported MJM analysis with standard PSG (whether in-laboratory or at-home). Shorter times to diagnosis and treatment, and greater improvements in daytime sleepiness were observed at 3 months post-randomisation in the MJM group. Exploratory findings also suggested a positive impact on health-related quality of life and work productivity, supporting the clinical value of the MJM-based approach.

**Implications of all the available evidence**

This study provides robust evidence that at-home monitoring of MJM with artificial intelligence-supported analysis is a pragmatic alternative to PSG for OSA diagnosis, reducing diagnostic delays and improving timely access to therapy. Timely and accessible OSA diagnosis addresses an important unmet need in the field and might contribute to better health outcomes.

## Introduction

Obstructive sleep apnoea (OSA) is a highly prevalent condition^1^ that is characterised by repeated airway obstructions during sleep that lead to intermittent hypoxia and sleep fragmentation.^2^ Daytime sleepiness is a common OSA symptom but the disease has more wide-ranging and significant impacts. These include increased risk of cardiovascular and metabolic disorders, cognitive decline, and cardiovascular and all-cause death.^3^, ^4^, ^5^, ^6^ OSA is therefore a significant public health issue.^7^ Effective primary therapies for OSA are available (including positive airway pressure [PAP]) and these reduce individual and societal burden,^8^^,^^9^ healthcare resource utilisation and costs.^8^, ^9^, ^10^, ^11^

Early detection and treatment of OSA is essential to mitigate the negative health impacts of the condition. However, access to diagnosis is limited and a significant proportion of individuals with OSA remain undiagnosed (and therefore untreated).^12^ In-laboratory polysomnography (PSG) is the gold standard for diagnosing OSA.^13^ However, this requires an overnight stay in a specialised centre with trained staff. The cost and complexity of PSG, along with the rising global prevalence of OSA,^7^ have created significant bottlenecks in access to care. Many regions, including France, have long waiting lists and the burden of PSG could discourage individuals from seeking a formal OSA diagnosis.^14^ Therefore, alternative, easily scalable care pathways that improve access and reduce diagnostic delays are key priorities in OSA management.^15^

Over the past decade, the main development in OSA diagnostic testing has been the introduction of at-home PSG and home sleep apnoea testing, consisting in portable monitoring systems with a limited number of sensors which are primarily used in patients with a high pre-test probability of OSA.^16^

A new device has been designed to diagnose OSA using monitoring of mandibular jaw movements (MJM) with artificial intelligence (AI)-supported analysis. This device uses a single-point sensor placed on the chin overnight to record MJM, which reflect respiratory effort and airway dynamics during sleep, enabling accurate measurement of total sleep time, sleep staging, and differentiation between central and obstructive events.^17^ Validation studies have shown diagnostic performance comparable to manually scored PSG, both in sleep laboratories and in-home settings.^18^, ^19^, ^20^

The SUNSAS study was designed to evaluate whether this single-point, end-to-end home sleep apnoea testing solution using AI-supported analysis of mandibular jaw movements could improve the diagnostic and treatment pathway for OSA. This novel approach was compared with current reference practice (at-home or in-laboratory PSG) in terms of time to OSA diagnosis and treatment, and whether any differences would translate into earlier improvements in daytime sleepiness and other patient-reported outcomes.

## Methods

### Study design

The SUNSAS trial was a prospective, multicentre (n = 18), randomised, controlled, parallel-group, open-label study conducted in France from 28 October 2021 to 7 October 2024. The study protocol was approved by the French National Authority for Health (HAS) under the framework of the *Forfait Innovation* programme, a French national initiative supporting the evaluation of innovative medical technologies with potential to improve care. The study was designed by the academic team at the University of Grenoble Alpes and then discussed and approved by the HAS and study sponsor (Sunrise). The trial is registered with ClinicalTrials.gov, number NCT05057975. The list of principal investigators is provided in the Supplementary Appendix. The study protocol version 1.7 dated June 13, 2022, and the statistical analysis plan version 1.0 dated November 18, 2024, are provided in the Appendix.

This trial was not monitored by a Data and Safety Monitoring Board (DSMB) as such oversight is not routinely recommended for pragmatic post-market trials involving minimal participant risk and no planned interim analyses.

### Participants

Adults aged 18–80 years referred for suspicion of OSA were eligible. Key exclusion criteria included any diagnostic procedure for OSA within the previous five years, any initiation of treatment for OSA, severe chronic obstructive or restrictive pulmonary disease (with or without oxygen therapy), unstable cardiovascular disease and severe heart failure. Patients prioritised for OSA diagnosis (e.g., truck drivers, night workers) were also excluded. The full list of eligibility criteria is available in the Supplementary Appendix. Biological sex at birth (male or female) was self-reported. Race and ethnicity data were not collected.

### Randomisation and masking

SUNSAS is an open-label trial. After a screening visit, eligible participants were randomly assigned (1:1) to undergo OSA diagnostic testing using MJM monitoring (Sunrise, Namur, Belgium) (Supplementary Figure S1) for up to three nights at home or a single standard overnight PSG (in-laboratory or in–home), with randomisation stratified by baseline ESS score (≤12 or >12). Randomisation was centralised and performed electronically using random block sizes, with the allocation sequence implemented in the software by an independent academic statistician who was not involved in the final analysis.

### Procedures

After providing informed consent, eligible participants were randomised to one of two specific diagnosis pathways, i.e., MJM monitoring or overnight PSG. The MJM monitoring device required a minimum of 4 h of use for a valid recording. MJM recordings were analysed using AI, as previously described.^18^ The MJM analysed data were integrated into a detailed report made available to physicians via an electronic file. The diagnosis was made based on the first valid recording in the MJM group. PSG studies were manually scored according to American Academy of Sleep Medicine criteria.^21^ Scoring was performed either by physicians or sleep technicians under physician supervision, consistent with routine clinical practice at each study centre.

After the diagnostic procedure, participants attended a diagnostic consultation to review the results of their sleep study and discuss any required treatment. Follow-up visits were scheduled at 3 months after randomisation and 3 months after the diagnostic consultation; an additional follow-up visit was conducted 3 months after therapy initiation for patients prescribed treatment (Supplementary Figure S2). Each centre selected an appropriate treatment for patients diagnosed with OSA (PAP or an oral appliance) based on their clinical experience and applicable French guidelines. Mild OSA was managed using lifestyle modifications in the first instance. A participant was considered to have completed the study upon attending their final protocol-defined visit. Right-censoring was applied at the date of last contact for participants who did not experience the event because of early study exit (e.g., withdrawal, loss to follow-up, or completion of the maximum 18-month study period without event occurrence).

### Outcomes

Primary endpoints were tested in the following prespecified hierarchical order: 1. daytime sleepiness at 3 months after the diagnostic consultation (noninferiority) and time to diagnostic consultation (superiority); 2. time to treatment initiation (superiority); and 3. daytime sleepiness at 3 months after randomisation (superiority). Secondary (exploratory) endpoints included changes in quality of life and work productivity from baseline to 3 months after randomisation, and PAP adherence at 3 months after treatment initiation. Additional secondary objectives will be reported separately. These include an economic evaluation of the impact of the MJM monitoring device within the French care pathway, night-to-night variability of the apnoea-hypopnoea index (AHI) assessed using up to three nights of MJM recordings, and an analysis of MJM-based device performance relative to PSG.

Daytime sleepiness was evaluated using the Epworth Sleepiness Scale (ESS). Quality of life was determined using the Short Form-36 (SF-36) and the Quebec Sleep Questionnaire (QSQ). Work productivity was measured using the Work Productivity and Activity Impairment (WPAI) questionnaire. Full details of these measures are provided in the Supplementary Appendix.

Investigators were required to report all serious adverse events and adverse device effects during the course of the study.

### Statistical analysis

The sample size was calculated to assess the noninferiority of OSA diagnosis based on MJM monitoring over PSG for the endpoint of daytime sleepiness at 3 months post-diagnosis. Using a minimal clinically important difference (MCID) in the ESS score of 2·5, noninferiority was defined as a 1-point between-group difference in the change in ESS score. Assuming a standard deviation of 4·6 points,^22^^,^^23^ 381 patients per group would provide 85% power at a one-sided alpha level of 0·025. Target enrolment was set at 848 participants allowing for a 10% dropout rate. Given the absence of any preliminary data regarding the expected difference in time to diagnosis, the sample size calculation was not based on this endpoint. However, the chosen sample size was estimated to be sufficient to detect an effect size greater than 0·25 (Cohen's d) for time to diagnosis, using a one-sided test at the 0·025 significance level.

Continuous variables are reported as median (interquartile range) and qualitative variables are reported as number of participants (percentage). Between-group differences in daytime sleepiness at 3 months post-diagnosis and PAP adherence at 3 months after treatment initiation were evaluated using noninferiority analyses. All other endpoints were evaluated using superiority analyses.

Superiority analyses were conducted using the modified intention-to-treat (mITT) population as the main analysis and the per-protocol (PP) population as a secondary analysis, whereas noninferiority was primarily based on the PP population and secondarily on the mITT population. The mITT population included all randomised participants with available data. The PP population included all randomised patients with available data and no major protocol deviations. Replacement of missing data was performed for ESS scores as part of sensitivity analyses for the primary endpoints (not for survival analyses). Missing data were imputed following Rubin's multiple imputation framework, by using the R ‘mice’ package. A bootstrap-based multiple imputation approach was used under a linear model including demographic and clinical covariates available for most participants (e.g., age, sex, cardiovascular history, and sleep-related measures) and the randomised study arm (i.e., MJM or PSG).^24^

To control the overall type I error rate for all primary outcomes, we used a prespecified hierarchical testing approach (Supplementary Figure S3 for full details). Each subsequent level was tested only if the null hypothesis at the preceding level was rejected. A one-sided significance level of 0·025 was applied at each step.

Noninferiority of diagnosis based on MJM monitoring compared with PSG for the change in ESS score from baseline to 3 months post-diagnosis was assessed using a one-sided t-test. Superiority analysis for the ESS score at 3 months post-randomisation was tested using analysis of covariance (ANCOVA), with baseline ESS score included as a covariate. Time-to-event endpoints were analysed using Kaplan–Meier estimators. Between-group comparisons were performed using Cox proportional hazards models. Primary outcome analyses were checked by an independent statistical team at the University of Grenoble Alpes.

Statistical analyses were performed using R software (version 4.3) with standard packages, including tidyverse. All code required to reproduce the analyses is included in the statistical analysis report.

### Ethics approval

The study was approved by the Ethics Committee in France (Comité de Protection des Personnes Ile de France IV, CPP IDF IV: 2021-A01827-34) on September 2, 2021. The study was conducted in accordance with Declaration of Helsinki principles, and Good Clinical Practice guidelines of the International Council for Harmonisation (ICH-GCP). All participants provided written informed consent.

### Role of the funding source

The funder of the study had no role in the study design, data collection, data analysis, and interpretation. The funder collaborated with the academic authors on the development of each draft of the manuscript, with editorial assistance funded by the University of Grenoble Alpes. The funder provided MJM monitoring sensors, with support from the French Ministry of Health through the *Forfait Innovation* programme.

## Results

**Fig. 1:**
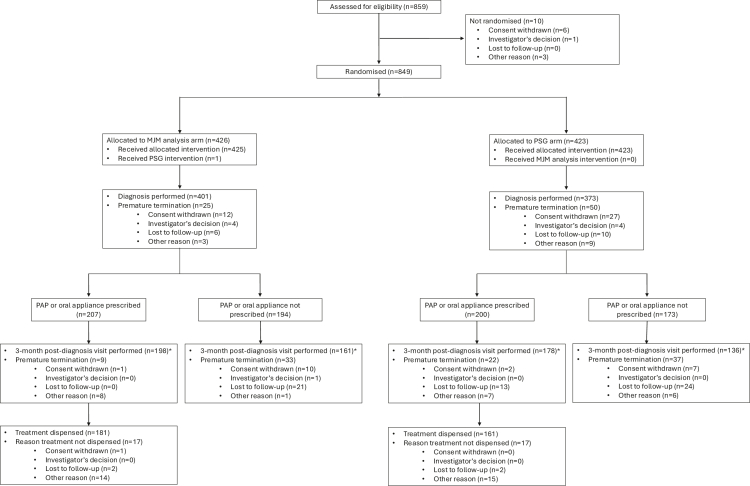
CONSORT flow diagram. MJM = mandibular jaw movements; OSA = obstructive sleep apnoea; PSG = polysomnography. The modified intention-to-treat (mITT) population for the noninferiority evaluation of the Epworth Sleepiness Scale score at 3 months after diagnosis included 335 patients in the MJM group, and 292 patients in the PSG group. The per-protocol (PP) population (main analysis) included 310 patients in the MJM group and 263 patients in the PSG group.

A total of 849 participants were randomised (median age 50 years, 58·7% male, median body mass index 28·0 kg/m^2^, median AHI 15·2/h) (Table 1). Of these, 774 (91·2%) received a diagnosis of OSA or no OSA, and 627 (73·9%) completed the 3-month post-diagnosis visit (Fig. 1). Overall, 133 participants (17·2%) were found not to have OSA, 239 (30·9%) had mild OSA (AHI 5 to <15/h), 220 (28·4%) had moderate OSA (AHI 15 to <30/h), and 182 (23·5%) had severe OSA (AHI ≥30/h), with no difference in OSA severity distribution between the MJM and PSG groups. In the MJM analysis group, 36% of patients were treated with PAP and 15% with an oral appliance, and in the PSG arm, 40% were treated with PAP and 13% with an oral appliance. Details regarding the geographic distribution and representativeness of trial participants are provided in Supplementary Table S1.

**Table 1:** Demographic and clinical characteristics of study participants at enrolment.

Characteristic	MJM (n = 426)	PSG (n = 423)	Overall (n = 849)
Age, years	50·0 (39·2; 60·0)	50·0 (39·0; 59·0)	50·0 (39·0; 60·0)
Female sex, n (%)	166 (39·0)	185 (43·7)	351 (41·3)
Body weight, kg	85·0 (74·0; 98·0)	83·0 (70·5; 98·0)	84·0 (72·0; 98·0)
Body mass index, kg/m^2^	28·1 (24·9; 33·0)	27·5 (24·5; 32·5)	28·0 (24·7; 32·8)
Waist circumference, cm	106·0 (100·0; 115·0)	105·0 (98·0; 115·0)	106·0 (99·0; 115·0)
Neck circumference, cm	39·0 (37·0; 43·0)	39·0 (35·0; 42·0)	39·0 (36·0; 42·2)
Current smoker, n (%)	76 (17·8)	80 (18·9)	156 (18·4)
AHI on first night of recording, events/h	12·8 (6·7; 24·5)	17·0 (6·9; 31·0)	15·2 (6·8; 27·4)
ESS score	11·0 (7·0; 15·0)	11·0 (7·0; 15·0)	11·0 (7·0; 15·0)
Systolic blood pressure, mmHg	130·0 (120·0; 140·0)	126·0 (120·0; 138·0)	128·5 (120·0; 139·2)
Diastolic blood pressure, mmHg	80·0 (70·0; 88·5)	80·0 (72·0; 87·0)	80·0 (71·0; 88·0)
Hypertension, n (%)	87 (20·4)	89 (21·0)	176 (20·7)
Type 2 diabetes, n (%)	33 (7·7)	19 (4·5)	52 (6·1)
Depression, n (%)	35 (8·2)	27 (6·4)	62 (7·3)
COPD, n (%)	4 (0·9)	5 (1·1)	9 (1·0)
Stroke/transient ischaemic attack, n (%)	19 (4·5)	21 (5·0)	40 (4·7)
Cardiac failure, n (%)	1 (0·2)	3 (0·7)	4 (0·5)
Coronary heart disease, n (%)	9 (2·1)	14 (3·3)	23 (2·7)
Universal health coverage, n (%)	50 (12·3)	52 (12·6)	102 (12·4)
Use of hypnotic medications, n (%)	17 (4·0)	16 (3·8)	33 (3·9)

Values are median (interquartile range) or number of patients (%); percentages may not total 100 because of rounding.

Abbreviations: AHI = apnoea-hypopnoea index; COPD = chronic obstructive pulmonary disease; ESS = Epworth Sleepiness Scale; MJM = mandibular jaw movements; OSA = obstructive sleep apnoea; PSG = polysomnography.

**Fig. 2:**
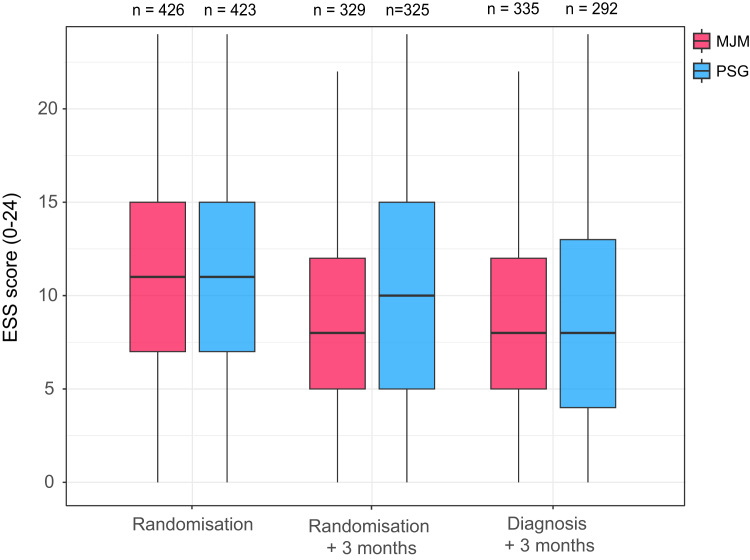
Boxplots of daytime sleepiness (Epworth Sleepiness Scale [ESS] score) at randomisation, 3 months post-randomisation, and 3 months post-diagnosis in the mandibular jaw movement (MJM) monitoring and polysomnography (PSG) groups. Numbers along the top of each graph indicate the number of patients that contributed data in each group at each time point.

All primary endpoints were met, and each step of the prespecified hierarchical testing procedure was successfully completed. OSA diagnosis with MJM monitoring was noninferior to PSG in reducing daytime sleepiness (ESS score) at 3 months post-diagnosis (−2·26 vs. −2·29, respectively; between-group difference in the PP population: −0·03 [95% confidence interval [CI]: −0·85 to 0·79]; p = 0·01) (Fig. 2). The same analysis performed in the mITT population yielded similar results (ESS score −2·28 vs. −2·40, respectively; between-group difference: −0·12 [95% CI: −0·89 to 0·66]; p = 0·013). The sensitivity analysis using imputed data also confirmed the noninferiority of MJM compared with PSG for ESS reduction at 3 months after diagnosis.

**Fig. 3:**
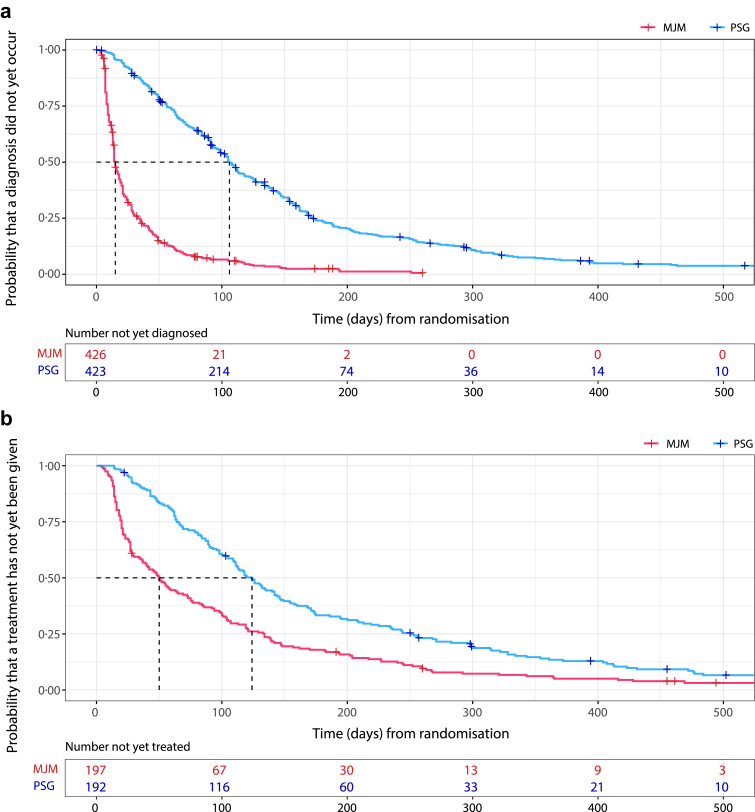
Kaplan–Meier curves showing time from randomisation to diagnosis (upper panel) and from randomisation to treatment initiation (lower panel) in the mandibular jaw movement (MJM) monitoring and polysomnography (PSG) groups.

Median time to diagnosis (15 vs. 106 days; p < 0·01) (Fig. 2) and median time to treatment initiation (50 vs. 124 days; p < 0·01) were significantly shorter using the new diagnostic test compared with PSG (Fig. 3). The time between diagnostic testing (i.e., MJM recording or PSG) and diagnosis consultation did not differ significantly between the MJM and PSG groups (Supplementary Figure S4). There was a significantly greater reduction in the ESS score at 3 months post-randomisation in the MJM monitoring versus PSG group (between-group difference: −1·51 [95% CI: −2·17, −0·85]; P < 0·01) (Fig. 2).

Participants with OSA diagnosis based on MJM monitoring showed significantly greater improvements in multiple SF-36 domains (vitality, social functioning, bodily pain, and general health perceptions) and QSQ domains (daytime sleepiness, diurnal symptoms, and nocturnal symptoms) compared with those diagnosed using PSG (Table 2). Presenteeism and activity impairment were also lower in the MJM group (Table 2).

**Table 2:** Patient-reported outcomes: changes from baseline to 3 months post-randomisation in quality of life and work productivity.

Variable	Baseline		Change from baseline		Between-group difference
	MJM	PSG	MJM	PSG	
**QSQ domain scores**^a^	N = 388	N = 382	N = 244	N = 260	
Daytime sleepiness	5·2 (4·0; 6·2)	5·3 (4·0; 6·2)	0·64 (0·48, 0·80)^b^	0·19 (0·06, 0·32)	0·41 (0·23, 0·59)
Diurnal symptoms	4·4 (3·2; 5·8)	4·5 (3·0; 6·0)	0·57 (0·41, 0·73)	0·26 (0·13, 0·38)	0·28 (0·09, 0·47)
Nocturnal symptoms	4·6 (3·6; 5·6)	4·9 (3·9; 5·7)	0·64 (0·49, 0·79)	0·29 (0·18, 0·41)	0·27 (0·10, 0·44)
Emotions	5·2 (4·2; 5·8)	5·2 (4·2; 6·0)	0·33 (0·20, 0·45)	0·16 (0·04, 0·27)	0·14 (−0·01, 0·29)
Social interactions	5·2 (4·4; 6·2)	5·5 (4·3; 6·2)	0·48 (0·33, 0·63)	0·29 (0·16, 0·41)	0·16 (−0·01, 0·33)
**SF–36 domain scores**^a^	N = 394	N = 387	N = 263	N = 274	
Physical functioning	90·0 (75·0; 95·0)	90·0 (75·0; 95·0)	0·00 (−1·58, 1·58)	−1·50 (−3·38, 0·39)	1·27 (−1·11, 3·65)
Role physical	75·0 (25·0; 100·0)^c^	75·0 (25·0; 100·0)	6·13 (1·91, 10·35)^d^	0·18 (−4·29, 4·66)	5·22 (−0·06, 10·51)
Role emotional	66·7 (33·3; 100·0)	66·7 (33·3; 100·0)	9·67 (4·78, 14·56)^c^	1·59 (−3·05, 6·22)^c^	5·59 (−0·22, 11·40)
Vitality	45·0 (30·0; 55·0)^c^	45·0 (25·0; 60·0)^c^	6·79 (4·80, 8·79)^c^	2·56 (0·84, 4·27)	3·78 (1·29, 6·28)
Emotional well-being	64·0 (48·0; 76·0)^c^	64·0 (48·0; 80·0)^c^	3·25 (1·38, 5·12)^c^	1·81 (0·08, 3·54)	1·17 (−1·22, 3·56)
Social functioning	62·5 (50·0; 87·5)^c^	62·5 (50·0; 87·5)^c^	5·22 (2·65, 7·79)^d^	−0·09 (−2·95, 2·76)	4·06 (0·56, 7·55)
Bodily pain	70·0 (45·0; 90·0)	77·5 (46·2; 90·0)	2·64 (0·15, 5·14)^c^	−1·61 (−3·84, 0·63)	3·69 (0·66, 6·71)
General health perceptions	55·0 (45·0; 70·0)^c^	60·0 (45·0; 73·8)^c^	2·12 (0·46, 3·78)^c^	−1·48 (−3·04, 0·08)	3·05 (0·88, 5·21)
**WPAI questionnaire scores**^b^	N = 384	N = 379	N = 244	N = 263	
Work time missed (absenteeism)^e^	0·0 (0·0; 0·0)	0·0 (0·0; 0·0)	−0·95 (−4·30, 2·41)	−0·69 (−3·59, 2·21)	0·26 (−3·00, 3·53)
Impairment while working (presenteeism)^e^	20·0 (0·0; 50·0)	20·0 (0·0; 57·5)	−10·08 (−15·19, −4·96)	−5·10 (−9·63, −0·57)	−5·65 (−11·00, −0·31)
Overall work impairment (productivity loss)^e^	20·0 (0·0; 50·0)	20·0 (0·0; 60·0)	−9·13 (−14·95, −3·31)	−4·64 (−9·31, 0·03)	−5·01 (−10·90, 0·89)
Impairment in regular activities (activity impairment)	20·0 (0·0; 50·0)	20·0 (0·0; 60·0)	−9·55 (−13·13, −5·97)	−4·26 (−7·66, −0·85)	−5·37 (−9·40, −1·34)

Values are presented as mean or between-group difference (95% confidence interval). Patient numbers indicate the smallest number of participants who completed each questionnaire/questionnaire dimension at each time point.

Abbreviations: MJM = mandibular jaw movements; PSG = polysomnography; QSQ = Quebec Sleep Questionnaire; SF-36 = Short Form 36; WPAI = Work Productivity and Activity Impairment.

a Higher scores indicate improvement.

b Lower scores indicate improvement.

c One value was missing.

d Two values were missing.

e These items relate only participants with a professional activity. The results are based on 236 to 243 responses in the MJM group, and 230 to 241 responses in the PSG group for baseline measurements, and on 120 to 131 responses in the MJM group, and 139 to 147 responses in the PSG group for change to baseline measurements.

Noninferiority in PAP adherence was not demonstrated (margin: 30 min), but the observed between-group difference was minimal (11·9 min; 95% CI: −32·8 to 56·6).

A post hoc analysis comparing MJM with at-home (rather than all) PSGs showed that median time to diagnosis (15 vs. 168 days; p < 0·01) and median time to treatment (50 vs. 162 days; p < 0·01) were significantly shorter in the MJM versus PSG (at-home) group (Supplementary Figure S5).

No adverse device effects were reported during the study.

## Discussion

In this randomised study of adults referred for suspected OSA, at-home MJM monitoring with AI-supported analysis was noninferior to PSG in reducing daytime sleepiness at 3 months post-diagnosis. OSA diagnosis was achieved 3 months earlier with MJM monitoring than with traditional PSG (despite no between-group difference in the time from the diagnostic testing date to the diagnostic consultation), and time to treatment initiation was also significantly shorter in the MJM group. Earlier access to therapy translated into a greater reduction in daytime sleepiness at 3 months post-randomisation in the MJM group. Exploratory secondary endpoints also supported the superiority of MJM monitoring over PSG at 3 months post-randomisation in improving health-related quality of life, work productivity, and daily activities, suggesting that the benefits of this approach extend beyond daytime sleepiness to broader quality-of-life outcomes. These benefits likely reflect earlier treatment initiation in the MJM group, as no significant between-group difference in daytime sleepiness was observed at 3 months after the diagnostic consultation. This result highlights the effectiveness of therapy and therefore the importance of early access to appropriate OSA treatment for improving symptoms and patient-reported outcomes. The minimal between-group difference in PAP device usage underscores that daytime sleepiness is only one of many factors affecting PAP adherence. Overall, study results support the clinical relevance of at-home MJM monitoring, which could contribute to evolution in OSA diagnostic approaches, including improved timeliness of treatment and its associated benefits.

Technological innovations to facilitate home sleep apnoea testing have the potential to revolutionise the way OSA is diagnosed in routine practice.^25^ Currently, the validation and assessment of these innovations is primarily focused on successful replication of indices provided by the ‘gold standard’ approach, namely single-night in-laboratory PSG. In that context, there is already robust evidence to support the performance of MJM monitoring compared with PSG.^17^^,^^18^^,^^26^^,^^27^ However, limiting the assessment of novel diagnostic tools to sensitivity and specificity is unlikely to convince healthcare workforces and payers of their clinical utility.^25^ It is also important to understand whether the new testing approaches speed up and expand access to treatment, improve equity and clinical outcomes. In addition, for a new diagnostic tool to have real-world impact, it must be easy to use and easily scalable across populations. These considerations were key drivers for the design of the current study, which evaluated time to diagnosis and treatment, along with patient-reported outcome measures. Although MJM analysis could take place over up to three nights, only the first night of recording was used for direct comparison with single-night PSG in the current analysis. MJM data from multiple nights (PSG was not repeated for feasibility reasons) will allow evaluation of night-to-night variability in AHI and OSA severity, a separate research objective that will be reported independently.

The current findings showing a shorter time to diagnosis and treatment with the use of at-home MJM monitoring are in line with early studies evaluating the impact of home sleep apnoea testing for OSA diagnosis.^28^^,^^29^ A 2022 systematic review investigated the effects of interventions to reduce wait times for diagnosis and treatment in adults with sleep-disordered breathing.^30^ While there was some indication that interventions to reduce wait times might have clinical benefits, available data were scarce and the authors called for further studies to fully evaluate the relationship between shorter wait times and patient outcomes.

There are also a limited number of randomised controlled trials comparing newer approaches to OSA diagnosis with PSG. Available data show that home sleep apnoea testing is as effective as PSG with respect to PAP adherence and outcomes such as daytime sleepiness, health-related quality of life, and the rate of cardiovascular events.^31^, ^32^, ^33^, ^34^, ^35^, ^36^ In addition, home sleep apnoea testing has been associated with reduced hospital resource utilisation and lower per-patient costs than PSG.^33^^,^^36^ However, the home testing methods evaluated in these previous trials were primarily based on polygraphy, and there were other differences from our study. The previous randomised trials included symptomatic individuals with a high pre-test probability of having OSA but excluded those with a high comorbidity burden. In contrast, around half of all patients with suspected OSA in routine practice are minimally symptomatic and often represent a middle-aged to elderly, multimorbid population.^37^ Our trial included patients referred for OSA-related symptoms or comorbidities, a group in which the prevalence of OSA is expected to be high, and reflecting routine practice around sleep apnoea diagnosis. Furthermore, several of the previous randomised trials investigated home sleep apnoea testing techniques in conjunction with automatically titrating PAP devices, bypassing in-laboratory titration.^31^^,^^35^ In contrast, the current study specifically focused on the impact of the diagnostic pathway itself, with therapeutic interventions to treat OSA left to the discretion of investigators.

OSA represents a major global societal health problem, with an estimated 300 million people in Europe remaining undiagnosed.^1^ There is therefore an unmet need to reduce sleep laboratory waiting lists and redefine diagnostic pathways for sleep apnea.^38^ A shorter home-based pathway can reduce drop-out rates by enabling faster access to care while avoiding the inconvenience and potential anxiety associated with the hospital environment. These advantages, meaningful for the individual patient, may translate into substantial population-level benefits by reducing sleep laboratory waiting times, allowing laboratory resources to be focused on the most complex cases. The goal is to deliver timely, personalised care for diagnosis and treatment. However, whether this approach will prove to be a cost-effective solution for diagnosing OSA likely depends on the healthcare setting and organisation of care. The impact of earlier diagnosis and treatment using MJM monitoring on long-term PAP adherence and objective clinical outcomes (e.g., rates of chronic conditions including cardiovascular disease), as well as the accumulation of real-world evidence to monitor sustained performance in everyday clinical settings, is another important area for future research.

Key strengths of the study include its large sample size, multicentre design, and pragmatic real-world setting. The approach taken to compare this innovative single-point end-to-end solution supported by AI analysis with both in-lab and at-home PSG, reflecting current practice in France for OSA diagnosis, is entirely novel. In addition, the study was conducted in 18 centres, including both public academic hospitals and private clinics, with no inclusion bias related to symptoms or comorbidities. Furthermore, the treatment of moderate-to-severe OSA with either PAP therapy or an oral appliance better reflects real-world clinical practice than focussing solely on PAP.

Alongside these strengths, the current findings should be interpreted in light of certain limitations. First, the follow-up duration was relatively short to evaluate the impact of using this new OSA diagnosis strategy on longer-term patient-reported and clinical outcomes, such as sustained changes in quality of life, and rates of cardiovascular events and mortality. Second, the study was conducted entirely in France, potentially limiting the generalisability of the findings to other healthcare systems. Nevertheless, the MJM monitoring device has already undergone independent evaluations outside France, supporting its use in multiple health systems. It has been granted an FDA De Novo classification and is eligible for reimbursement under the appropriate procedural codes. It is also recommended under the diagnostics guidance issued by the National Institute for Health and Care Excellence (NICE) for use within the UK National Health Service (NHS). Third, the study had an open-label design meaning that both investigators and patients were aware of study group allocation, which could have introduced response bias in patient-reported outcome measures or affected access to PSG. Other potential sources of bias relate to factors such as socioeconomic status and digital literacy. These were mitigated through standardised onboarding procedures, the availability of clinical support for device use, and eligibility criteria excluding participants unable to use a smartphone. Furthermore, given the size and randomised design of the trial, digital literacy issues would likely be expected to be relatively equally distributed across the two study arms. Fourth, the results comparing MJM analysis with at-home PSG only should be considered exploratory due to the post-hoc nature of this analysis. Additionally, although the ESS is a relevant outcome measure for patients, it only indirectly captures the effect of the diagnostic intervention. While the reduction in ESS score was statistically significantly greater in the MJM arm at 3 months post-randomisation, the between-group difference (−1·51 points) remained below the MCID of 2·5. Finally, we observed a higher rate of loss-to-follow-up than anticipated, with greater attrition in the PSG group, likely related to the longer waiting times for diagnosis. This imbalance between groups highlights how delays in diagnosis can increase the risk of drop-out. Although sensitivity analyses with imputed data supported the primary findings, the higher and imbalanced attrition rate means that the possibility of bias due to differential loss to follow-up, particularly for subjective outcomes such as ESS, cannot be fully excluded. In addition, such attrition and imbalance likely contributed to a loss of statistical power, even though the primary objectives were met. Finally, the non-inferiority analysis did not adjust for the ESS stratification factor used at randomisation. Although all analyses were conducted in accordance with the pre-specified statistical analysis plan, this was not aligned with the analyse-as-randomised principles, which should be considered when interpreting the findings.

In conclusion, our results show that an OSA diagnosis strategy based exclusively on at-home MJM monitoring with AI-supported analysis was noninferior to PSG in reducing daytime sleepiness, while significantly accelerating time to diagnosis and treatment initiation, resulting in earlier improvement in daytime sleepiness.

## Contributors

JLP and MR designed the study. KD participated in trial management. The statistical analysis plan was written by MR and MM. The Grenoble academic team (JLP, MM, and MR) accessed and verified the data. Statistical analyses were conducted by MM and MR (independent academic statisticians) independently of the Sunrise company. Primary outcome analyses were checked by an independent statistical team at the University of Grenoble. Planning for the first draft of the manuscript was undertaken by JLP and KD. The first draft of the paper was written by JLP, MR, and KD with the editorial assistance of an independent medical writer, whose support was funded by the Grenoble academic team. All authors contributed to critical revisions of the manuscript, vouch for the fidelity of the trial to the protocol, and approved the decision to submit for publication. JLP acts as overall guarantor and accepts full responsibility for the work and the conduct of the study.

## Data sharing statement

Deidentified participant data will be made available upon reasonable request to the corresponding author subject to agreement of the study sponsor. Access will be granted to researchers with an approved scientific proposal including specified objectives. Data will only be shared after a data sharing agreement is fully executed.

## Declaration of interests

JLP is supported by the French National Research Agency (ANR) in the framework of the “FRANCE 2030” program, the “e-health and integrated care” chair of Grenoble Alpes University Foundation, and the “Sleep Health-AI chair” within the “MIAI Cluster” for artificial intelligence (ANR-23-IACL-0006). He also reports consulting fees from Resmed, Sefam, Zoll-Respicardia, Eli Lilly, Idorsia, Pharmanovia, Biosency, and Bioprojet; and support for attending meetings from Agiradom. RT is supported by the French National Research Agency (ANR) in the framework of the “FRANCE 2030” program, CDP “my way to health” (ANR-23-IACL-0006). He also reports consulting fees from Resmed, Idorsia and Bioprojet; payment or honoraria for lectures, presentations, or educational events from Bioproject, Resmed, Idorsia, Inspire Medical and Elivie; grant support through his institution from Bioprojet; advisory board participation for Bioproject, Resmed and Inspire Medical; and support for attending meetings from Agiradom. KD is an employee of Sunrise. AP reports consulting fees from Resmed; payment or honoraria for lectures, presentations, or educational events from Resmed, Elia Medical, VitalAire, SOS Oxygène, Asten Santé, and ISIS Médical; and support for attending meetings or travel from Elia Medical, ISIS Médical, SOS Oxygène and ALMS. MP reports consulting fees from Philips Respironics, Air Liquide Medical, Resmed, SRETT, Asten Santé, and GSK; payment or honoraria for lectures, presentations, or educational events from Philips Respironics, Asten Santé, Resmed, Air Liquide Medical, SOS Oxygène, Antadir, Chiesi, Jazz Pharmaceuticals, Löwenstein Medical, Fisher & Paykel, Bastide Medical, Orkyn, Elivie, and Sanofi; support for attending meetings from Asten Santé, VitalAire, and Sanofi; advisory board participation for Resmed, Sanofi, Philips Respironics, AstraZeneca, and Asten Santé; receipt of equipment from Philips Respironics, Resmed, and Fisher & Paykel; research grants from Asten Santé, Resmed and Fisher & Paykel; and shares in Kernel Biomedical. FG reports payment or honoraria for lectures, presentations, or educational events from Resmed, Philips Respironics, Bioprojet, Inspire Medical, Sefam, and Cidelec; advisory board participation from Sefam, Air Liquide Santé, Inspire Medical, Bioprojet, Eli Lilly and Asten Santé; and meeting or travel support from Resmed, Asten Santé, Sefam, and Inspire Medical. JBM is a scientific advisor to Sunrise and is an investigator in pharmaceutical trials for Jazz Pharmaceuticals, SMB Lab, Takeda and Alkermes. HP, MM, and MR have no conflicts of interest to declare.

## Appendix A Supplementary data

Supplementary Appendix

Study protocol

Statistical analysis plan

SUNSAS study investigators

French translation of the abstract
