# Supplementary material for: Time to diagnosis and treatment of obstructive sleep apnoea using mandibular jaw movement monitoring versus polysomnography: an open-label, multicentre, randomised, controlled trial
Source: Lancet Reg Health Eur. 2026 Mar 17;64:101637. doi: 10.1016/j.lanepe.2026.101637 (PMC13147807; doi:10.1016/j.lanepe.2026.101637)
Supplement: Supplementary Appendix [file mmc1.docx]

**Supplementary Appendix**

**Time to diagnosis and treatment of obstructive sleep apnoea using mandibular jaw movement monitoring versus polysomnography: an open-label, multicentre, randomised, controlled trial**

Jean-Louis Pépin MD, Renaud Tamisier MD, Marc Manceau PhD, Katleen Denoncin PhD, Arnaud Prigent MD, Maxime Patout MD, Hervé Pégliasco MD, Frédéric Gagnadoux MD, Jean-Benoit Martinot MD, Matthieu Roustit PhD, on behalf of SUNSAS investigators.

From the University Grenoble Alpes, HP2, Inserm 1300, Grenoble, France (JLP, RT, MM, MR); Grenoble Alpes University Hospital, EFCR Laboratory, Grenoble, France (JLP, RT); Sunrise, Namur, Belgium (KD); Polyclinique Saint-Laurent, Rennes, France (AP); La Pitié Salpêtrière University Hospital, Pulmonology and Sleep Department, FHU UMANHYS, Sorbonne University, Paris, France (MP); Hôpital Européen, Marseille, France (HP); Department of Respiratory and Sleep Medicine, Angers University Hospital, Angers, France (FG); Sleep Laboratory, CHU Université catholique de Louvain (UCL), Namur Site Sainte-Elisabeth, Namur, Belgium (JBM); and Institute of Experimental and Clinical Research, UCL Bruxelles Woluwe, Brussels, Belgium (JBM).

This appendix has been provided by the authors to give readers additional information about the work.

Table of Contents

[List of SUNSAS Principal Investigators 2](#_Toc221281244)

[Supplementary Methods 3](#_Toc221281245)

[Inclusion Criteria 3](#_Toc221281246)

[Exclusion Criteria 3](#_Toc221281247)

[Assessments 3](#_Toc221281248)

[Supplementary Figures 4](#_Toc221281249)

[Figure S1. Details of the mandibular jaw movement (MJM) monitoring device combined with artificial intelligence-based automated analysis. 4](#_Toc221281250)

[Figure S2. Trial design schematic. 5](#_Toc221281251)

[Figure S3. Hierarchical statistical analysis (global alpha risk control) strategy. 6](#_Toc221281252)

[Figure S4. Kaplan-Meier curves showing time from diagnostic testing (mandibular jaw movement (MJM) monitoring or polysomnography (PSG)) to diagnosis consultation. 7](#_Toc221281253)

[Figure S5. Kaplan-Meier curves showing time from randomisation to diagnosis (upper panel) and from randomisation to treatment initiation (lower panel) in the mandibular jaw movement (MJM) monitoring and at-home polysomnography (PSG) groups – a post-hoc analysis. 8](#_Toc221281254)

[Supplementary Tables 9](#_Toc221281255)

[Table S1. Representativeness of the study population. 9](#_Toc221281256)

[References 10](#_Toc221281257)

# List of SUNSAS Principal Investigators

| **Principal Investigator** | **Affiliation** |
| --- | --- |
| Jean-Louis PEPIN | Clinique Universitaire de Physiologie, Pôle Thorax et Vaisseaux, CHU Grenoble Alpes and Université Grenoble Alpes, Grenoble, France |
| Yves DAUVILLIERS | CHU Montpellier – Hôpital Gui-de-Chauliac, Unité des Troubles du Sommeil, Département de Neurologie, Montpellier, France |
| Marie Pia D’ORTHO | Hôpital Bichat-Claude Bernard, Service d’Explorations Fonctionnelles Multidisciplinaires, Physiologie, Centre du Sommeil, Paris, France |
| Frédéric GAGNADOUX | CHU Angers, Département de Pneumologie et Médecine du Sommeil, Angers, France |
| Thibaut GENTINA | Hôpital privé la Louvière, CESAL - Centre d’Explorations du Sommeil de l’Agglomération Lilloise, Lille, France |
| Damien LÉGER | AP-HP Hôpital Hôtel Dieu, Centre du Sommeil et de la Vigilance – Centre de Référence Narcolepsies et Hypersomnies Rares, Service de Pathologies Professionnelles et Environnementales, Paris, France |
| Herve PEGLIASCO | Hôpital Européen Marseille, Service de Pneumologie, Marseille, France |
| Laure PETER-DEREX | CHU Lyon – Hôpital de la Croix-Rousse, Centre de Médecine du Sommeil et des Maladies Respiratoires, Lyon, France |
| Pierre PHILIP | CHU Bordeaux – Hôpital Pellegrin, Service Universitaire de Médecine du Sommeil, Service des Explorations Fonctionnelles du Système Nerveux - Clinique du Sommeil, Bordeaux, France |
| Carole PLANES | Hôpitaux Universitaires de Paris Seine Saint-Denis (HUPSSD, AP-HP), Hôpital Avicenne, Service de Physiologie - Explorations Fonctionnelles, Bobigny, France |
| Arnaud PRIGENT | Polyclinique Saint-Laurent, Groupe Médical de Pneumologie, Rennes, France |
| Laurent BOYER | AP-HP Hôpital Henri Mondor, Service de Physiologie Explorations Fonctionnelles, Créteil, France |
| Vincent PUEL | Pôle Exploration Apnées Sommeil (PEAS), Nouvelle Clinique Bel-Air, Bordeaux, France |
| Maxime PATOUT | Service des Pathologies du Sommeil (Département R3S), AP-HP Pitié Salpêtrière, Groupe Hospitalier Universitaire APHP-Sorbonne Université, Paris, France |
| Ari CHAOUAT | Centre Hospitalier Régional Universitaire de Nancy, Département de Pneumologie, Hôpital d’Adultes de Brabois, Vandoeuvre-lès-Nancy, France |
| Claire LAUNOIS | Service des Maladies Respiratoires, Centre Hospitalier Universitaire de Reims, Hôpital Maison Blanche, Reims, France |
| Sandrine PONTIER-MARCHANDISE | Centre Hospitalier Universitaire de Toulouse, Hôpital Larrey, Toulose, France |
| Renaud TAMISIER | Centre du Sommeil de Grenoble, Grenoble, France |

# Supplementary Methods

## Inclusion Criteria

A patient must meet all of the following inclusion criteria to be enrolled in the study:

- Male or female aged 18 to 80 years;
- Patient referred for suspected sleep apnoea syndrome;
- Patient with a smartphone and home internet access, and able to use a smartphone application;
- Patient affiliated with or beneficiary of a social security scheme.

## Exclusion Criteria

A patient must not meet any of the following criteria to be included in the study:

- Patient who has already undergone any type of sleep recording test within the five years preceding inclusion;
- Patient already treated for OSA (Obstructive Sleep Apnoea) within the five years preceding inclusion;
- Patient with a severe chronic obstructive or restrictive pulmonary disease, with or without oxygen therapy (as judged by the principal investigator);
- Patient who refuses to shave their beard (if too thick) to wear the Sunrise device;
- Patient with unstable cardiovascular disease or severe heart failure requiring hospitalization within the three months preceding inclusion, or classified as Class III or IV according to the New York Heart Association;
- Individuals referred to in Articles L1121-5 to L1121-8 of the French Public Health Code (pregnant women, women in labor, breastfeeding mothers, persons deprived of liberty by judicial or administrative decision, persons under legal protection — may not be included in clinical trials);
- Patient currently excluded from another study or currently participating in an interventional study;
- Patient who, in the investigator’s opinion, is likely to be uncooperative or non-compliant with the obligations of participating in the study;
- Patient with pathologies affecting the rotation of the condyle in the temporomandibular joint; Patient with priority diagnostic needs due to high-risk occupations (e.g., truck drivers, night workers, etc.).

## Assessments

The Short Form-36 (SF-36) is a generic patient-reported outcome measure that quantifies health status and measures health-related quality of life (HRQoL) across eight domains (physical functioning, role limitations due to physical health problems, bodily pain, general health perceptions, vitality [energy/fatigue], social functioning, role limitations due to emotional problems, and mental health).^1^ A total SF-36 score above 50 indicates above-average HRQoL, while a score below 50 suggests below-average HRQoL.

The Quebec Sleep Questionnaire (QSQ) Is a disease-specific HRQoL instrument designed to assess the effect of obstructive sleep apnoea (OSA) on quality of life.^2^ It includes 32 items across five domains (hypersomnolence, diurnal symptoms, nocturnal symptoms, emotions, and social interactions), each of which is scored on a 7-point Likert scale, where higher scores indicate better disease-specific quality of life.

The Epworth Sleepiness Scale (ESS) is a self-assessment tool comprising eight items, where individuals rate their usual chances of dozing off during various daytime scenarios. Each situation is scored on a scale from 0 (no likelihood of dozing) to 3 (high likelihood of dozing). The total ESS score is calculated by summing the scores from all eight items, yielding a possible score of 0 to 24, with higher scores reflecting increased levels of daytime sleepiness. An ESS score above 10 indicates the presence of excessive daytime sleepiness.

The Work Productivity and Activity Impairment Questionnaire (WPAI) is used to evaluate the impact of health problems on an individual’s work and daily activities over the previous 7 days. It measures absenteeism, presenteeism, and activity impairment. Higher scores indicate greater impairment and decreased productivity.

# Supplementary Figures

Figure S1. Details of the mandibular jaw movement (MJM) monitoring device combined with artificial intelligence-based automated analysis. Sunrise (Sunrise, Namur, Belgium) is a home sleep test consisting of a sensor, smartphone application, and cloud-based software that analyses sleep data and delivers a report that is available to clinicians. To measure physiological parameters during sleep, the device captures MJMs using a lightweight, single-point sensor placed on the chin. The sensor’s inertial measurement unit includes a gyroscope and an accelerometer, which respectively record the movement and position of the mandible across three axes. Recorded data are wirelessly transmitted to the smartphone application. Once the recording is complete, the full dataset is transferred from the smartphone to the cloud for analysis by dedicated software. Using artificial intelligence, the system identifies specific MJM signal patterns associated with physiological or pathological events, such as sleep stages, arousals, and respiratory disturbances. The resulting clinical scores are compiled into a comprehensive report accessible through an online portal, where clinicians can review, annotate, and edit both the report and the raw data.


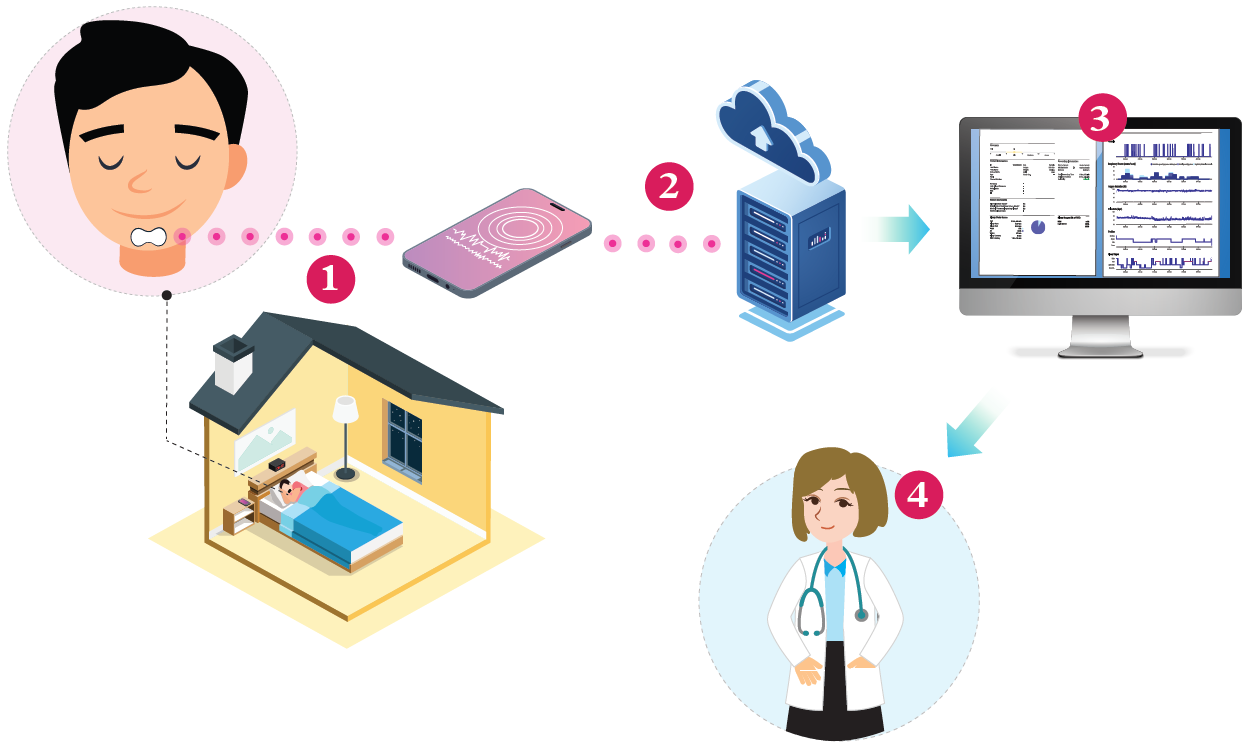


Figure S2. Trial design schematic. The SUNSAS study was a multicentre, prospective, randomised, controlled trial with two open-label arms. After providing informed consent, eligible participants were randomised to one of two specific diagnosis pathways. In the polysomnography (PSG) arm, diagnosis was based on in-laboratory or in-home overnight PSG, according to each centre’s standard practice. In the mandibular jaw movements (MJM) arm, diagnosis was based on MJM monitoring with artificial intelligence-supported analysis at home. After the diagnostic procedure, participants attended a diagnostic consultation to review results, discuss treatment options, and receive a therapy prescription if indicated. Follow-up visits were then conducted at 3 months post-randomisation and 3 months post-diagnosis for all participants, and at 3 months post-treatment initiation for patients diagnosed and treated for obstructive sleep apnoea (OSA).


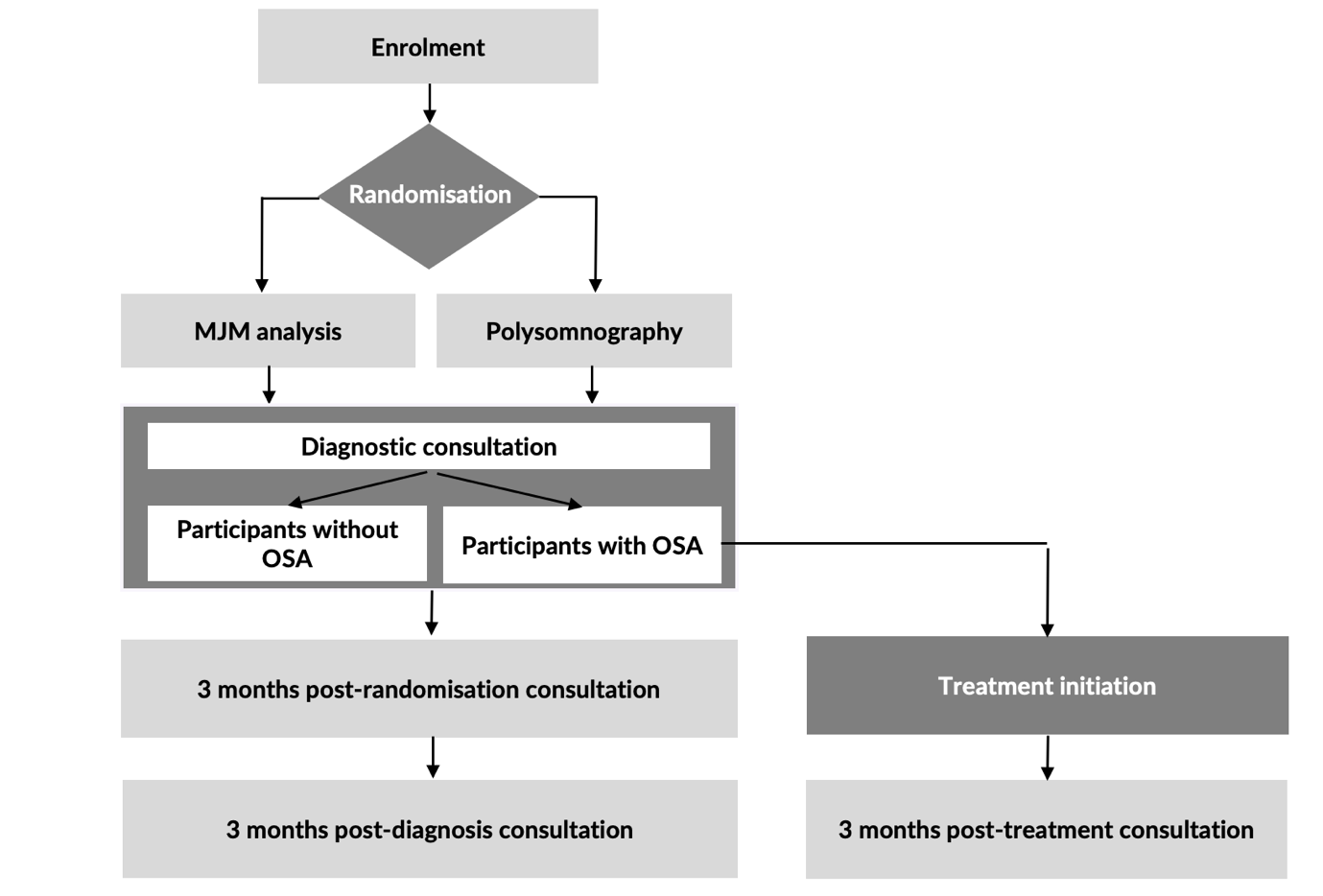


Figure S3. Hierarchical statistical analysis (global alpha risk control) strategy. The first level of the hierarchical statistical testing assessed two hypotheses: the noninferiority of the new mandibular jaw movements (MJM)-based diagnostic home sleep test compared with polysomnography (PSG) for reducing daytime sleepiness (measured by the Epworth Sleepiness Scale [ESS] score) at 3 months post-diagnosis, and the superiority of the new diagnostic test over PSG for the time from randomisation to diagnosis. Both null hypotheses needed to be rejected before testing the second level, which tested the superiority of the new test over PSG for time from randomisation to treatment initiation in patients with an indication for OSA treatment. If the null hypothesis was rejected, the third level tested the superiority of the new test over PSG for reducing the ESS score at 3 months post-randomisation. mITT, modified intention-to-treat.

Figure S4. Kaplan-Meier curves showing time from diagnostic testing (mandibular jaw movement (MJM) monitoring or polysomnography (PSG)) to diagnosis consultation.


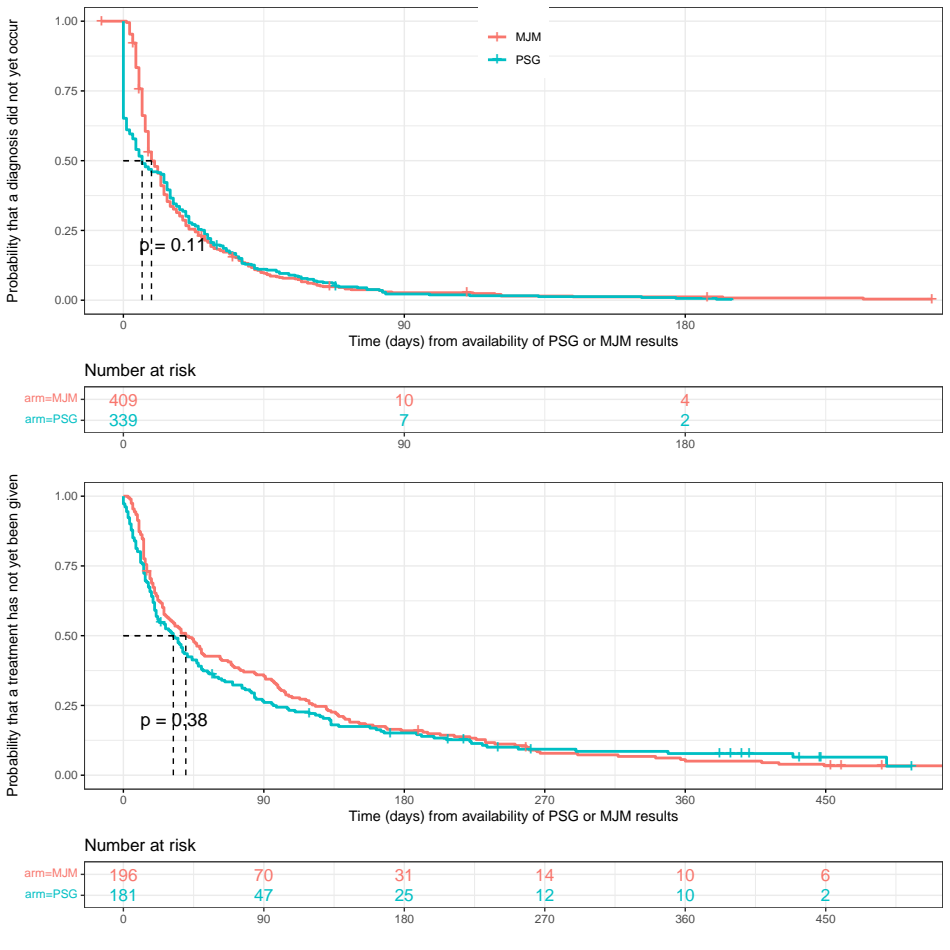


Figure S5. Kaplan-Meier curves showing time from randomisation to diagnosis (upper panel) and from randomisation to treatment initiation (lower panel) in the mandibular jaw movement (MJM) monitoring and at-home polysomnography (PSG) groups – a post-hoc analysis.


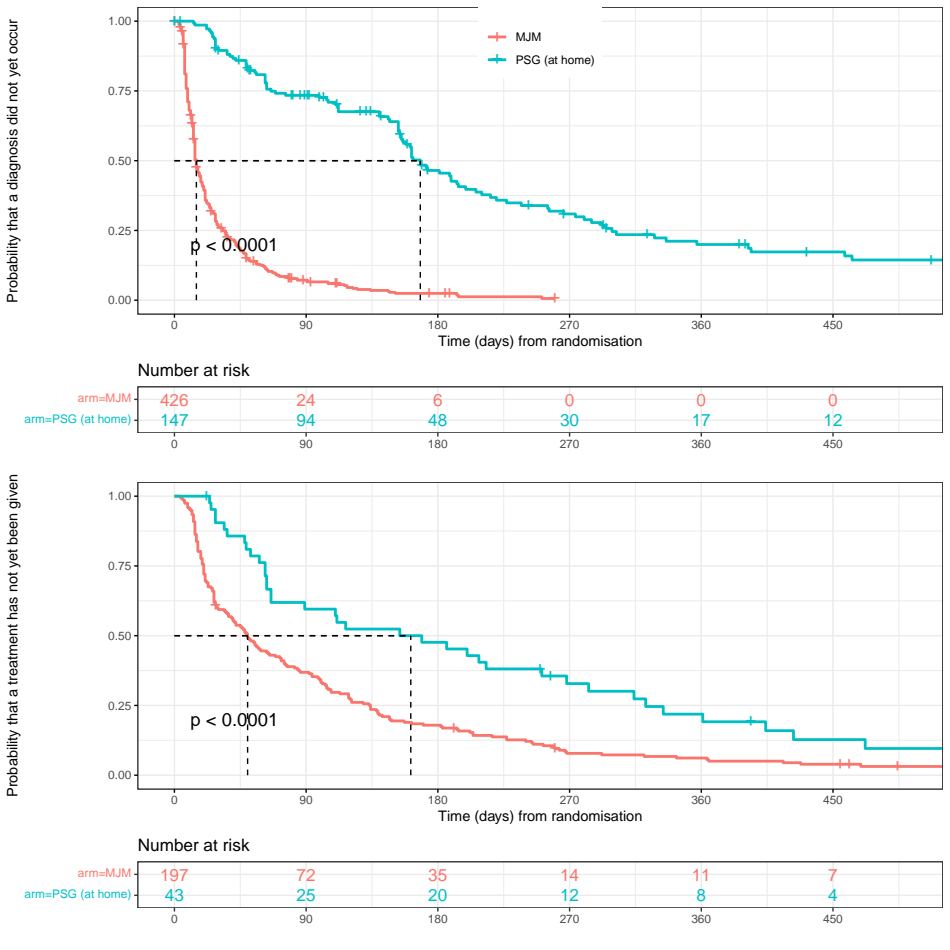


# Supplementary Tables

## Table S1. Representativeness of the study population.

| **Category** | **Details** |
| --- | --- |
| **Disease under investigation** | Obstructive sleep apnoea (OSA) |
| **Sex and gender** | OSA is more prevalent in men than in women. A seminal population-based study from the early 1990s reported that OSA (defined as an apnoea-hypopnea index [AHI] ≥5 events/h with symptoms) affected 4% of men and 2% of women.^3^ More recent studies, likely influenced by rising obesity rates, estimate OSA prevalence between 14% and 50% in men and 5% to 23% in women,^4,5^ suggesting a male-to-female ratio typically ranging from 2:1 to 5:1. Findings from a large population-based study estimate the overall prevalence of OSA in the French adult population aged 18–69 years at 20·9%, with higher rates in men (24·1%) than in women (18·0%).^6^ |
| **Age** | The prevalence of OSA increases with age. In France, estimated OSA prevalence is 25·8% in adults aged <40 years, rising to 39·1% in those aged 40–49 years, 44·7% in those aged 50–59 years, and 44·0% in individuals aged ≥60 years.^6^ |
| **Race or ethnic group** | OSA appears to be more prevalent in certain racial and ethnic groups, including African Americans, Hispanics, and Native Americans. |
| **Geography** | OSA is a significant global public health issue due to its high and rising prevalence. According to estimates by Benjafield et al. (2019),^7^ over one billion people aged 30–69 years worldwide are affected by OSA. The countries with the highest absolute numbers of individuals with OSA are China, the United States, Brazil, and India. Other countries ranking in the top ten include Pakistan, Russia, Nigeria, Germany, France, and Japan. This distribution largely mirrors the population size of these countries, highlighting the global burden of the disorder and the need for widespread screening and management strategies. |
| **Other considerations** | While male sex, older age, and obesity are established risk factors for OSA, additional behavioural and socioeconomic determinants may also contribute, though their roles are less well defined. |
| **Overall representativeness of the study** | The SUNSAS study was conducted in adults ≥18 years in France. Just over half of the study population (56%) were male and the median age was 50·0 years. These demographic characteristics are consistent with known epidemiological patterns of OSA, which is more prevalent in middle-aged adults and shows a higher incidence in men than women. The study had a broad recruitment strategy, enrolling 849 patients from 18 sleep centres across France, spanning both public hospitals and private clinics. This multicentre approach enhances the external validity of the findings by capturing a diverse and representative sample of the French adult population affected by OSA. The inclusion of patients from various healthcare settings ensures representativity in terms of geographic, socioeconomic, and clinical pathways, making the results highly generalisable to real-world practice. Of the 774 individuals with suspected OSA who underwent a diagnostic consultation in the context of the SUNSAS study, 404 (52%) were prescribed treatment with either an oral appliance or positive airway pressure. |

# References

1. Ware JE, Jr., Sherbourne CD. The MOS 36-item short-form health survey (SF-36). I. Conceptual framework and item selection. *Med Care*. 1992;30(6):473-83.

2. Lacasse Y, Bureau MP, Sériès F. A new standardised and self-administered quality of life questionnaire specific to obstructive sleep apnoea. *Thorax*. 2004;59(6):494-9. doi:10.1136/thx.2003.011205

3. Young T, Palta M, Dempsey J, Skatrud J, Weber S, Badr S. The occurrence of sleep-disordered breathing among middle-aged adults. *N Engl J Med*. 1993;328(17):1230-5. doi:10.1056/nejm199304293281704

4. Heinzer R, Vat S, Marques-Vidal P, et al. Prevalence of sleep-disordered breathing in the general population: the HypnoLaus study. *Lancet Respir Med*. 2015;3(4):310-8. doi:10.1016/s2213-2600(15)00043-0

5. Peppard PE, Young T, Barnet JH, Palta M, Hagen EW, Hla KM. Increased prevalence of sleep-disordered breathing in adults. *Am J Epidemiol*. 2013;177(9):1006-14. doi:10.1093/aje/kws342

6. Balagny P, Vidal-Petiot E, Renuy A, et al. Prevalence, treatment and determinants of obstructive sleep apnoea and its symptoms in a population-based French cohort. *ERJ Open Res*. 2023;9(3)doi:10.1183/23120541.00053-2023

7. Benjafield AV, Ayas NT, Eastwood PR, et al. Estimation of the global prevalence and burden of obstructive sleep apnoea: a literature-based analysis. *Lancet Respir Med*. 2019;7(8):687-698. doi:10.1016/s2213-2600(19)30198-5
