# Supplementary material for: Time to diagnosis and treatment of obstructive sleep apnoea using mandibular jaw movement monitoring versus polysomnography: an open-label, multicentre, randomised, controlled trial
Source: Lancet Reg Health Eur. 2026 Mar 17;64:101637. doi: 10.1016/j.lanepe.2026.101637 (PMC13147807; doi:10.1016/j.lanepe.2026.101637)
Supplement: Study protocol [file mmc2.pdf]

CLINICAL INVESTIGATION PLAN

Validation of an integrated digital solution (Sunrise device) for the automated analysis of mandibular jaw movements using artificial intelligence versus polysomnography for the diagnosis of obstructive sleep apnea: a national multicenter randomized controlled trial

SUNSAS

Prospective controlled, randomized, parallel-arm, open-label, multicenter, national study

N° ID/RCB : 2021-A01827-34

N° protocol, version and date : **PRO-545 V1.7 - 13 June 2022**

**Sponsor**	Sunrise SA<br>Chaussée de Marche 598/02, 5101 Namur, Belgium<br>Tel: +32 81 26 11 26<br>Mail: support@hellosunrise.com
**Coordinating Investigator**	Prof. Jean-Louis Pépin<br>CHU Grenoble Alpes<br>Clinique Universitaire de Physiologie, sommeil et exercice<br>Pôle Thorax et Vaisseaux,<br>Boulevard de la chantourne<br>38700 La Tronche<br>France<br>Tel: +33 4 76 76 87 66<br>Mail: JPepin@chu-grenoble.fr

TRANSLATED VERSION FROM FRENCH TO ENGLISH USING AI

« This document is the exclusive property of SUNRISE SA. It may not be copied, reproduced, or disclosed, in whole or in part, without its written authorization. »

REVISION HISTORY

VERSION	DATE	MAJOR MODIFICATIONS
1.0	09/05/2020	N/A (submission of the protocol to HAS)
1.1	10/10/2020	Modification of the primary endpoint as required by HAS
1.2	24/11/2020	Minor protocol modifications following observations from the HAS College (opinion no. 2020.0072/AC/SED dated November 12, 2020)
1.3	19/01/2021	Modification of the list of investigative centers participating in the study
1.4	15/06/2021	Initial submission to the CPP including the following modifications:<br>• Update of key stakeholders (addition of the sponsor's representative, the CRO, contact details of the centers, and data management)<br>• Addition of the methodologist's signature on the protocol signature page<br>• Addition of the investigators' signature page<br>• Update of abbreviations<br>• Clarification of endpoint no. 5 (PAP compliance over the last 30 days of use)<br>• Addition of time windows for follow-up and telephone visits (+/- 7 days) and the possibility to combine visits if the windows overlap<br>• Removal of the 6-month post-randomization visit<br>• Telephone follow-up at 3 months post-diagnosis replaced by a hospital visit<br>• Addition of the non-inclusion criterion: "Patient suffering from conditions affecting condyle rotation in the temporomandibular joint," in reference to the contraindication stated in the instructions for use<br>• Update of various protocol sections to comply with ISO 14155:2020 standard
1.5	16/08/2021	• Update of the vigilance section following requests from ANSM and CPP Île-de-France IV<br>• Modification of section 23 regarding overall study supervision (clarification on the objectives and composition of the steering committee)<br>• Change of principal investigator at the Nouvelle Clinique Bel-Air center<br>• Change of the sponsor's and manufacturer's address due to recent relocation<br>• Update of contact details for Nathalie Préaubert

1.6	17/11/2021	• The principal investigator at CHRU Lille was replaced (Dr. Gaudier replaces Prof. Charley-Monaca)<br>• The ESS questionnaire is also completed at the 3-month post-treatment follow-up visit<br>• Adverse event and defect reporting to be done via paper forms instead of the eCRF as initially indicated<br>• Protocol deviations to be reported using an Excel form instead of via the eCRF as initially indicated<br>• Clarifications regarding objectives at 3 months post-diagnosis (i.e., 3 months after the diagnostic announcement consultation/teleconsultation, not 3 months after the diagnostic procedure)<br>• Visit windows modified to +/- 15 days instead of +/- 7 days<br>• Diagnostic announcement and 3-month post-treatment follow-up visits may be conducted in person or via teleconsultation depending on the usual practice of the centers<br>• In addition to collecting treatments at inclusion, treatments will also be reviewed with the patient during each visit<br>• For follow-up visits, self-questionnaires completed by patients may be filled out electronically up to 10 days before or after the visit date<br>• Questionnaires completed routinely prior to consent do not need to be repeated if completed within 6 weeks (instead of 15 days) before the inclusion visit, unless deemed necessary by the investigator<br>• Statistical clarifications on the 3-month post-diagnosis analysis if the patient's follow-up reaches the maximum 18-month period<br>• Typo corrections (postal code for Marseille, name of the WPAI:SAS questionnaire)<br>• Update of the Sunrise device label following the address change of SUNRISE SA
1.7	13/06/2022	• Follow-up visits at 3 months after inclusion and 3 months after the diagnostic announcement consultation may be conducted in person or via teleconsultation, depending on the usual practice at each center<br>• Addition of new centers<br>• Update of the inclusion period

PRINCIPAL STAKEHOLDERS

**Sponsor**	**SUNRISE SA**<br>**Laurent et Pierre MARTINOT**<br>Chaussée de Marche 598/02, 5101 Namur, Belgium<br>Tel : +32 81 26 11 26<br>Mail : laurent@hellosunrise.com et pierre@hellosunrise.com
**Sponsor Representative**	**Délégation à la Recherche Clinique et à l'Innovation**<br>CHU GRENOBLE Alpes, Pavillon Dauphiné,<br>CS 10217, 38043 GRENOBLE Cedex 09, France<br>Chef de projet Promoteur : Amjad UNEISI<br>Tel : +33 4 76 76 81 08<br>Mail : AUneisi@chu-grenoble.fr
**CRO**	**ICUREsearch**<br>6B avenue de Romans<br>38160 Saint-Marcellin, France<br>Tel : +33 4 38 90 39 38<br>Mail : stephane.ruckly@icuresearch.eu
**Coordinating Investigator**	Prof. Jean-Louis Pépin<br>CHU Grenoble Alpes<br>Clinique Universitaire de Physiologie, sommeil et exercice<br>Pôle Thorax et Vaisseaux,<br>Boulevard de la chantourne<br>38700 La Tronche<br>France<br>Tel: +33 4 76 76 87 66<br>Mail: JPepin@chu-grenoble.fr
**Principal Investigators**	**Dr Laurent BOYER**<br>AP-HP Hôpital Henri Mondor<br>Service de physiologie explorations fonctionnelles<br>1 Rue Gustave Eiffel<br>94000 Créteil, France<br>Tel : +33 1 49 81 26 77<br>Mail : laurent.boyer@aphp.fr<br><br>**Pr Yves DAUVILLIERS**<br>CHU Montpellier – Hôpital Gui-de-Chauliac<br>Unité des troubles du sommeil, département de neurologie<br>80 Av Augustin Fliche<br>34295 Montpellier Cedex 5, France<br>Tel : +33 4 67 33 63 61<br>Mail : y-dauvilliers@chu-montpellier.fr

	**Pr Marie Pia d'ORTHO**<br>AP-HP Hôpital Bichat-Claude Bernard<br>Service d'explorations fonctionnelles multidisciplinaires, physiologie, centre du sommeil<br>46 Rue Henri Huchard<br>75018 Paris, France<br>Tel : +33 1 40 25 84 01<br>Mail : marie-pia.dortho@bch.aphp.fr<br><br>**Pr Frédéric GAGNADOUX**<br>CHU Angers<br>Département de pneumologie et médecine du sommeil<br>4 rue Larrey<br>Angers, 49933 cedex 9, France<br>Tel: +33 2 41 35 36 95<br>Mail: frgagnadoux@chu-angers.fr<br><br>**Dr Thibaut GENTINA**<br>Hôpital privé la Louvière<br>CESAL - Centre d'Explorations du Sommeil de l'Agglomération Lilloise<br>69 Rue de la Louvière<br>59800 Lille, France<br>Tel : +33 3 20 55 02 50<br>Mail : docteur.gentina@gmail.com<br><br>**Pr Damien LÉGER**<br>AP-HP CUP Hôpital Hôtel Dieu<br>Centre du sommeil et de la vigilance – centre de référence narcolepsies et hypersomnies rares, service de pathologies professionnelles et environnementales<br>1 pl. Parvis Notre-Dame<br>75181 PARIS CEDEX, France<br>Tel : +33 1 42 34 85 63<br>Mail : damien.leger@aphp.fr<br><br>**Dr Simon Gaudier**<br>CHRU Lille – Hôpital Roger Salengro<br>Unité des troubles de la veille et du sommeil, neurophysiologie clinique<br>Rue du Pr Émile Lainé<br>59037 Lille cedex, France<br>Tel : +33 3 20 44 63 62<br>Mail : simon.gaudier@CHRU-LILLE.FR<br><br>**Dr Herve PEGLIASCO**<br>Hôpital Européen Marseille<br>Service de pneumologie<br>6 rue Désirée Clary

	13003 Marseille, France<br>Tel : +33 4 13 42 72 40<br>Mail : H.PEGLIASCO@hopital-europeen.fr<br><br>**Dr Laure PETER- DEREX**<br>CHU Lyon – Hôpital de la Croix-Rousse<br>Centre de Médecine du Sommeil et des Maladies Respiratoires<br>103 Grande Rue de la Croix-Rousse<br>69004 Lyon, France<br>Tel : +33 4 72 07 17 69<br>Mail : laure.peter-derex@chu-lyon.fr<br><br>**Pr Pierre PHILIP**<br>CHU Bordeaux – Hôpital Pellegrin<br>Service Universitaire de Médecine du Sommeil<br>Service des explorations fonctionnelles du système nerveux - Clinique du sommeil<br>Place Amélie Raba-Léon<br>33076 Bordeaux Cedex, France<br>Tel : +33 5 57 82 01 73<br>Mail : pierre.philip@chu-bordeaux.fr<br><br>**Dr Carole PLANES**<br>Hôpitaux Universitaires de Paris Seine Saint-Denis (HUPSSD, AP-HP), Hôpital Avicenne<br>Service de Physiologie - Explorations fonctionnelles<br>125, rue de Stalingrad<br>93000 Bobigny, France<br>Tel : +33 1 48 95 56 37<br>Mail : carole.planes@aphp.fr<br><br>**Dr Arnaud PRIGENT**<br>Polyclinique Saint-Laurent<br>Groupe médical de pneumologie<br>2 Ter, rue de Saint-Laurent<br>35700 Rennes, France<br>Tel : +33 2 99 25 65 35<br>Mail : dr.arnaudprigent@gmail.com<br><br>**Dr Vincent PUEL**<br>Pôle Exploration Apnées Sommeil (PEAS), Nouvelle clinique Bel-Air<br>138 av de la République<br>33073 BORDEAUX CEDEX, France<br>Tel : +33 6 80 24 12 70<br>Mail : vpuel001@gmail.com<br><br>**Dr Maxime PATOUT**

	Service des Pathologies du Sommeil (Département R3S)<br>AP-HP Pitié Salpêtrière, Groupe Hospitalier Universitaire APHP-Sorbonne Université,<br>47-96 Boulevard de l'hôpital<br>75013 Paris, France<br>Tel : +33 6 63 79 23 28<br>Mail : maxime.patout@aphp.fr<br><br>**Pr Ari CHAOUAT**<br>Centre Hospitalier Régional Universitaire de Nancy<br>Département de Pneumologie<br>Hôpital d'Adultes de Brabois<br>Allée du Morvan<br>54500 Vandoeuvre-lès-Nancy, France<br>Tel : +33 3 83 15 40 21<br>Mail : a.chaouat@chru-nancy.fr<br><br>**Dr Claire LAUNOIS**<br>Service des Maladies Respiratoires<br>Centre Hospitalier Universitaire de Reims<br>Hôpital Maison Blanche<br>45 rue Cognacq Jay<br>51092 Reims, France<br>Tel : +33 3 26 78 76 14<br>Mail : claunois@chu-reims.fr<br><br>**Dr Sandrine PONTIER – MARCHANDISE**<br>Centre Hospitalier Universitaire de Toulouse<br>Hôpital Larrey<br>24 chemin de Pouvourville<br>31059 Toulouse, France<br>Tel : +33 5 67 77 18 46<br>Mail : pontier.s@chu-toulouse.fr<br><br>**Pr Renaud TAMISIER**<br>Centre du Sommeil de Grenoble<br>75 Avenue Gabriel Péri<br>38400 Saint Martin d'Hères, France<br>Tel : +33 4 76 76 84 69<br>Mail : rtamisier@chu-grenoble.fr

**Manufacturer**	**SUNRISE SA**<br>**Laurent et Pierre MARTINOT**<br>Chaussée de Marche 598/02, 5101 Namur, Belgique<br>Tel : +32 81 26 11 26<br>Mail : laurent@hellosunrise.com et pierre@hellosunrise.com
**Methodologist**	**Dr Sébastien BAILLY**<br>Clinique Universitaire de Physiologie, Pôle Thorax et Vaisseaux, CHU GRENOBLE ALPES, 38043 GRENOBLE Cedex 09 ; Université GRENOBLE ALPES ; Inserm U1042, Laboratoire HP2, GRENOBLE, France<br>Tel : +33 4 76 76 87 66<br>Mail : SBailly@chu-grenoble.fr<br><br>**Pr Matthieu ROUSTIT**<br>Centre d'Investigation Clinique – Inserm CIC1406, CHU GRENOBLE ALPES, 38043 GRENOBLE Cedex 09 ; Université GRENOBLE ALPES ; Inserm U1042, Laboratoire HP2, GRENOBLE, France<br>Tel : +33 4 76 76 62 36<br>Mail : MRoustit@chu-grenoble.fr
**Data Management**	**Rémi AIGUEBONNE**<br>CHU GRENOBLE ALPES, Pôle Thorax et Vaisseaux,<br>38043 GRENOBLE Cedex 09, France<br>Data Manager – Base de données MARS<br>Pôle Thorax & Vaisseaux – Laboratoire d'EFCR<br>Tel : +33 4 76 76 84 81<br>Mail : remi.aiguebonne@univ-grenoble-alpes.fr
**Economic Evaluation Methodology**	**Nathalie PREAUBERT-HAYES**<br>Health Economist<br>Medconsult<br>21 Quai Alphonse le Gallo<br>92100 Boulogne-Billancourt, France<br>Tel : +33 6 89 10 86 66<br>Mail : nathalie.preaubert@medconsult.fr

TABLE OF CONTENTS

1 STUDY SYNOPSIS 14

2 SCIENTIFIC RATIONALE AND GENERAL DESCRIPTION OF THE STUDY 24

2.1 CURRENT KNOWLEDGE ABOUT THE DISEASE 24
2.2 CURRENT KNOWLEDGE ABOUT THE REFERENCE DEVICE 24
2.3 CURRENT KNOWLEDGE ABOUT THE STUDY DEVICE 25
2.4 RESEARCH HYPOTHESIS AND EXPECTED OUTCOMES 30

3 MEDICAL DEVICE UNDER INVESTIGATION 30

3.1 DESCRIPTION OF THE SUNRISE MEDICAL DEVICE 30
3.2 INTENDED USE AND TARGET POPULATION 32
3.3 MANUFACTURER 32
3.4 DEVICE IDENTIFICATION AND TRACEABILITY 32
3.5 SUPPLY OF THE MEDICAL DEVICE 33
3.6 DEVICE PACKAGING 33
3.7 DEVICE LABELING 33
3.8 SHIPPING AND STORAGE OF DEVICES 34
3.9 DISPENSING OF DEVICES 34
3.10 RETURN OF DEVICES 34
3.11 ACCOUNTABILITY 35
3.12 TRAINING PRIOR TO DEVICE USE 35

4 STUDY OBJECTIVES 35

4.1 PRINCIPAL OBJECTIVES 35
4.2 SECONDARY OBJECTIVES 36

5 STUDY ENDPOINTS 36

5.1 PRIMARY ENDPOINTS 36
5.2 SECONDARY ENDPOINTS 37

6 DESIGN OF THE CLINICAL INVESTIGATION 37

6.1 CHOSEN METHODOLOGY 37
6.2 RANDOMISATION METHOD 38
6.3 METHODS FOR ECONOMIC EVALUATION 38
6.3.1 Measurement of Efficiency 38
Type of Analysis 38
Perspective 38
Identification and Valuation of Healthcare Costs for Patients in Both Groups 38
6.3.2 Budgetary and Organizational Impact Analysis 39
6.4 MEASURES TAKEN TO REDUCE AND AVOID BIAS 40

7 IDENTIFICATION OF PARTICIPANTS 40

7.1 INCLUSION CRITERIA 40
7.2 NON-INCLUSION CRITERIA 41
7.3 END-OF-STUDY CRITERIA AND EARLY WITHDRAWAL OF PATIENTS 41
7.4 REMPLACEMENT OF PATIENTS 42
7.5 CONSTRAINTS RELATED TO THE CLINICAL INVESTIGATION 42

8 CONDUCT OF THE CLINICAL INVESTIGATION 42

8.1 STUDY DESIGN 42
8.2 PROVISIONAL SCHEDULE OF THE CLINICAL INVESTIGATION 43
8.3 IDENTIFICATION AND SÉLECTION OF PATIENTS 43
8.4 CONDUCT OF VISITS 44
8.4.1 Inclusion visit 44
8.4.2 Diagnostic procedure 46
8.4.3 Diagnosis announcement consultation/teleconsultation 48
8.4.4 Remote treatment initiation (if indicated) 49
8.4.5 Follow-up visits 49
8.5 SUMMARY OF PROCEDURES 51

9 STATISTICS 53

9.1 CALCULATION OF THE REQUIRED NUMBER OF SUBJECTS 53
9.2 STATISTICAL METHODS USED 54

10 BENEFIT / RISK RATIO 57

10.1 EXPECTED BENEFITS 57
10.2 RISKS RELATED TO THE CLINICAL INVESTIGATION 57
10.3 BENEFIT / RISK BALANCE 58
10.4 STUDY LIMITATIONS 58
10.5 RISK CONTROL AND MITIGATION 58

11 VIGILANCE AND SAFETY 59

11.1 DÉFINITIONS 59
11.2 INVESTIGATOR RESPONSIBILITIES 60
11.2.1 Evaluation of the causal relationship 61
11.2.2 Evaluation of the severity of the event 62
11.2.3 Evaluation of the progression of the event 62
11.3 METHODS OF REPORTING BY THE INVESTIGATOR TO THE SPONSOR 63
11.4 REPORTING PROCEDURES OF THE SPONSOR/MANUFACTURER TO THE COMPETENT AUTHORITIES 63
11.5 EXPECTED ADVERSE EVENTS OR EFFECTS 63

12 DATA MANAGEMENT 63

12.1 DATA COLLECTION AND PROTECTION 63
12.2 DEFINITION OF SOURCE DATA 64
12.3 DATA MANAGEMENT 65
12.4 DATA REVIEW 65
12.5 DATABASE LOCK 65

13 CONFIDENTIALITY AND PSEUDONYMIZATION OF PERSONAL DATA 66

14 **SECURITY OF SUNRISE DEVICE DATA 66**

15 CONFIDENTIALITY 67

16 CONTROL AND QUALITY ASSURANCE 67

16.1 INSTRUCTIONS FOR DATA COLLECTION 67
16.2 QUALITY CONTROL/MONITORING OF DATA 67
16.3 AUDIT AND INSPECTION 68

17 RULES FOR STOPPING THE CLINICAL INVESTIGATION 69

18 ETHICAL AND REGULATORY CONSIDERATIONS 70

19 PROTOCOL DEVIATIONS 70

20 AMENDMENT TO THE PROTOCOL 71

21 INSURANCE 71

22 PRESERVATION OF DOCUMENTS AND RESEARCH-RELATED DATA 71

23 STEERING COMMITTEE 72

24 RULES RELATING TO PUBLICATION 72

24.1 FINAL STUDY REPORT 72
24.2 SCIENTIFIC COMMUNICATIONS 73
24.3 COMMUNICATION OF RESULTS TO PATIENTS 73

25 PROCEDURE FOR OBTAINING INFORMED CONSENT 73

26 AGREEMENT AND FUNDING 74

27 BIBLIOGRAPHY 75

SIGNATURE PAGE OF THE CLINICAL INVESTIGATION PLAN

Validation of an integrated digital solution (Sunrise device) for the automated analysis of mandibular jaw movements using artificial intelligence versus polysomnography for the diagnosis of obstructive sleep apnea: a national multicenter randomized controlled trial

SUNSAS Study

This clinical investigation protocol PRO-545 version 1.7 dated June 13, 2022 has been read, understood, and approved.

**Coordonnating investigator :** Pr Jean-Louis PEPIN<br>Pôle Thorax et Vaisseaux<br>Laboratoire EFCR et Sommeil<br>CHU GRENOBLE ALPES	Date and Signature:
**Sponsor** : SUNRISE SA<br>Represented by:<br>Mr Pierre MARTINOT<br>Administrator	Date and Signature:
**Methodology**: Pr Matthieu Roustit<br>Clinical Investigation Center<br>CHU Grenoble Alpes	Date and Signature:

SIGNATURE PAGE OF THE CLINICAL INVESTIGATION PROTOCOL – PRINCIPAL INVESTIGATOR

Validation of an integrated digital solution (Sunrise device) for the automated analysis of mandibular jaw movements using artificial intelligence versus polysomnography for the diagnosis of obstructive sleep apnea: a national multicenter randomized controlled trial

SUNSAS Study

Clinical Investigation Protocol PRO-545 version 1.7 dated June 13, 2022

I have read all pages of the clinical investigation protocol for which SUNRISE SA is the sponsor. I undertake to conduct the research in accordance with the protocol and the terms and conditions defined therein, as well as any subsequent amendments provided to me by the sponsor. I commit to conducting this protocol in compliance with the principles of the “Declaration of Helsinki,” Good Clinical Practice, the Public Health Law of August 9, 2004, and the implementing decree of November 16, 2016, in particular by providing information and obtaining the patients’ written consent prior to any protocol-related procedure.

I also undertake to ensure that co-investigators and other qualified members of my team have access to copies of this protocol and related study documents to enable them to work in compliance with the provisions contained in those documents.

I am informed that my personal data will be subject to automated processing for the purposes of implementing and conducting the study. This information may potentially be transferred outside the European Union. In accordance with the amended law of January 6, 1978, I have the right to access and correct my personal information through the sponsor.

**Principal Investigator:**	**Institution:**	**Date:**	**Signature:**

1 STUDY SYNOPSIS

Study Title	Validation of an integrated digital solution (Sunrise device) for the automated analysis of mandibular jaw movements using artificial intelligence versus polysomnography for the diagnosis of obstructive sleep apnea: ā national multicenter randomized controlled trial
Short Title	SUNSAS
Coordonnating Investigator	**Pr. Jean Louis Pépin**<br>PU-PH en physiologie clinique<br>CHU de Grenoble – Université Grenoble Alpes
Sponsor	SUNRISE SA<br>Chaussée de Marche 598/02,<br>5101 Namur, Belgique
Study Rationale	In France, it is estimated that approximately 12 million people aged 30 to 69 are affected by moderate to severe obstructive sleep apnea-hypopnea syndrome (OSAHS) (AHI ≥ 15) (1).<br>Due to its prevalence and its link with obesity and chronic cardiometabolic diseases (2), OSAHS represents a genuine public health issue. OSAHS primarily causes debilitating symptoms for patients, such as daytime sleepiness (caused by sleep fragmentation), fatigue, irritability, and nocturnal snoring, which may be accompanied by sensations of choking or suffocation during sleep, affecting the quality of life of bed partners or roommates. Non-restorative sleep is also associated with concentration difficulties, nocturia (more than one urination per night), libido disorders, and attention deficits (3). OSAHS therefore leads to a significant deterioration in quality of life and may also be responsible for road (4) and workplace (5) accidents. OSAHS is also associated with long-term consequences: cardiovascular morbidity and mortality (hypertension, heart failure, atrial fibrillation, etc.), metabolic disorders (diabetes, obesity), and cognitive decline. It is described as an independent predictive factor of mortality (6,7). Its cost was estimated by McKinsey and Company in the United States to be equivalent to that of hypertension or stroke (8):

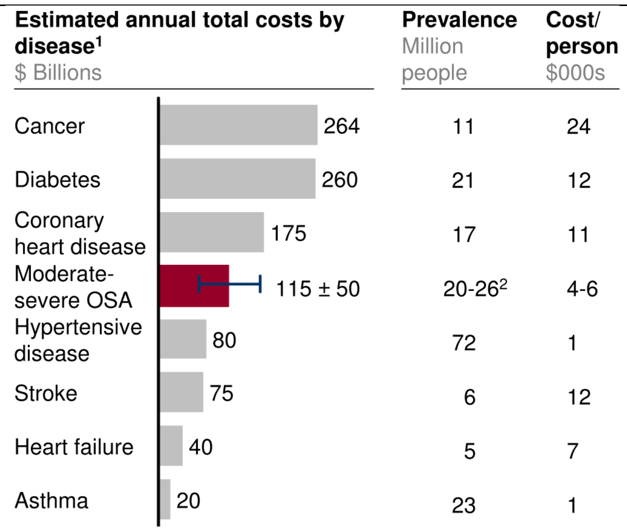

Moderate to severe OSAHS has a significant annual economic impact, comparable to that of major chronic diseases.

According to the French National Authority for Health (HAS), polysomnography conducted in a sleep laboratory is considered the gold standard for diagnosing OSAHS (9). This diagnostic test is complex to implement and requires specialized personnel for data interpretation (with potential inter-center variability in diagnostic quality) (10–12). In France, waiting times for access to polysomnography range from several weeks to several months (10), leading to delays and unequal access to care for a condition that otherwise has highly effective symptomatic treatments. Yet, the HAS recommends "rapid recording for patients suspected of OSAHS who present with severe daytime sleepiness and/or severe cardiovascular or respiratory comorbidities and/or a high-risk occupation" (7). Furthermore, the implementation of polysomnography is long and cumbersome (13). When polysomnography is indicated, sensor placement must be performed either in the sleep lab or at the physician's office. The patient must then either be hospitalized overnight or return home equipped with the sensors. This complex and highly specialized diagnostic pathway is not well suited to the prevalence and consequences of the disease (1).

That is why there is now strong expert consensus on the need to develop simplified diagnostic approaches that incorporate new technologies—including artificial intelligence—to provide robust outpatient alternatives for evaluating sleep structure and respiratory abnormalities (14,15). The benefits of diagnosing OSAHS in an outpatient setting are well established (10), but to date, no device can perform as effectively as the reference exam, polysomnography (14). In fact, polygraphy, which does not measure total

	sleep time, may underestimate the severity of OSAHS and is limited in differentiating between central and obstructive respiratory events (10,16).<br><br>The new integrated digital solution, Sunrise, enables the diagnosis of OSAHS through an original mandibular sensor that analyzes mandibular movements. This approach has been scientifically validated for identifying respiratory events during sleep (apneas, central or obstructive hypopneas) (17–21). The diagnostic solution also allows identification of sleep fragmentation and estimation of total sleep time. Interpretation is enhanced by an artificial intelligence system that standardizes the scoring quality of respiratory events. In a large single-center validation study of 376 patients, its diagnostic performance was found to be equivalent to that of polysomnography (22). The digital solution, which includes a mobile app to guide patients, enables outpatient diagnosis and easy multi-night recordings. Its integration into clinical practice could address the challenges currently faced in France and significantly transform the diagnostic approach for suspected OSAHS patients, thereby accelerating their access to care.<br><br>The study aims to determine the role of the Sunrise digital diagnostic solution in the care pathway for patients suspected of having OSAHS.<br>We hypothesize that Sunrise is both non-inferior to standard care in terms of daytime sleepiness at 3 months post-diagnosis and superior to polysomnography (whether ambulatory or in-lab) in terms of diagnostic disclosure time and treatment initiation. By accelerating treatment initiation, we further hypothesize that Sunrise is superior to standard care in improving sleepiness, quality of life, and work productivity at 3 months post-randomization. Finally, we hypothesize that the solution is non-inferior in terms of compliance with continuous positive airway pressure (CPAP) therapy.<br><br>All these care-related objectives would also demonstrate that Sunrise is a cost-effective solution compared to polysomnography, provided it reduces diagnostic and treatment-related costs while improving patients' quality of life without reducing treatment efficacy or compliance.<br><br>This ambitious study (19 centers, >800 patients), randomized, will cover the entire French territory, with half the centers in academic settings and half in private practice. The reference arm will reflect the centers' usual practice, whether ambulatory PSG or in-lab PSG. This will ensure good generalizability of the results and a high-quality medico-economic evaluation.
Technology Description	Sunrise is an integrated digital medicine solution for the diagnosis of obstructive sleep apnea-hypopnea syndrome (OSAHS), capable of detecting

	respiratory disorders through the analysis of mandibular movements, optimized by a machine learning–based artificial intelligence algorithm. It consists of a unique, innovative sensor worn on the chin during the night, connected via Bluetooth to a mobile application that guides patients through the setup process and enables the transfer of data collected by the sensor to a certified health data host, ensuring security and confidentiality of medical data.<br><br>The analyzed data is compiled and automatically transferred into a detailed report, made available to physicians via the mobile application the day after the test.
Principal Objectives	Several primary objectives will be assessed hierarchically:<br>1. a) To demonstrate the non-inferiority of Sunrise over the reference method (PSG) on daytime sleepiness at 3 months after the diagnostic consultation.<br>AND<br>b) To demonstrate the superiority of Sunrise over the reference method (PSG) on the time between the inclusion (randomization) and the diagnostic consultation.<br><br>2. To demonstrate the superiority of Sunrise over the reference method (PSG) on the time between the inclusion (randomization) and the treatment dispensation, specifically for patients who are prescribed treatment (positive airway pressure (PAP) or oral appliance).<br><br>3. To demonstrate the superiority of Sunrise over the reference method (PSG) on daytime sleepiness at 3 months post-inclusion (randomization).
Secondary Objectives	1. To demonstrate the superiority of Sunrise over the reference method (PSG) on the quality of life at 3 months post-inclusion (randomization).<br><br>2. To demonstrate the superiority of Sunrise over the reference method (PSG) on work productivity at 3 months post-inclusion (randomization).<br><br>3. To assess the economic impact of Sunrise, through a cost-effectiveness analysis expressed in cost per quality-adjusted life-year (QALY) gained at 3 months after the diagnostic consultation, compared to the reference method (PSG), from the payer's perspective.<br><br>4. To estimate the organizational and budgetary impact of deploying Sunrise in the French healthcare system over 3 years for the French health insurance (*only if the cost-effectiveness analysis is in favor of Sunrise*).

	5. To demonstrate the non-inferiority of Sunrise over the reference method (PSG) on treatment adherence, specifically for patients who are treated by PAP, at 3 months after having started the treatment.<br><br>6. To confirm the diagnostic accuracy of Sunrise compared to the reference method (PSG) in the PSG arm.<br><br>7. To measure the within-subject inter-night variability of the OSA severity index with Sunrise.
Primary Endpoints	1. a) Variation in ESS score from baseline to 3 months post-diagnosis.<br>AND<br>b) Time (in days) between inclusion (randomization) and diagnostic consultation.<br><br>2. Time (in days) between inclusion (randomization) and treatment dispensation of PAP or MAD.<br><br>3. Variation in ESS score from baseline to 3 months post-inclusion (randomization).
Secondary Endpoints	1. Variation in SF-36 and QSQ scores from baseline to 3 months post-inclusion (randomization).<br><br>2. Variation in WPAI:SAS score from baseline to 3 months post-inclusion (randomization).<br><br>3. Incremental cost-effectiveness ratio (ICER) of Sunrise compared to the reference method (PSG), expressed as the incremental cost (in €) per QALY gained 3 months post-diagnosis.<br><br>4. Net benefit (in €) for the public health insurance from the dissemination of Sunrise in the French healthcare system over 3 years.<br><br>5. Mean adherence to PAP over 30 days evaluated between D60 and D90 after PAP treatment initiation.<br><br>6. Diagnostic accuracy: sensitivity and specificity of Sunrise for detecting OSA at the recommended thresholds of 5 and 15 respiratory events per hour (for patients included in the PSG arm only).<br><br>7. Inter-night variability of the number of respiratory events per hour measured with Sunrise on multiple nights at home (for patients included in the Sunrise arm only).
Study Design	This is a prospective, controlled, randomized, parallel-arm, open-label, multicenter, national study.

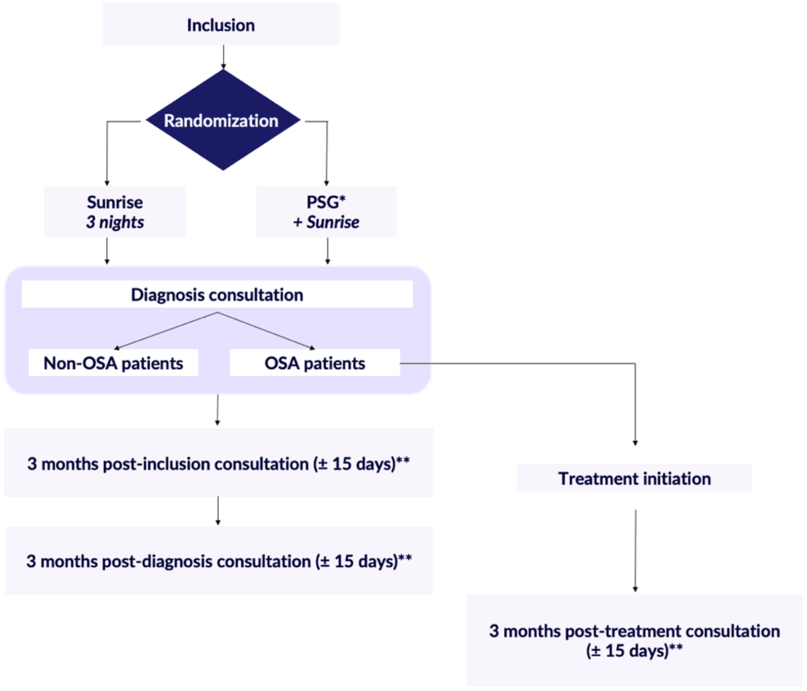

Flowchart of the clinical trial design

Patient Selection

- Selection of patients according to inclusion / non-inclusion criteria;
- Verbal explanation of the study and provision of a written information sheet;
- Answers to patient questions; patients are given a 48-hour reflection period to confirm their participation in the clinical investigation.

Inclusion Visit

- Patient inclusion after obtaining informed consent;
- Full collection of medical history (cardiovascular, cerebrovascular, metabolic, or hepatic diseases) and current treatments;
- A clinical examination will be performed;
- Completion of questionnaires: ESS, SF36, QSQ, WPAI:SAS, EQ5D-5L. Questionnaires may be completed directly by patients on a designated digital platform. The data will then be automatically transferred to the eCRF;
- Provision of the patient diary to record healthcare consumption and explanation of the importance of data collection;
- Patients will then be randomized into two groups:
  - **Experimental arm (Sunrise):** diagnostic assessment using the Sunrise solution
  - **Reference arm (PSG):** standard diagnostic procedure using the reference method—polysomnography (PSG) in a sleep lab or ambulatory PSG depending on the center's practice and patient profile

	**If Randomized to the Experimental Arm (Sunrise):**<br>• Provision of 3 Sunrise diagnostic kits and usage instructions;<br>• Scheduling of the diagnostic result consultation/teleconsultation;<br>• Scheduling of the 3-month post-inclusion consultation/teleconsultation.<br><br>**If Randomized to the Reference Arm (PSG):**<br>• Scheduling the PSG date either ambulatory or in a sleep lab according to the center's usual practice (not via the expedited clinical trial pathway);<br>• Scheduling of the diagnostic result consultation/teleconsultation according to center practices (may not be scheduled at inclusion);<br>• Scheduling of the 3-month post-inclusion consultation/teleconsultation.<br><br>**Day of Diagnostic Procedure**<br>Adverse events will be recorded after the procedure. Patients in the Sunrise arm are encouraged to proactively contact the investigator or a team member to report any adverse events if applicable.<br><br>In the Sunrise arm:<br>The patient uses the Sunrise diagnostic solution as indicated, preferably for 3 consecutive nights (within a maximum of 7 days).<br><br>In the PSG arm:<br>The patient is equipped for PSG (either in-lab or ambulatory) by a technician and also wears the Sunrise device during the same night as the PSG recording.<br><br>**Diagnostic Announcement Consultation / Teleconsultation**<br>In both arms:<br>• Communication of the diagnosis;<br>• Discussion of treatment options (if applicable);<br>• Prescription of treatment;<br>• Scheduling of the 3-month follow-up visit post-diagnosis consultation/teleconsultation;<br>• Review of the patient diary (record of healthcare consumption) and collection of any missing data;<br>• Review of current treatments;<br>• Collection of adverse events.

	**Day of Treatment Initiation (if indicated)**<br>In both arms:<br><br>First day of treatment: CPAP (Continuous Positive Airway Pressure) or Mandibular Advancement Device (MAD).<br><br>**FOLLOW-UP VISITS:**<br><br>**3 Months Post-Inclusion (± 15 days)**<br>In both arms:<br>• Completion of ESS, SF36, QSQ, EQ5D-5L, WPAI:SAS questionnaires;<br>• Review of the patient diary and collection of any missing data;<br>• Review of current treatments;<br>• Collection of adverse events.<br><br>**3 Months Post-Diagnosis Consultation (± 15 days)**<br>In both arms:<br>• Completion of ESS, EQ-5D-5L questionnaires;<br>• End of follow-up for untreated patients (not diagnosed with OSAHS);<br>• Final review and verification/correction of patient diary and healthcare consumption data;<br>• Review of current treatments;<br>• Collection of adverse events.<br><br>**3 Months Post-Treatment Initiation (± 15 days)** ***(only for treated patients)***<br>• Collection of compliance data for patients on CPAP (via telemonitoring);<br>• Measurement of sleepiness using ESS;<br>• Review of current treatments;<br>• Collection of adverse events.<br><br>Depending on inclusion, diagnosis, and treatment dates (if applicable), follow-up visits may be combined if the theoretical visit windows overlap.<br><br>Questionnaires completed electronically by patients can be submitted between 10 days before and 10 days after the scheduled follow-up visit.<br><br>Follow-up visits may be conducted in person or via teleconsultation, provided that either the 3-month post-diagnosis or 3-month post-treatment visit is in-person to collect the patient diary.
Randomization Method	Centralized electronic randomization, with random block sizes and stratification by center and by patients' ESS score at the time of inclusion:

	ESS score ≤ 12 and ESS score > 12.
Study Duration	Inclusion period: approximately 5 patients/month/center, i.e., around 22 months<br>*Feasibility of inclusion: on average, a sleep laboratory records 30 patients per week, 50% of whom are diagnosed with OSAHS*<br>*Follow-up duration: maximum of 18 months (maximum follow-up duration for patients in the PSG group, since the diagnostic delay is not known)*<br>Total study duration: approximately 40 months
Study Population	Adult patients referred for an initial diagnosis following a suspicion of OSAHS
Number of Sites	19 centers in France (public and private)
Number of patients	848 patients
Inclusion criteria	- Man or woman aged 18 to 80 years.<br>- Patient referred for suspected OSA syndrome.<br>- Patient with a smartphone and home internet connection, and capable of using a smartphone application.<br>- Patient affiliated with or benefiting from a social security system.
Non-inclusion criteria	- Patient who has already undergone a sleep recording test of any kind in the last five years prior to inclusion.<br>- Patient already treated for OSA in the last five years prior to inclusion.<br>- Patient with severe chronic obstructive or restrictive pulmonary disease, with or without oxygen (according to the judgment of the principal investigator (PI)).<br>- Patient refusing to shave their beard (if too abundant) to wear the Sunrise device.<br>- Patient with unstable cardiovascular disease or severe heart failure requiring hospitalization within the 3 months prior to inclusion or meeting the criteria of the New York Heart Association (NYHA), Class III or IV disease.<br>- Persons covered by articles L1121-5 to L1121-8 of the French Public Health Code (pregnant women, women in labor, breastfeeding mothers, individuals deprived of liberty by judicial or administrative decision, individuals under legal protection, cannot be included in clinical trials).<br>- Patient currently in the exclusion period of another study or participating in an ongoing interventional study.<br>- Patient who, in the investigator's opinion, may not be cooperative or compliant with the obligations inherent in study participation.<br>- Patient suffering from conditions affecting the rotation of the condyle in the temporomandibular joint.<br>- Patient given diagnostic priority: high-risk occupations (e.g., truck drivers, night workers, etc.).

Sample size and statistical methods	Several primary endpoints will be tested sequentially, following a predefined hierarchy established before the start of the protocol. To avoid inflation of the alpha risk due to multiple testing, the next level in the hierarchy will only be tested if the null hypothesis of the preceding level is rejected. For each level in the hierarchy, an overall alpha risk of 0.05 will be maintained.<br><br>The sample size is calculated based on the first level of this hierarchy (sleepiness scale), as no preliminary data are available for the time between inclusion and diagnosis disclosure. Assuming the smallest clinically meaningful difference is approximately 2.5 points (2 points in the most conservative estimates)[1 2], we set a non-inferiority margin of 1 point for the delta M3 – baseline difference between groups. Assuming a standard deviation of this difference of 4.6[1 3], with an alpha risk of 0.025 and a power of 85%, 381 subjects per group are required to demonstrate the non-inferiority of the intervention compared to standard care, using a one-sided t-test (PASS v15, NCSS, LLC. Kaysville, Utah, USA). Accounting for an anticipated 10% loss to follow-up, we plan to enroll 848 patients in the trial.<br><br>This sample size also enables detection of an effect size, expressed as Cohen's d, of 0.23 or 0.26, with a power of 90% and an alpha risk of 0.05 or 0.025, respectively, using a two-sided t-test, for other primary endpoints in the hierarchy. Thus, the required sample size for hierarchical endpoint 1A* also provides sufficient power (90%) to detect a small to moderate difference for endpoint 1B and subsequent levels*.<br><br>*Criterion 1A: Non-inferiority on ESS (Epworth Sleepiness Scale)<br>Criterion 1B: Superiority on diagnostic delay<br>Criterion 2: Superiority on time to treatment initiation<br>Criterion 3: Superiority on ESS at 3 months post-randomization<br><br>1 Crook S, Sievi NA, Bloch KE, Stradling JR, Frei A, Puhan MA, et al. Minimum important difference of the Epworth Sleepiness Scale in obstructive sleep apnoea: estimation from three randomised controlled trials. Thorax. 2019;74(4):390-6.<br>2. Patel S, Kon S, Nolan CM, Barker RE, Simonds AK, Morrell MJ, Man WDC. The Epworth Sleepiness Scale: Minimum Clinically Important Difference in Obstructive Sleep Apnea. Am J Respir Crit Care Med. 2018 Apr 1;197(7):961-963.<br>3. Ballester E, Badia JR, Hernández L, Carrasco E, de Pablo J, Fornas C, et al. Evidence of the effectiveness of continuous positive airway pressure in the treatment of sleep apnea/hypopnea syndrome. Am J Respir Crit Care Med. févr 1999;159(2):495-501

2 SCIENTIFIC RATIONALE AND GENERAL DESCRIPTION OF THE STUDY

2.1 CURRENT KNOWLEDGE ABOUT THE DISEASE

In France, it is estimated that approximately 12 million people aged 30 to 69 are affected by moderate to severe obstructive sleep apnea-hypopnea syndrome (OSAHS) (AHI ≥ 15), and 24 million have an AHI ≥ 5 (1).

Yet, OSAHS is frequently underdiagnosed. It is estimated that more than 85% of patients with clinically significant OSAHS do not receive an appropriate diagnosis.

Due to its prevalence and its association with obesity and chronic cardiometabolic diseases (2), OSAHS represents a true public health issue. It causes debilitating symptoms for patients such as daytime sleepiness (caused by sleep fragmentation), fatigue, irritability, and nocturnal snoring, sometimes accompanied by sensations of choking or suffocation during sleep, which negatively impact not only the patient's quality of life but also that of their partners. Non-restorative sleep is also associated with concentration difficulties, nocturia (more than one urination per night), libido disorders, and impaired alertness, among others (3).

OSAHS therefore leads to a significant and sometimes severely disabling decline in quality of life, and may contribute to road accidents (4) and workplace accidents (5). It is also linked to severe long-term consequences: cardiovascular morbi-mortality (e.g. hypertension, heart failure, atrial fibrillation), metabolic disorders (diabetes, obesity), and cognitive impairment. It has been described as an independent predictive factor of mortality (6,7).

Its economic burden has been independently estimated in the United States by McKinsey & Company to be equivalent to that of hypertension or stroke (8): moderate to severe OSAHS has a significant annual economic impact when compared to other conditions.

2.2 CURRENT KNOWLEDGE ABOUT THE REFERENCE DEVICE

According to the French National Authority for Health (HAS), polysomnography conducted in a sleep laboratory is considered the reference technique for diagnosing OSAHS (9). This diagnostic test is highly complex to implement and requires specialized personnel for data interpretation (with potential inter-center variability in diagnostic quality) (10–12). Indeed, the procedures for conducting

polysomnography are lengthy and cumbersome (13). When polysomnography is indicated, the placement of various sensors must be performed in a sleep lab or a physician's office. The patient must then either be hospitalized overnight or return home equipped with the sensors.

This complex and highly specialized diagnostic pathway is not well-suited to the prevalence and consequences of the disease (1). In France, waiting times for a PSG appointment range from several weeks to several months (10), resulting in delayed and unequal access to care for a condition that otherwise benefits from highly effective symptomatic treatment. However, the HAS recommends "rapid testing for patients suspected of OSAHS who present with severe daytime sleepiness and/or severe cardiovascular or respiratory comorbidities and/or work in a high-risk profession" (7).

This is why there is now strong expert consensus on the need to develop simplified diagnostic approaches that incorporate new technologies, including artificial intelligence, to provide robust outpatient alternatives for evaluating sleep architecture and respiratory abnormalities (14,15). The benefits of diagnosing OSAHS in an outpatient setting are well established (10), but to date, no device can perform this diagnosis as effectively as the reference examination—polysomnography (14). In fact, polygraphy, which does not measure total sleep time, may lead to underestimation of OSAHS severity, and differentiating between central and obstructive respiratory events is limited (10,16).

2.3 CURRENT KNOWLEDGE ABOUT THE STUDY DEVICE

The new integrated Sunrise solution enables the diagnosis of OSAHS using an original mandibular sensor that analyzes mandibular movements. It consists of a unique, innovative sensor placed on the chin during the night, connected via Bluetooth to a mobile application that guides patients through setup and enables the transfer of data collected by the sensor to a certified health data host, ensuring the security and confidentiality of medical data (Figure 1). Additionally, the Sunrise device is based on the latest advancements in ergonomics and design to ensure ease of use, comfort, and a positive user experience..

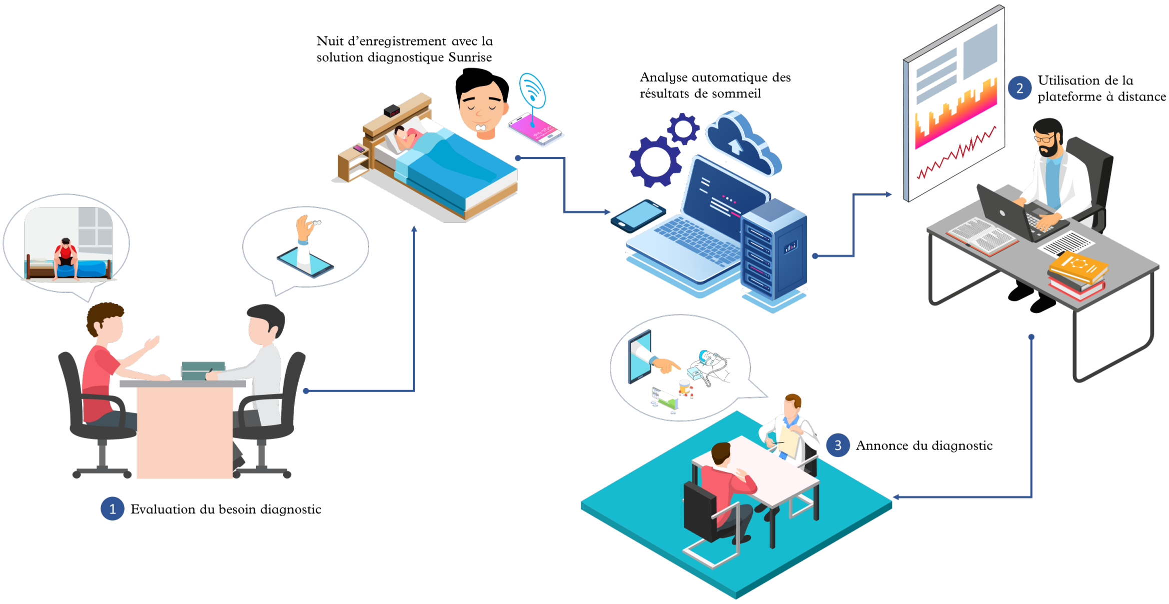

Figure 1 Mechanism of action of the integrated digital medicine solution for the diagnosis of OSAHS, Sunrise.

The integrated Sunrise digital solution enables the diagnosis of OSAHS through a novel mandibular sensor that analyzes mandibular movements. This approach has been scientifically validated for identifying respiratory events during sleep (apneas, central or obstructive hypopneas), sleep fragmentation, and total sleep time estimation (17–21). The interpretation is enhanced by an artificial intelligence system that standardizes the quality of respiratory event scoring.

The utility of mandibular movements in sleep evaluation stems from the fact that the brainstem respiratory control centers are responsible, among other functions, for maintaining oxygenation throughout the night by adjusting ventilatory drive and controlling the tone of the muscles that maintain the patency of the upper airways. These muscles are attached to the mandible, making their activity analyzable through detection of various types of mandibular movements (Figure 2). Mandibular movements allow for:

1. Accurate detection of sleep and wake phases (Figure 2, top image). A sensor that records mandibular movements can thus estimate total sleep time. A reliable measurement of total sleep time improves the accuracy of calculating the number of respiratory events per hour of sleep (better characterization of the denominator).
2. Detection of respiratory effort during sleep (Figure 2, middle and bottom images). Respiratory effort is characterized by oscillatory mandibular movements at the respiratory rate.

3. Reliable detection of micro-arousals and awakenings during sleep (number of micro-arousals and arousals per hour; Figure 2, middle and bottom images). Indeed, micro-arousals and awakenings are typically accompanied by sudden mandibular movements, often with mouth closure.

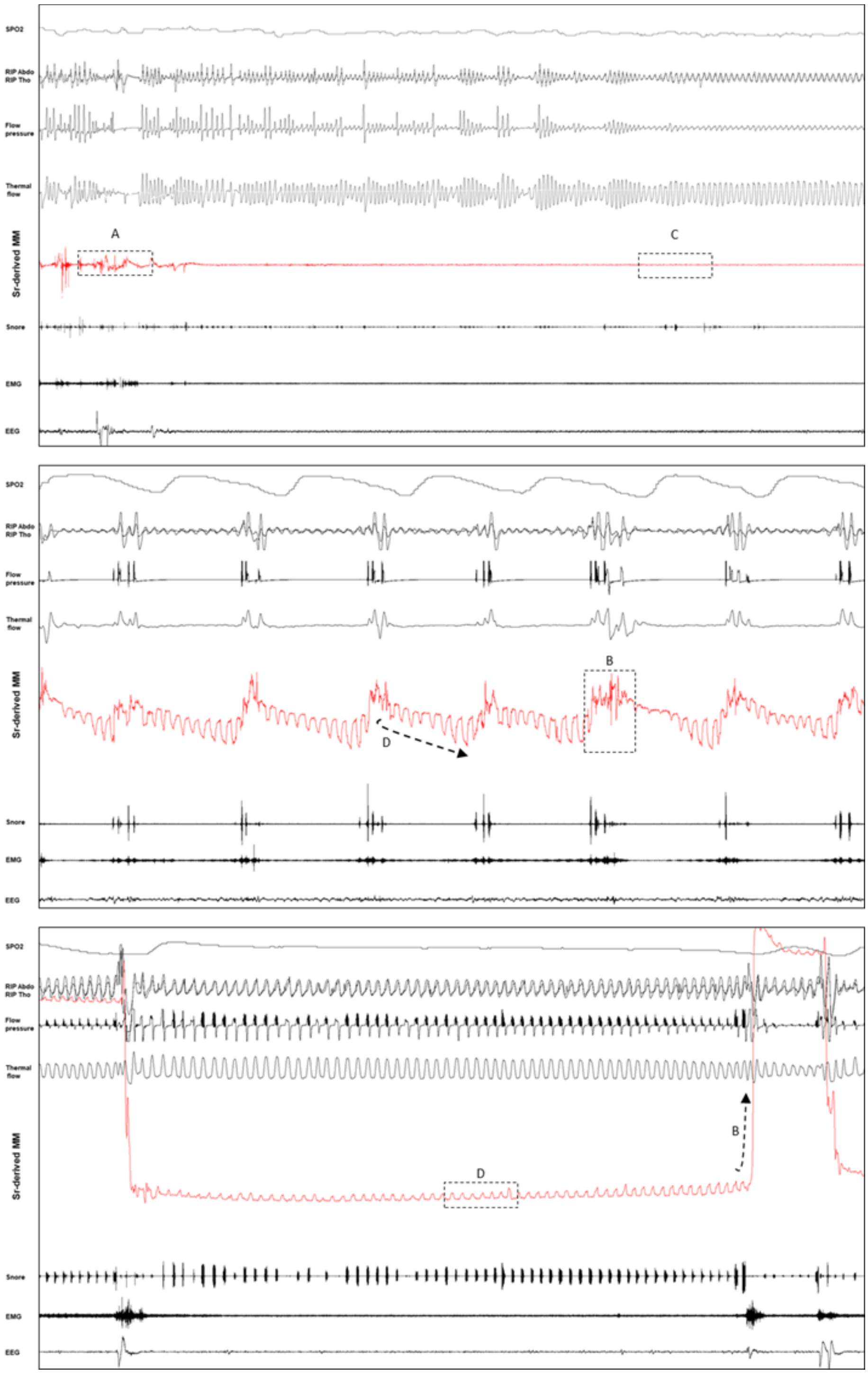

Figure 2 Behavior of the mandibular signal derived from the Sunrise sensor (in red) compared to polysomnography signals (in black), showing a transition period between wakefulness and sleep (top image). Wakefulness is characterized by rapid, irregular, and unpredictable mandibular movements (A), while quiet sleep is represented by slow, oscillatory mandibular movements at the respiratory frequency (C). The middle image illustrates a period of sleep marked by obstructive apneas. An obstructive respiratory event is defined by large-amplitude oscillatory mandibular movements indicating respiratory effort (D), ending with a sudden, rapid mouth-closing movement that marks a micro-arousal (B). The bottom image depicts another type of respiratory event, characterized by a prolonged effort period ending in a respiratory micro-arousal.

Based on these measurements (total sleep time, respiratory effort, micro-arousals, and arousals), the analysis of mandibular movements can provide an accurate measurement of the number of respiratory events occurring per hour of sleep, and thus calculate the obstructive apnea-hypopnea index (OAHI) used for the diagnosis of OSAHS (Figure 3).

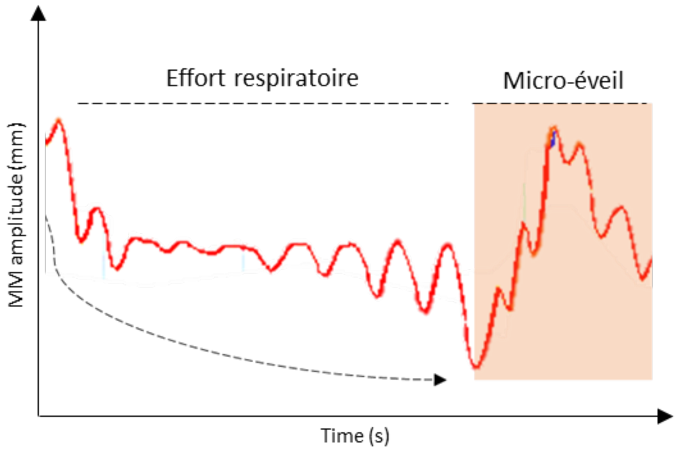

Figure 3 Representation of an obstructive respiratory event detected through mandibular movements, consisting of a period of respiratory effort and ending with a micro-arousal..

The sensor's measurements are supplemented by a questionnaire integrated into the mobile application, which characterizes the patient's clinical context and is completed by the patient before placing the sensor on the chin. The questionnaire is based on the International Classification of Sleep Disorders – Third Edition (ICSD-3) (26) and gathers information on OSAHS symptoms. The sensor data and questionnaire responses are collected through the patient's mobile app and automatically transferred at the end of the overnight recording to a cloud server in the form of encrypted data.

A machine learning–based artificial intelligence algorithm, capable of interpreting micromovements of the mandible during sleep, analyzes these data and translates them into clinically relevant parameters: obstructive apnea-hypopnea index per hour of sleep, respiratory effort, micro-arousals and arousals, sleep structure (total sleep time, sleep latency, sleep/wake stages, wake-up time), and head position. Upon completion of this analysis, all these parameters—along with the responses to the sleep questionnaire—are detailed in a report accessible on the cloud platform for the physician to support the diagnostic decision-making process.

The diagnostic performance of the Sunrise solution was recently validated in a large study conducted in 376 patients suspected of OSAHS, compared to the reference method, in-lab polysomnography (22),

showing excellent detection performance for OSAHS at clinical thresholds of 5 or 15 obstructive respiratory events per hour of sleep.

2.4 RESEARCH HYPOTHESIS AND EXPECTED OUTCOMES

This new study aims to determine the role of the Sunrise digital diagnostic solution within the care pathway of patients in France suspected of having OSAHS. Due to its ease of implementation, low cost, automation, and precision in analysis, we hypothesize that the Sunrise diagnostic solution is non-inferior to standard care in terms of sleepiness at 3 months post-diagnosis, and superior to polysomnography (whether performed at home or in a sleep lab) in terms of diagnostic disclosure delay and time to treatment initiation. By accelerating treatment initiation, we also hypothesize that Sunrise would be superior to standard care in reducing sleepiness as well as improving quality of life and work productivity at 3 months after randomization. Finally, we hypothesize that the solution is non-inferior in terms of adherence to continuous positive airway pressure (CPAP) therapy.

All of these clinically relevant objectives would also support that Sunrise is a cost-effective solution compared to polysomnography, as it could reduce costs related to the diagnostic and treatment pathway while improving patients' quality of life without reducing treatment efficacy or adherence.

If these hypotheses are confirmed, the Sunrise solution could become a simple new tool for healthcare professionals to diagnose patients suspected of OSAHS in their homes. The Sunrise solution enables outpatient diagnosis and makes it easy to repeat recordings over multiple nights (up to three). Its integration into clinical practice could address current challenges in France related to limited access and delayed diagnosis, and significantly improve the diagnostic approach and care of patients suspected of having OSAHS.

3 MEDICAL DEVICE UNDER INVESTIGATION

3.1 DESCRIPTION OF THE SUNRISE MEDICAL DEVICE

The Sunrise solution is a single-use, non-invasive medical device bearing the Class IIa CE marking under the European Directive 93/42/EEC, developed by SUNRISE SA, and designed for home use. This solution combines a miniaturized portable sensor that records mandibular movements during sleep with a digital platform that analyzes the device's data.

Sunrise is an integrated digital medicine solution for the diagnosis of OSAHS, enabling the detection of respiratory disturbances through the analysis of mandibular movements, optimized by a machine

learning–based artificial intelligence algorithm. It consists of a unique, innovative sensor placed on the chin during the night (Figure 4), connected via Bluetooth to a mobile application that guides patients through setup and enables the transfer of data to a certified health data host, ensuring data security and confidentiality.

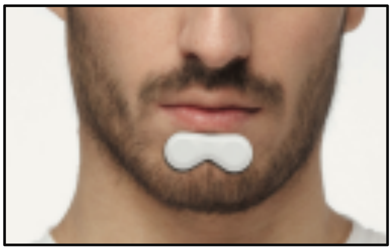

Figure 4 Sunrise medical device.

During sleep, data from the Sunrise device is transmitted via Bluetooth Low Energy (BLE) to the mobile application installed on the smartphone. The standard BLE operating range is 10 meters. To use the Sunrise solution, the user must allow the mobile app to enable the geolocation function of the phone to permit BLE connection. Geolocation data is not recorded, but it is required to allow the app to detect the nearby sensor. The analyzed data is then compiled and automatically transferred into a detailed report made available to physicians via a secure platform at the end of the night.

The Sensor

The innovative Sunrise mandibular movement sensor (Figure 4) weighs 3 grams, measures 46.5 x 20.0 x 5.6 mm, and is composed of the following three layers:

- A top protective layer made of polyurethane.
- A middle layer consisting of a flexible printed circuit board that includes:
  - An inertial measurement unit (IMU) to record movements;
  - A microprocessor with Bluetooth Low Energy module;
  - A single-use battery (3V).
- A bottom layer in contact with the skin on the patient's chin, made of biocompatible 3M™ adhesive.

The device does not contain any medication, tissues, or blood products.

The Mobile App and Digital Medicine Platform

The software components intended for users include:

- A mobile application, available on Android and iOS for the patient, which allows:

- Collection of clinical context information via a standardized questionnaire based on the International Classification of Sleep Disorders – Third Edition (ICSD-3) (26);
- Acquisition of data recorded by the sensor during the night;
- Transfer of encrypted data to the cloud server for analysis and storage.

- A cloud server interface, accessible to physicians, to view measured physiological parameters, a detailed report of sleep-related parameters, and information about the patient's clinical context. Physicians can also view raw data from the Sunrise solution.

Other software components:

- Background software modules (not accessible by the patient) that process and analyze the data.

3.2 INTENDED USE AND TARGET POPULATION

Sunrise is a support tool for detecting sleep-related breathing disorders, insomnia disorders, sleep-wake rhythm disorders, hypersomnia disorders, insufficient sleep syndrome, and bruxism in adults (over 18 years old) suspected of having sleep disorders. Sunrise also serves as a support tool for the detection of sleep-related breathing disorders in children (ages 3 to 18) suspected of having obstructive sleep apnea or habitual snoring. It is also intended to assist healthcare professionals in diagnosis and clinical decision-making, or to refer patients for further diagnostic evaluation when necessary.

3.3 MANUFACTURER

The manufacturer of the Sunrise diagnostic solution is SUNRISE SA, located at Chaussée de Marche 598/02, 5101 Namur, Belgium

3.4 DEVICE IDENTIFICATION AND TRACEABILITY

The Sunrise devices used in the SUNSAS study are identified as follows:

- Reference: SUN100
- Firmware version: 01
- Software version: 1.X.Z

The devices are identified by a unique serial number for each product, consisting of six alphanumeric characters in the format XXXYYY, where XXX is a sequence of three letters and YYY is a sequence of three digits. This serial number enables full traceability of the device by SUNRISE SA.

3.5 SUPPLY OF THE MEDICAL DEVICE

The Sunrise solution, which includes the mobile application and the Sunrise device, will be supplied by SUNRISE SA. The Sunrise application, available on standard app download platforms such as Google Play and the App Store, will enable the transmission of data to the Sunrise platform for analysis by an algorithm.

3.6 DEVICE PACKAGING

Inside the Sunrise solution box (a white rectangular box measuring 120 x 160 x 25 mm) shown in Figure 5, the following items are included:

- A single-use Sunrise device
- A quick start guide explaining how to download the app, connect the sensor and the app, and use the device
- A comprehensive user manual for the Sunrise solution
- A measuring tape for determining neck circumference
- Three additional adhesives to reinforce the sensor's attachment
- A prepaid return envelope for sending the device back to SUNRISE SA after use for reconditioning

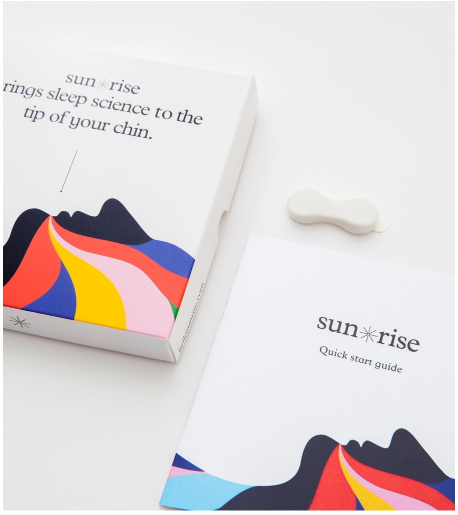

Figure 5 Conditionnement de la solution Sunrise.

3.7 DEVICE LABELING

SUNRISE SA is responsible for labeling the Sunrise devices. The labeling includes, among other things (Figure 6):

- The device reference,

- The device serial number,
- The device expiration date,
- The CE marking of the device.

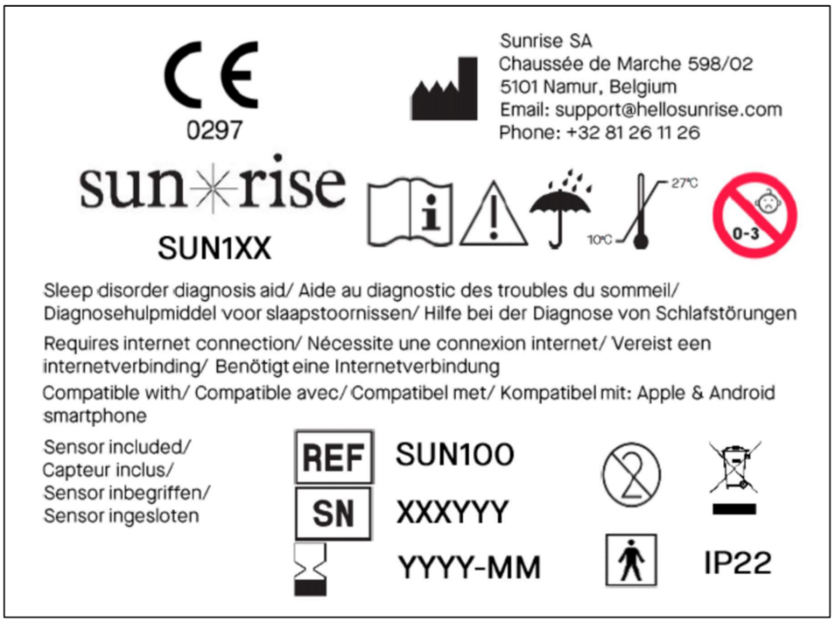

Figure 6 Labeling of the Sunrise Device.

3.8 SHIPPING AND STORAGE OF DEVICES

The Sunrise devices will be shipped by SUNRISE SA to the study's participating centers.

The devices will be stored at the respective participating centers before being distributed to patients.

The storage duration and conditions must comply with those specified by the manufacturer.

3.9 DISPENSING OF DEVICES

During the inclusion visit, each patient randomized to the experimental arm (Sunrise) will receive three devices, each accompanied by a prepaid return envelope. In the reference arm (PSG), the sensor will be dispensed on the night of the polysomnographic test.

3.10 RETURN OF DEVICES

After using the three Sunrise devices for the three nights of recording, the patient may return the devices to SUNRISE SA for recycling, using the three prepaid return envelopes provided for this purpose. The Sunrise devices used in the reference arm (PSG) will be returned to SUNRISE SA during the study by the participating centers.

At the end of the study, any unused Sunrise devices will be returned to SUNRISE SA by the participating centers.

3.11 ACCOUNTABILITY

The Sunrise solution is a CE-marked device used according to its intended purpose. Therefore, no specific accountability procedure for the devices is required for this study. The devices will be supplied to the centers in accordance with SUNRISE SA's operational procedures.

3.12 TRAINING PRIOR TO DEVICE USE

Patients randomized to the Sunrise arm will use the device according to the instructions for use (document WD-155) provided with the device.

For patients randomized to the PSG arm, the team members responsible for equipping the patients with the Sunrise device on the night of the PSG will be trained in the use of the device by SUNRISE SA.

No medical or surgical procedure is involved in the use of the Sunrise device.

4 STUDY OBJECTIVES

4.1 PRINCIPAL OBJECTIVES

Several primary objectives will be assessed in a hierarchical manner:

1. a) To demonstrate the non-inferiority of Sunrise over the reference method (PSG) on daytime sleepiness at 3 months after the diagnostic consultation.
   AND
   b) To demonstrate the superiority of Sunrise over the reference method (PSG) on the time between the inclusion (randomization) and the diagnostic consultation.

2. To demonstrate the superiority of Sunrise over the reference method (PSG) on the time between the inclusion (randomization) and the treatment dispensation, specifically for patients who are prescribed treatment (positive airway pressure (PAP) or oral appliance).

3. To demonstrate the superiority of Sunrise over the reference method (PSG) on daytime sleepiness at 3 months post-inclusion (randomization).

4.2 SECONDARY OBJECTIVES

1. To demonstrate the superiority of Sunrise over the reference method (PSG) on the quality of life at 3 months post-inclusion (randomization).

2. To demonstrate the superiority of Sunrise over the reference method (PSG) on work productivity at 3 months post-inclusion (randomization).

3. To assess the economic impact of Sunrise, through a cost-effectiveness analysis expressed in cost per quality-adjusted life-year (QALY) gained at 3 months after the diagnostic consultation, compared to the reference method (PSG), from the payer's perspective.

4. To estimate the organizational and budgetary impact of deploying Sunrise in the French healthcare system over 3 years for the French health insurance (*only if the cost-effectiveness analysis is in favor of Sunrise*).

5. To demonstrate the non-inferiority of Sunrise over the reference method (PSG) on treatment adherence, specifically for patients who are treated by PAP, at 3 months after having started the treatment.

6. To confirm the diagnostic accuracy of Sunrise compared to the reference method (PSG) in the PSG arm.

7. To measure the within-subject inter-night variability of the OSA severity index with Sunrise.

5 STUDY ENDPOINTS

5.1 PRIMARY ENDPOINTS

1. a) Variation in ESS score from baseline to 3 months post-diagnosis.
   AND

b) Time (in days) between inclusion (randomization) and diagnostic consultation.

2. Time (in days) between inclusion (randomization) and treatment dispensation of PAP or MAD.

3. Variation in ESS score from baseline to 3 months post-inclusion (randomization).

5.2 SECONDARY ENDPOINTS

1. Variation in SF-36 and QSQ scores from baseline to 3 months post-inclusion (randomization).

2. Variation in WPAI:SAS score from baseline to 3 months post-inclusion (randomization).

3. Incremental cost-effectiveness ratio (ICER) of Sunrise compared to the reference method (PSG), expressed as the incremental cost (in €) per QALY gained 3 months post-diagnosis.

4. Net benefit (in €) for the public health insurance from the dissemination of Sunrise in the French healthcare system over 3 years.

5. Mean adherence to PAP over 30 days evaluated between D60 and D90 after PAP treatment initiation.

6. Diagnostic accuracy: sensitivity and specificity of Sunrise for detecting OSA at the recommended thresholds of 5 and 15 respiratory events per hour (for patients included in the PSG arm only).

7. Inter-night variability of the number of respiratory events per hour measured with Sunrise on multiple nights at home (for patients included in the Sunrise arm only).

6 DESIGN OF THE CLINICAL INVESTIGATION

6.1 CHOSEN METHODOLOGY

- Superiority study
- Prospective

- Randomized
- Controlled
- Parallel-arm
- Open-label
- Multicenter
- Comparative between the Sunrise device and the reference method (PSG performed either ambulatory or in a sleep laboratory)

6.2 RANDOMISATION METHOD

Patient randomization into the two study arms will be electronic and centralized, using random block sizes, with stratification by study center and stratification based on the patient's ESS score at the time of inclusion: ESS score ≤ 12 and ESS score > 12..

6.3 METHODS FOR ECONOMIC EVALUATION

6.3.1 Measurement of Efficiency

Type of Analysis

In accordance with HAS recommendations, efficiency will be assessed through a cost-utility study, given the impact of OSAHS, diagnostic delay, and time to treatment initiation on patients' quality of life. The outcome measure will therefore be the QALY (Quality-Adjusted Life Year)**.** The two patient groups (Sunrise vs. PSG) will be compared 3 months after diagnosis by relating their healthcare costs to outcomes expressed in QALYs.

Perspective

The adopted perspective for this study is that of the payer, i.e., Health Insurance. This is a narrower perspective than that recommended by best methodological practices, but this choice was made for a secondary objective, since the majority of costs associated with the diagnostic and therapeutic management of OSAHS in France are direct medical costs borne by Health Insurance.

Identification and Valuation of Healthcare Costs for Patients in Both Groups

In both study groups, healthcare utilization will be identified using patient diaries designed specifically for the study. These will be completed continuously throughout the patients' participation—from

inclusion up to the 3-month visit following the diagnostic consultation/teleconsultation. During the study, these diaries will record in particular the following:

- Medical consultations, both general practitioners and specialists, conducted in private practice or in hospital
- Hospitalizations
- Medications
- Medical and paramedical procedures (physiotherapy, nursing, etc.)
- Laboratory tests, pathology, imaging
- Medical transportation

These healthcare utilizations will be valued at the current reimbursement rates, using the available official coding and pricing classifications (e.g., CCAM, NGAP, TNB, T2A, etc.).

Identification and Quantification of Effectiveness Data

Utility data will be calculated using the EQ-5D-5L questionnaire, the scoring of which has recently been validated in France. This questionnaire will be completed by the patient at three time points: at inclusion, 3 months after inclusion (randomization), and 3 months after the diagnostic consultation/teleconsultation.

Efficiency Ratio

The cost-utility ratio (CUR) between the two strategies, expressed as cost per QALY gained 3 months after the diagnostic consultation/teleconsultation, will combine in the numerator the differential in direct costs between the two strategies (C_sunrise - C_ref) and in the denominator the differential in QALYs (E_sunrise - E_ref):

$$CUR = (C\_sunrise - C\_ref) / (E\_sunrise - E\_ref)$$

This ratio informs on the health benefit obtained relative to the collective investment mobilized. The graphical interpretation and statistical analysis of the efficiency ratio are presented in Chapter 10.

6.3.2 Budgetary and Organizational Impact Analysis

If the economic analysis demonstrates the efficiency of managing OSAHS using the Sunrise device under investigation, a budget impact analysis (BIA) will be conducted. The BIA complements the

efficiency analysis by informing decision-makers about the healthcare system's financial capacity to support the deployment of a new care strategy. It helps identify the organizational changes expected in patient care pathways and the resources required to implement these new models.

We therefore propose an estimate of the budgetary impact for the French National Health Insurance (Assurance Maladie) associated with the rollout of the Sunrise strategy in the care pathway of patients suspected of having OSAHS, over a 3-year period. This estimate will be based on the costs incurred and avoided through the implementation of the Sunrise strategy. The BIA will be conducted in accordance with the 2016 HAS recommendations[1], and it will use methodological inputs identified in the efficiency study, as well as data collected during the study (e.g., time to diagnosis, clinical symptoms, complications, and overall healthcare utilization).

The difference between incurred and avoided costs will allow us to calculate the net benefit for the Health Insurance system from the nationwide implementation of this strategy in France over a 3-year time horizon.

6.4 MEASURES TAKEN TO REDUCE AND AVOID BIAS

Randomization is intended to avoid selection bias by creating, on average, two comparable groups. Furthermore, the participating centers are both public and private institutions located throughout France, thereby limiting any bias related to center selection.
For the analysis of secondary endpoint no. 6, the reading of all PSG recordings will be performed by a centralized laboratory.

7 IDENTIFICATION OF PARTICIPANTS

7.1 INCLUSION CRITERIA

A patient must meet all of the following inclusion criteria to be enrolled in the study:

- Male or female aged 18 to 80 years;
- Patient referred for suspected sleep apnea syndrome;

[1] https://www.has-sante.fr/upload/docs/application/pdf/201612/guide_methodologique__choix_methodologiques_pour_lanalyse_de_limpact_budgetaire_a_la_has_.pdf)

- Patient with a smartphone and home internet access, and able to use a smartphone application;
- Patient affiliated with or beneficiary of a social security scheme.

7.2 NON-INCLUSION CRITERIA

A patient must not meet any of the following criteria to be included in the study:

- Patient who has already undergone any type of sleep recording test within the five years preceding inclusion;
- Patient already treated for OSAHS (Obstructive Sleep Apnea-Hypopnea Syndrome) within the five years preceding inclusion;
- Patient with a severe chronic obstructive or restrictive pulmonary disease, with or without oxygen therapy (as judged by the principal investigator);
- Patient who refuses to shave their beard (if too thick) to wear the Sunrise device;
- Patient with unstable cardiovascular disease or severe heart failure requiring hospitalization within the three months preceding inclusion, or classified as Class III or IV according to the New York Heart Association;
- Individuals referred to in Articles L1121-5 to L1121-8 of the French Public Health Code (pregnant women, women in labor, breastfeeding mothers, persons deprived of liberty by judicial or administrative decision, persons under legal protection — may not be included in clinical trials);
- Patient currently excluded from another study or currently participating in an interventional study;
- Patient who, in the investigator’s opinion, is likely to be uncooperative or non-compliant with the obligations of participating in the study;
- Patient with pathologies affecting the rotation of the condyle in the temporomandibular joint;
- Patient with priority diagnostic needs due to high-risk occupations (e.g., truck drivers, night workers, etc.).

7.3 END-OF-STUDY CRITERIA AND EARLY WITHDRAWAL OF PATIENTS

The clinical investigation is considered complete for a patient once they have attended their final visit as specified in the protocol—namely, the 3-month post-diagnosis visit for non-OSAHS patients, or the 3-month post-treatment follow-up for OSAHS patients. A maximum follow-up

period of 18 months post-inclusion is planned, after which the patient will be withdrawn from the study.

The patient is free to withdraw their consent to participate in the study at any time, without having to provide a reason. The investigator may also withdraw the patient from the study at any time if they judge that the patient's health condition is not compatible with continued participation. If necessary, appropriate medical care will be offered to patients who are withdrawn from the study. At the end of the study or in the event of early termination, the study exit form will be completed in the CRF. In the case of early termination, the reason for withdrawal must be indicated:

- The patient withdrew their consent
- Investigator's decision
- Lost to follow-up
- Patient death
- Follow-up period too long (more than 18 months)
- Sponsor's decision
- Adverse event requiring the patient to stop participation in the study

A patient is considered lost to follow-up if the investigator is unable to contact them. The investigator must attempt to contact the patient at least three times using two different methods (e.g., phone, email, mail, etc.) before considering them lost to follow-up.

Unless the patient explicitly objects, the data collected before their withdrawal will be used in the context of the study.

7.4 REMPLACEMENT OF PATIENTS

Patients who withdraw from the study prematurely will not be replaced.

7.5 CONSTRAINTS RELATED TO THE CLINICAL INVESTIGATION

The patient may participate simultaneously in another non-interventional study, provided it does not interfere with the assessments carried out in this protocol. There is no exclusion period following this protocol.

8 CONDUCT OF THE CLINICAL INVESTIGATION

8.1 STUDY DESIGN

The experimental design of the clinical investigation is shown in Figure 7 below.

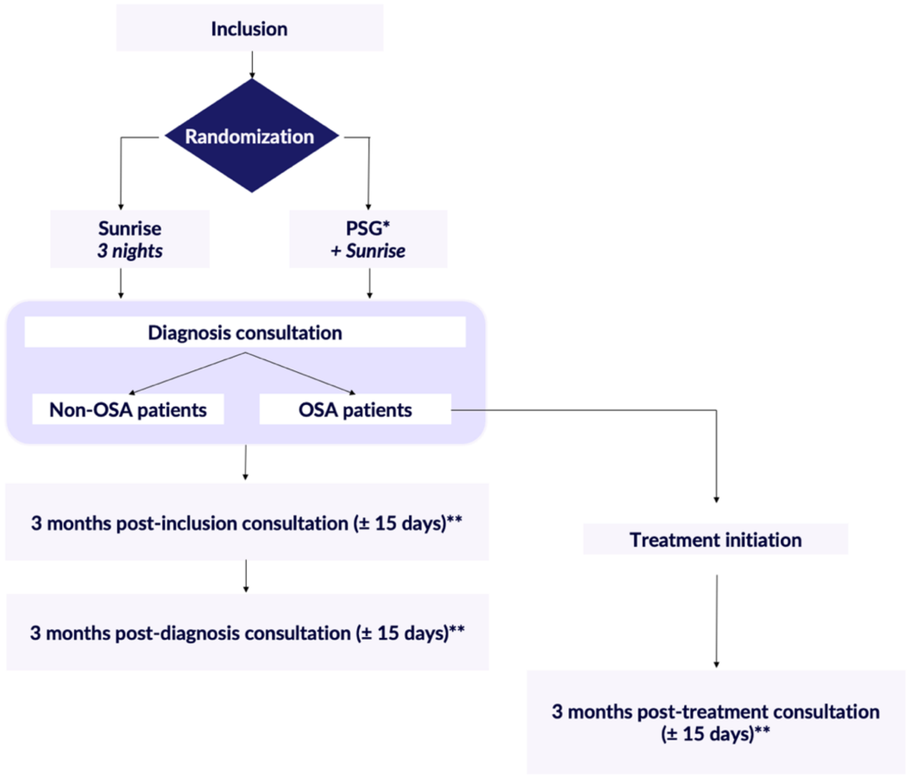

* PSG in a laboratory or outpatient setting according to standard practice (and not via the specific clinical trial pathway for participants, which could significantly reduce the waiting period).

Figure 7 :Flowchart of the clinical trial design.

8.2 PROVISIONAL SCHEDULE OF THE CLINICAL INVESTIGATION

Inclusion duration: approximately 5 patients/month/center, or about 22 months. Enrollment is competitive.

Feasibility of inclusion: on average, a sleep laboratory registers 30 patients per week, 50% of whom are diagnosed with OSAHS (Obstructive Sleep Apnea-Hypopnea Syndrome).

Follow-up duration: maximum 18 months (maximum follow-up duration for patients in the PSG group, knowing that the diagnostic delay is unknown).

Total study duration: approximately 40 months.

8.3 IDENTIFICATION AND SÉLECTION OF PATIENTS

Les patients seront sélectionnés sur base des critères d'inclusion/non-inclusion de l'étude mentionnés à la section 7 du protocole. Les patients sélectionnés recevront alors une information orale concernant l'étude et un document d'information écrit. Les patients auront la possibilité de poser toutes les questions sur l'étude à l'investigateur et disposent s'ils le souhaitent d'un délai de réflexion de 48 heures pour confirmer leur participation à l'investigation clinique.

Patients will be recruited based on scheduled appointments at the various centers participating in the clinical trial. Preselected patients will be those coming for a first diagnosis of sleep apnea following

suspicion of OSAHS, including patients presenting signs and/or symptoms and/or comorbidities commonly associated with OSAHS, such as:

- Daytime sleepiness
- Fatigue
- Nocturnal snoring
- Non-restorative sleep
- Concentration difficulties
- Observed apneas
- Sensations of choking/suffocation during sleep
- Hypertension
- Comorbidities (cardiovascular and cerebrovascular history, diabetes, etc.)

Patients will be selected based on the inclusion/non-inclusion criteria of the study mentioned in section 7 of the protocol. Selected patients will then receive oral information about the study and a written information document. Patients will have the opportunity to ask any questions about the study to the investigator and, if they wish, have a 48-hour reflection period to confirm their participation in the clinical investigation.

8.4 CONDUCT OF VISITS

8.4.1 Inclusion visit

Patients agreeing to participate in the clinical trial will only be included after obtaining informed consent. The inclusion visit will include a comprehensive collection of medical history (cardiovascular, cerebrovascular, metabolic, or hepatic diseases) and current treatments. A clinical examination will include:

- Measurement of anthropometric parameters: sex, age (years), weight (kg), height (cm), body mass index (BMI) in kg/m², neck circumference (cm), waist circumference (cm), and hip circumference (cm);
- Measurement of blood pressure (mmHg) and heart rate (bpm);
- Collection of risk factors including evaluation of smoking status, alcohol consumption, respiratory allergies;
- Collection of clinical signs for suspected sleep apnea syndrome.
-

The patient will also complete 5 questionnaires:

- Epworth Sleepiness Scale (ESS);

- Generic health-related quality of life questionnaire (EQ-5D-5L) (useful for measuring efficiency);
- Quebec Sleep Questionnaire (QSQ);
- Generic quality of life questionnaire (SF-36);
- Work Productivity and Activity Impairment Questionnaire: Sleep Apnea Syndrome (WPAI:SAS).

The questionnaires can be completed directly by patients on a dedicated digital platform. The data will then be automatically transferred into the eCRF.

Examinations and questionnaires completed routinely before consent are not to be repeated if performed within 6 weeks of the inclusion visit unless deemed necessary by the investigator.

Patients will also be given a “patient diary” to record all their healthcare consumption and use of the healthcare system (doctors, hospitalizations, medications, laboratory tests, etc.) during their participation in the study, up to 3 months post-diagnosis. The importance of this record and data completeness will be explained to the patient to estimate cost differences in management between the two patient groups evaluated during the study. The diary should be completed by the patient at each contact with a healthcare professional or by the healthcare professional themselves. The CRA will review the diary at each visit to ensure ongoing completion and identify any missing data. The completed patient diary will be returned to the investigator at the 3-month post-diagnosis visit.

The patient will be randomized into one of the two study arms. The randomization procedure will allow random allocation into the following arms:

1. **Experimental arm**: diagnostic management using the Sunrise solution.
2. **Reference arm**: usual diagnostic management by the reference method (PSG in-lab or ambulatory, depending on the routine practice of each participating center).

<u>The experimental arm (Sunrise):</u>

- Patients randomized to this arm will receive 3 diagnostic kits of the Sunrise solution and instructions for use;
- A consultation or teleconsultation to announce the diagnosis will be scheduled with the patients;
- A follow-up consultation will be scheduled 3 months after inclusion (randomization).

<u>The reference arm (PSG):</u>

- Patients randomized to this arm will receive an appointment for PSG, either ambulatory or in a sleep laboratory, according to the routine practice of the center (and not through the specific pathway for clinical trial participants, which can significantly reduce waiting times);
- A consultation or teleconsultation to announce the diagnosis will be scheduled based on the center's routine practice (this visit may not be planned at the time of the inclusion visit);
- A follow-up consultation will be scheduled 3 months after inclusion (randomization).

8.4.2 Diagnostic procedure

Adverse events will be collected following the procedure. Patients in the Sunrise arm are encouraged to proactively contact the investigator or a member of the research team to report any adverse events if they occur.

<u>In the experimental arm (Sunrise):</u>

The patient uses the Sunrise diagnostic solution as indicated for 3 nights, preferably consecutive (within a maximum period of 7 days), changing the device each night since it is a single-use device.

The patient must first download the mobile application on their smartphone. The patient has the option to return the Sunrise sensors to SUNRISE SA using the prepaid envelope provided with the Sunrise diagnostic solution.

The Sunrise device is a portable device that records mandibular movements, head movements, and head position during sleep. The Sunrise solution was developed to obtain all the necessary sleep parameters for diagnosing OSAHS using a single device placed on the chin. Its mode of action is based on mandibular movements, which allow identification of respiratory events during sleep, as validated by numerous publications (17–22).

Mandibular movements allow

- Precise detection of wake and sleep phases.
- Detection of respiratory effort during sleep. Respiratory effort is characterized by large-amplitude oscillatory mandibular movement at the breathing frequency.
- Detection of micro-arousals and arousals occurring during sleep (number of micro-arousals and arousals per hour).

From these measurements (i.e., total sleep time, respiratory efforts, micro-arousals, and arousals), analysis of mandibular movements can provide an accurate measure of the number of respiratory events occurring per hour of sleep and thus calculate the respiratory event index (REI), which includes obstructive and mixed apnea-hypopneas, as well as respiratory micro-arousals (RMA).

Once data is collected by the sensor, it is automatically analyzed at the end of the night by a machine learning algorithm capable of decoding the micromovements of the mandible during sleep. These data are translated into sleep parameters of interest:

- Sleep characterization:
  - Total sleep time
  - Time spent in Rapid Eye Movement (REM) versus non-REM stages (as a percentage of total sleep time and in minutes)
  - Time spent in each sleep position (as a percentage of total sleep time)
- Respiratory events:
  - Apnea-Hypopnea Index (AHI) (number of apneas-hypopneas per hour of sleep)
  - Respiratory Micro-Arousals (RMA) (number of respiratory micro-arousals per hour of sleep)
  - Respiratory Event Index (REI) (AHI + RMA per hour of sleep)
  - Time spent in respiratory effort (as a percentage of total sleep time)
  - REI by sleep position

The physician will diagnose based on the detailed report including analyzed data made available via a secure platform at the end of the night. Instructions for accessing patient data are provided in document WD-199.

<u>In the reference arm (PSG):</u>

The patient is equipped for PSG in the sleep laboratory or ambulatory setting by a technician. The patient is also equipped with the Sunrise diagnostic solution on the night concurrent with the PSG recording. Each center will receive on loan a smartphone with the mobile application, which will be used for patients in the experimental arm.

PSG is the reference examination used to detect sleep disorders. It combines the collection of electroencephalogram (EEG), electromyogram (EMG), and electro-oculogram (EOG) to differentiate sleep stages. Airflow quantification is done by thermistor and nasal pressure measurement. Respiratory effort is assessed using thoracic and abdominal belts. Oxygen saturation (SaO2) and electrocardiogram (ECG) measurements evaluate the impact of respiratory events on blood oxygenation and heart rate. The studied parameters are:

- Sleep characterization:
  - Total sleep time
  - Time spent in stages 1-2, 3-4, and REM sleep (as a percentage of total sleep time and in minutes)
  - Time spent in each sleep position (as a percentage of total sleep time)
- Respiratory events:
  - AHI (number of apneas-hypopneas per hour of sleep)
  - Obstructive AHI (number of obstructive apneas-hypopneas per hour of sleep)
  - Mixed AHI (number of mixed apneas-hypopneas per hour of sleep)
  - Respiratory Micro-Arousals (RMA) (number of respiratory micro-arousals per hour of sleep)
  - Respiratory Event Index (REI) (AHI + RMA per hour of sleep)
  - Mean nocturnal SaO2, minimum nocturnal SaO2, time spent below 90% SaO2 (in minutes)

The physician will diagnose based on the PSG recording reading performed by the center (routine practice).

For the analysis of secondary endpoint number 6, a centralized reading of all PSG recordings will be performed according to the guidelines recommended by the AASM (American Academy of Sleep Medicine).

8.4.3 Diagnosis announcement consultation/teleconsultation

The diagnosis announcement can be done during an in-person consultation at the hospital or via teleconsultation.

In both arms:

- Diagnosis announcement;

- Discussion of treatment options (if applicable);
- Prescription of treatment;
- A visit 3 months after the diagnosis announcement consultation/teleconsultation will be scheduled with the patients;
- Review of the patient diary (identification of healthcare utilization) and collection of missing data if applicable;
- Verification of ongoing treatments;
- Collection of adverse events.

8.4.4 Remote treatment initiation (if indicated)

In both arms:

Patient's first day of treatment: treatment with Continuous Positive Airway Pressure (CPAP) or Mandibular Advancement Device (MAD).

8.4.5 Follow-up visits

Depending on the dates of the inclusion visit, the diagnosis announcement consultation/teleconsultation, and the start of treatment (if applicable), follow-up visits may be combined if the theoretical visit windows overlap.

Questionnaires completed electronically by patients can be filled out from 10 days before up to 10 days after the scheduled follow-up visit date.

Follow-up visits can be conducted in person or via teleconsultation, provided that either the visit 3 months post-diagnosis or 3 months post-treatment is done in person to collect the patient diary.

Consultation/teleconsultation 3 months after inclusion (randomization) (± 15 days)

In both arms:

- Measurement of sleepiness using the ESS;
- Measurement of quality of life using the SF-36, EQ5D-5L, and QSQ questionnaires;
- Measurement of work productivity using the WPAI:SAS questionnaire;
- Review of the patient diary (identification of healthcare utilization) and collection of missing data if applicable;
- Verification of ongoing treatments;

- Collection of adverse events.

Consultation/teleconsultation 3 months after the diagnosis announcement consultation/teleconsultation (± 15 days)

In both arms:

- Measurement of sleepiness using the ESS;
- Measurement of quality of life using the EQ-5D-5L questionnaire for efficiency measurement;
- Discontinuation of follow-up for untreated patients (not diagnosed with SAHOS);
- Final review of the patient diary (identification/verification/corrections of healthcare utilization) and verification of data completeness;
- Verification of ongoing treatments;
- Collection of adverse events.
-

Consultation/teleconsultation 3 months after treatment initiation (± 15 days) (only for treated patients)

In both arms:

- Collection of adherence data for patients treated with CPAP (via telemonitoring);
- Measurement of sleepiness using the ESS;
- Verification of ongoing treatments;
- Collection of adverse events.

8.5 SUMMARY OF PROCEDURES

The procedures carried out as part of this clinical investigation are presented in Table 1 below..

Table 1 Procedures carried out as part of this clinical investigation.

	Inclusion visit*	Diagnostic procedure	Diagnosis disclosure consultation/ teleconsultation	Remote treatment initiation	Consultation/ teleconsultation 3 months post-inclusion	Consultation/ Teleconsultation 3 months post-diagnosis	Consultation / Teleconsultation 3 months post-treatment
Patient information	✓						
Eligibility criteria	✓						
Informed consent	✓						
Medical history	✓						
Randomization	✓						
Clinical examination	✓						
ESS questionnaire	✓				✓	✓	✓
SF-36, QSQ, WPAI:SAS questionnaires	✓				✓		
EQ5D-5L questionnaire	✓				✓	✓	
Sunrise/PSG		✓					
CPAP or MAD for OSA patients				✓			
CPAP adherence							✓
Patient logbook (record of healthcare consumption)			✓		✓	✓	

	Inclusion visit*	Diagnostic procedure	Diagnosis disclosure consultation/ teleconsultation	Remote treatment initiation	Consultation/ teleconsultation 3 months post-inclusion	Consultation/ Teleconsultation 3 months post-diagnosis	Consultation / Teleconsultation 3 months post-treatment
Current treatments	✓		✓		✓	✓	✓
Collection of adverse events	✓	✓	✓		✓	✓	✓

* Examinations performed and questionnaires completed routinely before obtaining consent should not be repeated if conducted within 6 weeks of the inclusion visit, unless the investigator deems it necessary.

9 STATISTICS

9.1 CALCULATION OF THE REQUIRED NUMBER OF SUBJECTS

The sample size is calculated based on the first level of the hierarchy (sleepiness scale, with no preliminary data available for the time between inclusion and diagnosis announcement). Considering that the smallest clinically relevant difference is approximately 2.5 points (2 points in the lowest estimates) [1,2], we set a non-inferiority margin of 1 point for the difference in delta M3 – baseline between groups. Assuming a standard deviation of this difference of 4.6[1,3], with an alpha risk of 0.025 and a power of 85%, 381 subjects per group are required to demonstrate the non-inferiority of the intervention compared to the standard, using a one-sided t-test (PASS v15, NCSS, LLC. Kaysville, Utah, USA). Anticipating a 10% dropout rate, we plan to include 848 patients in the trial.

This sample size allows detection of an effect size, expressed by Cohen's d, of 0.23 or 0.26, with a power of 90% and an alpha risk of 0.05 or 0.025, respectively, for a two-sided t-test, for the other primary endpoints in the hierarchy. Thus, the required number of subjects for criterion 1A in the hierarchy allows detection of a small to medium difference with sufficient power (90%) for criterion 1B and subsequent levels.

We do not have preliminary data regarding the mean and standard deviation of the delay until diagnosis or treatment initiation, which are heterogeneous between centers and between outpatient PSG and sleep laboratory PSG. For this reason, we expressed the expected effect size using Cohen's d, with a power of 90%, based on the sample size calculated for criterion 1A. Regarding the ESS, considering the common standard deviation above, this effect size corresponds to a difference of 1.2 points between groups.

*Criterion 1A: Non-inferiority of ESS; Criterion 1B: Superiority of diagnostic delay; Criterion 2: Superiority of treatment initiation delay; Criterion 3: Superiority of ESS 3 months post-randomization.

1 Crook S, Sievi NA, Bloch KE, Stradling JR, Frei A, Puhan MA, et al. Minimum important difference of the Epworth Sleepiness Scale in obstructive sleep apnoea: estimation from three randomised controlled trials. Thorax. 2019;74(4):390-6.
2. Patel S, Kon S, Nolan CM, Barker RE, Simonds AK, Morrell MJ, Man WDC. The Epworth Sleepiness Scale: Minimum Clinically Important Difference in Obstructive Sleep Apnea. Am J Respir Crit Care Med. 2018 Apr 1;197(7):961-963.
3. Ballester E, Badia JR, Hernández L, Carrasco E, de Pablo J, Fornas C, et al. Evidence of the effectiveness of continuous positive airway pressure in the treatment of sleep apnea/hypopnea syndrome. Am J Respir Crit Care Med. févr 1999;159(2):495-501

9.2 STATISTICAL METHODS USED

The final statistical analysis plan will be approved before the final database lock, which will be carried out in accordance with SOP RCDMS.MOP.009 of CHU Grenoble Alpes, following standard data management procedures and as per the pre-specified specifications. The general approach to data analysis is described below.

Populations studied and handling of missing data:

The analysis of the primary endpoint will be conducted based on the intention-to-treat (ITT) principle for superiority hypotheses, meaning that all randomized patients must be included in the analysis and analyzed in the arm to which they were initially assigned. However, this requires complete data for all randomized patients, which is rarely achievable in practice. This therefore implies imputing missing data, which we propose to do in sensitivity analyses (see below).

Conversely, for the primary analysis, we propose an analysis on the modified intention-to-treat (mITT) population, which will include patients for whom the following protocol deviations are observed:

- Use of the device in the Sunrise arm not in accordance with recommendations;
- Follow-up visits not completed.

In contrast, the non-inferiority hypothesis (level 1 of the hierarchy) will be tested in the primary analysis on the per-protocol population, which is more conservative in this case.
We therefore propose the following strategy[1,2] :

- Minimize attrition as much as possible by limiting constraints (number of on-site visits, time required to complete questionnaires, etc.);
- Continue patient follow-up and endpoint data collection even in cases of protocol deviations, particularly for remote collection of the ESS at 3 months after diagnosis;
- Consider sensitivity analyses on the per-protocol population (for superiority hypotheses) or mITT (for the non-inferiority hypothesis), as well as on the ITT population (for both superiority and non-inferiority hypotheses) after replacing missing data using multiple imputation methods, provided the assumption of data missing at random (MAR) is credible.

1. Lavange & Permutt, A regulatory perspective on missing data in the aftermath of the NRC report, *Stat in Med* 2016
2. Permutt, Sensitivity analysis for missing data in regulatory submissions, *Stat in Med* 2016

Description of the data:

For each of the two groups, qualitative variables will be described by their frequency and percentage, and quantitative variables by their mean and standard deviation, or median and interquartile range if they do not follow a normal distribution. The normality of quantitative variables will be assessed by

graphical checks and the Shapiro-Wilk test. For the primary and secondary outcomes analyzed using linear models, if the assumptions are not met, data transformation will be considered.

Analysis of the primary endpoint:

Several primary endpoints will be tested sequentially, following a predefined hierarchy (see Figure 8 below). In order to control the inflation of the alpha risk due to multiple testing, the next level in the hierarchy will only be tested if the null hypothesis at the previous level is rejected. For each level of the hierarchy, a global alpha risk of 0.05 will be applied. It should be noted that at level 1, both null hypotheses must be rejected simultaneously (each with an alpha risk of 0.05) in order to proceed to testing the subsequent levels.

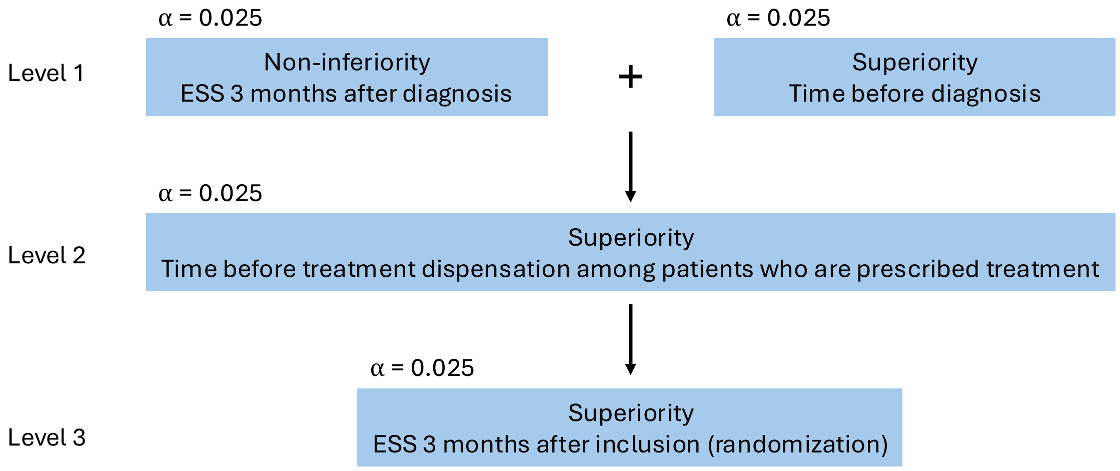

Figure 8 Predefined hierarchy for the testing of primary endpoints.

Level 1 analysis (non-inferiority of ESS) will be conducted using a **one-sided t-test**; a p-value < 0.025 will be considered statistically significant. Simultaneously, superiority regarding the time between inclusion and the announcement of the diagnosis will be tested using **a two-sided Student's t-test**; a p-value < 0.05 will be considered statistically significant. If the assumptions of the t-test are not met, a Mann-Whitney test will be used. Since the post-inclusion follow-up period is a maximum of 18 months, it is possible that the diagnosis will not be known at the end of the follow-up. We expect this situation to be rare (<5% of patients). In this case, the time to diagnosis will be considered as 18 months. If this situation turns out to be more frequent, a survival analysis using a log-rank test will be considered.

Level 2 will also be analyzed using a two-sided t-test; a p-value < 0.05 will be considered statistically significant. If the assumptions of the t-test are not met, a Mann-Whitney test will be used.

Level 3 will involve testing the superiority of the Sunrise diagnostic solution compared to the reference method on the ESS measured 3 months after randomization, adjusted for the initial ESS (at inclusion),

using an analysis of covariance (ANCOVA). If the data distribution is not normal, a transformation will be considered. A p-value < 0.05 will be considered statistically significant.

Analysis of secondary endpoints:

Comparisons of continuous variables (e.g., quality-of-life questionnaires, work productivity questionnaire) measured at inclusion (Day 0) and 3 months post-inclusion (randomization) will be performed using a two-sided ANCOVA, adjusted for the baseline value. A p-value < 0.05 will be considered statistically significant.

Other continuous variables (e.g., adherence) will be analyzed using a Student's t-test if assumptions are met, or a Mann-Whitney test if the variables do not follow a normal distribution. A p-value < 0.05 will be considered statistically significant.

Repeatability of the Sr-RDI index measurement for OSAHS severity at home in the experimental arm will be assessed using the within-subject coefficient of variation (wsCV) and the intra-class correlation coefficient (ICC). A wsCV < 10% and an ICC > 0.85 will be considered excellent.

In the reference arm, the agreement between the Sunrise diagnostic solution and the reference method (PSG) in measuring respiratory events will be evaluated using the Bland-Altman method. The diagnostic performance of the Sunrise solution compared to the reference method for detecting OSAHS at the recommended thresholds of 5 and 15 respiratory events per hour of sleep will be evaluated using sensitivity and specificity, in the PSG arm.

Analysis of health economic evaluation criteria

Cost analysis

From a statistical perspective, costs related to the efficiency analysis and the ICUR will be compared using Student's t-tests (for mean comparisons) or Wilcoxon tests (for distribution comparisons), depending on the data distribution.

QALY calculation

Utilities will be calculated from the EQ-5D-5L questionnaire, using utility scores validated in the French population (published utility tables [2])). QALYs will be calculated as follows, based on a 3-month post-

[2] Luiz Flavio Andrade; Kristina Ludwig ;· Juan Manuel Ramos Goni ; Mark Oppe ; Gérard de Pouvourville ; A French Value Set for the EQ‑5D‑5L ; PharmacoEconomics ; 8 janvier 2020.

diagnosis follow-up with EQ-5D-5L measurements at inclusion (0), at 3 months post-inclusion (pi), and at 3 months post-diagnosis (pd):

QALY (t0–t3pd) = [(U(t0) + U(t3pi))/2] × (t3pi – t0) + [(U(t3pi) + U(3pd))/2] × (3pd – t3pi)

Where t = time in years (e.g., t6 = 0.5); U = utility score

Efficiency ratio

The cost-utility ratio will take the following form:

ICUR=cost differential (ΔC) / QALY differential (ΔU) = (C_sunrise – C_ref) / (QALY_sunrise – QALY_ref)

Initially, a crude estimate of this ratio will be calculated. The confidence interval for the ICUR will be estimated using bootstrap methods, and possibly by a method based on Fieller's theorem.

Next, we will estimate the incremental net benefit (INB = ΔE × λ – ΔC), where λ is the maximum value the payer is willing to pay for one unit of effectiveness, adjusted for the randomization stratification factors and other potential confounders using multiple linear regression models.

The probability of cost-effectiveness (PCE) of the Sunrise device compared to PSG will be calculated based on the INB distribution for a given value of λ. A cost-effectiveness acceptability curve (CEAC) representing the PCE as a function of λ will be developed for λ values ranging from €0 to €100,000.

10 BENEFIT / RISK RATIO

10.1 EXPECTED BENEFITS

For patients randomized to the experimental arm, the Sunrise diagnostic solution, being more accessible than PSG, is expected to allow quicker access to care while ensuring high-quality management. The patient becomes more involved in their own diagnosis, and the ability to easily repeat recordings at home is a considerable advantage.

For patients randomized to the PSG arm, as this involves standard care procedures, no benefit related to participation in this clinical investigation is expected.

10.2 RISKS RELATED TO THE CLINICAL INVESTIGATION

The additional procedures carried out as part of this study (completion of questionnaires) are non-invasive.

Polysomnography (PSG) is a standard care procedure (which may simply cause some discomfort falling asleep due to the various sensors). Regarding the Sunrise solution, this CE-marked device is used within its intended purpose. The use of the Sunrise device may lead to adverse effects or symptoms such as skin irritation (see section 11.4).

No additional risks are introduced by this study.

10.3 BENEFIT / RISK BALANCE

The benefit/risk balance is favorable.

10.4 STUDY LIMITATIONS

The inclusion criteria being comparable to those of routine practice, the study results are expected to be equivalent to those observed in real life, making the trial generalizable.
The choice of superiority criteria on sleepiness, quality of life, work productivity, and diagnostic and treatment initiation delays will help demonstrate a considerable benefit for patients.

A primary endpoint selected from hard measures of morbidity and mortality, such as major adverse cardiac events (MACE), is not appropriate given the time frame and the recommendations for conducting an Innovation Package study (see ministerial instruction).

10.5 RISK CONTROL AND MITIGATION

The risks were identified through a clinical investigation including an exhaustive literature review and a prior clinical investigation for obtaining the CE marking, representing the most up-to-date knowledge of the risks associated with the device and the procedure. Additionally, the sponsor has mitigated the risks related to the device, the procedure, and the study by taking into account the following considerations:

- Selection of experienced and competent investigators in the field of sleep medicine.
- All investigators and study team members participating in this clinical investigation will be trained on the protocol.
- Only appropriate patients will be included in the study based on inclusion/exclusion criteria comparable to those of routine practice.

- Ensuring that the diagnostic procedure and patient follow-up are conducted frequently according to the protocol and as consistent as possible with routine practice.
- Providing patients with additional personalized care and a rapid appointment/teleconsultation in case of adverse events or questions regarding device use.
- Providing continuous technical and clinical support to the study staff throughout the duration of the study.
- Adverse effects inherent to the device are clearly disclosed in the patient information leaflet so that patients can make an informed decision about participating in the study.
- Instructions for device use are provided to the patient.

11 VIGILANCE AND SAFETY

11.1 DÉFINITIONS

Adverse event: any harmful occurrence, any unintended illness or injury, or any unfortunate clinical sign, including an abnormal laboratory finding, in participants, users, or other persons during a clinical investigation, whether or not related to the medical device under investigation.

Serious adverse event: any adverse event that has resulted in:

a) death;

b) a serious deterioration in the participant's health, which leads to:

i) a life-threatening illness or injury;

ii) permanent impairment of a bodily structure or function;

iii) hospitalization or prolonged hospitalization of the participant;

iv) medical or surgical intervention to prevent life-threatening illness or injury or permanent impairment of a bodily structure or function;

v) chronic disease;

c) fetal distress, fetal death, congenital physical or mental disabilities, or congenital malformation.

Adverse device effect: any adverse event related to the use of the medical device under investigation. This definition includes any adverse event resulting from deficiencies or inadequacies in the instructions for use, deployment, implantation, installation, operation, or any malfunction of the medical device under investigation. It also includes any event resulting from user error or intentional misuse of the medical device under investigation. This includes the comparator if it is a medical device.

Serious adverse device effect: an adverse device effect that results in any of the consequences characteristic of a serious adverse event.

Device deficiency: any defect regarding identity, quality, durability, reliability, safety, or performance of a device under investigation, including any malfunction, user error, or defect in the information provided by the manufacturer.

Incident: any malfunction or deterioration in the characteristics or performance of a device available on the market, including user error due to ergonomic features, as well as any defect in the information provided by the manufacturer and any undesirable side effect.

Serious incident: any incident that has caused or may have caused or may cause:

a) death of a patient, user, or any other person;

b) serious temporary or permanent deterioration of the health of a patient, user, or any other person;

c) a serious threat to public health.

Safety corrective action: any corrective measure taken by a manufacturer for technical or medical reasons to prevent or reduce the risk of a serious incident related to a device available on the market.

11.2 INVESTIGATOR RESPONSIBILITIES

For this study, the investigator is required to report to the sponsor any serious adverse events and adverse effects related to the medical device under investigation. It is also the responsibility of the investigator and the research team to report any defects associated with medical devices distributed by SUNRISE SA.

Investigators must gather all available information to determine the start date, end date, intensity, causal relationship, severity, seriousness, measures taken, and the progression of the reported event.

The investigator must also send all requested supporting documents (pseudonymized) in accordance with the sponsor’s request.

The method for reporting device-related adverse events and defects will be a paper adverse event or defect form to be completed, dated, and signed. The form must be sent by email to sunsas@icuresearch.eu.

Events must be reported for all patients from the date of consent throughout the patient's follow-up period as defined by the study. If a reported event is not resolved by the end of the study, the event will be considered ongoing/unresolved, and no further action will be required from the investigator.

The management of any adverse event is at the sole discretion of the investigator and must comply with current good medical practice. Any medication administered to treat an adverse event must be recorded in the patient's eCRF.

11.2.1 Evaluation of the causal relationship

An assessment of causality is carried out for every reported serious adverse event. The rating method used is as follows:

- **No relationship**: The relationship with the device or procedures can be excluded if the event occurs within a timeframe incompatible with the study and/or there is sufficient information showing the observed reaction is unrelated to the study and/or there is a plausible alternative explanation, etc.
- **Possible relationship**: The relationship with the use of the device or with the procedures is weak but cannot be entirely excluded. Alternative causes are also possible (e.g., an underlying or concomitant disease/clinical condition and/or the effect of another device, drug, or treatment). Cases where the relationship cannot be assessed or where no information has been obtained should also be classified as "possible."
- **Probable relationship**: The relationship with the use of the device or with the procedures appears relevant and/or the event cannot reasonably be explained by another cause.
- **Determined relationship**: The serious adverse event is associated with the device, comparator, or procedures beyond any reasonable doubt when the event is a known side effect of the product category to which the device belongs or of similar devices and procedures; the event has a temporal relationship with the use/application of the device or procedures; the event involves a body part or organ where the device or investigative procedures apply or affect; the serious adverse event follows a known response pattern to the medical device (if the pattern is known); stopping the use of the medical device (or reducing the level of activation/exposure) and reintroducing its use (or increasing the level of activation/exposure) impact the serious adverse event (when clinically feasible); other possible causes (e.g., underlying or concomitant disease/clinical condition and/or effect of

another device, drug, or treatment) have been adequately ruled out; the patient harm is due to misuse; the event depends on a false result given by the device used for diagnosis.

Adverse events with a possible, probable, or determined relationship are considered related to the medical device and/or procedures.

Investigators must distinguish between adverse events related to the Sunrise device and those related to the procedures (any procedure specific to the clinical investigation). An adverse event may be related to both the procedure and the device.

Procedure complications are considered unrelated if these procedures would have been applied to patients even without the use/application of the Sunrise device.

In some specific cases, the event may not be adequately assessed due to insufficient or conflicting information and/or data that cannot be verified or completed. Investigators will make every effort to define and categorize the event to avoid these situations.

11.2.2 Evaluation of the severity of the event

The severity of an adverse event is assessed by the investigator using the following categories:

- Mild: Signs and symptoms that can be easily tolerated. Symptoms that can be ignored.
- Moderate: Symptoms cause discomfort but are tolerable; they cannot be ignored and affect concentration.
- Severe: Symptoms affect usual daily activities.

11.2.3 Evaluation of the progression of the event

The investigator will assess the progression of all reported events during the study as one of the following:

- Resolved without sequelae
- Resolved with sequelae
- Ongoing
- Death
- Not resolved
- Unknown

11.3 METHODS OF REPORTING BY THE INVESTIGATOR TO THE SPONSOR

The events to be reported as mentioned in section 11.2 and any device defects occurring throughout the study must be notified to the sponsor immediately, and no later than 3 working days after becoming aware of the event.

11.4 REPORTING PROCEDURES OF THE SPONSOR/MANUFACTURER TO THE COMPETENT AUTHORITIES

The sponsor of this study, SUNRISE SA, is also the manufacturer of the Sunrise medical device under investigation in SUNSAS.

In accordance with European Regulation 2017/745 on medical devices (MDR), the sponsor notifies the competent authorities of any serious adverse event when there is a causal relationship between this serious adverse event and the preceding investigation procedure. All events resulting in death or immediate risk of death, serious injury or illness that require rapid corrective action for participants/patients, users, or others, or any new information about these cases must be reported without delay (immediately), and no later than 2 calendar days from the day the sponsor became aware of the event to be reported or new information regarding an already reported event. Other serious adverse events to be reported to the authorities or any new information/updates concerning them must be reported without delay (immediately), and no later than 7 calendar days from the day the sponsor became aware of the event to be reported or new information regarding an already reported event.

11.5 EXPECTED ADVERSE EVENTS OR EFFECTS

The expected adverse effects are irritation, itching, and redness of the skin.

12 DATA MANAGEMENT

12.1 DATA COLLECTION AND PROTECTION

An electronic Case Report Form (eCRF) will be used for the study. The MARS eCRF collection platform holds the necessary certifications for hosting health data in France (HDS) and ensures compliance with the European GDPR regulations on data protection. Individuals responsible for completing the eCRF must be clearly identified in the delegation of tasks document.

Data will be entered into the eCRF progressively as it is collected during the study, by the investigator or an authorized individual. Each change will be recorded in the eCRF (audit trail). The eCRF is secured

and accessible only via a login and password. The investigator is required to sign off on the data entered.

For the questionnaires, patients may complete them electronically using their smartphone, tablet, or computer via a dedicated digital platform. The data will then be automatically transferred into the MARS eCRF.

In the event of missing, illegible, or inconsistent data, query lists will be sent to the relevant investigators, and corrections must be made in the eCRF.

Data recorded during this study will be processed electronically at CHU GRENOBLE ALPES in accordance with Law No. 2018-493 of June 20, 2018, concerning the protection of personal data.
Data processing is carried out under Article 9 of EU Regulation 2016/679.
This study falls within the scope of the "Reference Methodology" (MR-001) as per Article 54, paragraph 5, of the amended Law of January 6, 1978, relating to information technology, files, and civil liberties. The sponsor has submitted a declaration of compliance with this reference methodology to the CNIL.

12.2 Definition of Source Data

Source data includes all information contained in original records or certified copies of original records that document clinical findings, observations, or any other information related to a clinical investigation, necessary for the reconstruction and evaluation of the clinical investigation. This also includes the following data entered directly into the eCRF in electronic format:

- Responses to the ESS, EQ5D-5L, QSQ, SF-36, and WPAI:SAS questionnaires
- Medical history
- Ongoing treatments
- Measurement of anthropometric parameters: sex, age (month and year of birth), weight (kg), height (cm), body mass index (BMI) in kg/m², neck circumference (cm), waist circumference (cm), and hip circumference (cm)
- Measurement of blood pressure (mmHg) and heart rate (bpm)
- Risk factors including assessment of smoking status, alcohol consumption, and respiratory allergies
- Clinical signs suggesting suspected sleep apnea syndrome
- Patient eligibility criteria

- Visit dates

12.3 Data management

Data management will consist of the following steps:

- Drafting a data management plan, including a data validation specification plan ;
- Drafting an annotated case report form (CRF) ;
- Programming and validating consistency checks ;
- Correcting the database ;
- Generating listings ;
- Reviewing the data.

12.4 Data review

A data review meeting prior to database lock will be held either in person or remotely once all data have been collected, entered, and validated. At a minimum, the sponsor, the data manager, the statistician, and the study monitor will attend.

The purpose of this meeting is to:

- • Review the study conduct conditions;
- • Specify protocol-defined deviations;
- • Determine the analysis populations;
- • Review the statistical analysis plan.

12.5 Database lock

The database lock will be performed once all queries are resolved, corrections integrated, and monitoring completed. A copy of the locked database files will be sent to the sponsor and the statistician for analysis. A signed database lock form will be provided.

Once the database is locked, any modification can only be made using database correction forms approved by the sponsor. If the database needs to be corrected, the locking/unlocking of the database must be clearly documented in the study file.

13 CONFIDENTIALITY AND PSEUDONYMIZATION OF PERSONAL DATA

In accordance with the applicable legislative provisions (articles L.1121-3 and R.5121-13 of the Public Health Code), persons with direct access to the source data will take all necessary precautions to ensure the confidentiality of information related to the experimental devices, the research, the participants involved, particularly concerning their identity and the results obtained. These persons, like the investigators themselves, are bound by professional secrecy.

During or after the clinical investigation, the data collected on the participants and transmitted to the sponsor by the investigators (or any other specialized personnel) will be pseudonymized. The names and addresses of the individuals concerned must never appear in clear text. It is the Investigator's responsibility to keep sufficient information to identify the patients participating in the trial and their records, and to be able to provide this information to the competent authorities or the sponsor, if necessary. All documents identifying research participants generated during this study must be treated confidentially and must not be disclosed to persons not directly involved in the study without the patient's written authorization.

Subject coding method: PAXXXXX with PA for Patient Anonymous followed by the unique patient number, which may include up to 5 digits.

14 SECURITY OF SUNRISE DEVICE DATA

Upon registration, the patient must explicitly give their consent before their registration data is temporarily stored on the phone and then sent to the application programming interface (API) for recording. Without this consent, these data are neither stored on the phone nor sent to the API, and the user account cannot be created.

For communication between the sensor and the mobile application, the communication protocol used is low energy Bluetooth. By definition, one intrinsic characteristic of this type of communication is that once connected, it can no longer be detected by another device. Additionally, its low power consumption reduces the transmission range and the distance at which the device can be detected. These two factors secure the connection between the sensor and the mobile app.

At the end of the night, a wireless connection (WiFi or 3G/4G) is necessary to establish communication between the smartphone and the cloud server. The recording data are temporarily stored on the phone during the night but are sent to the cloud storage space once the night is over.

All transfers and communications with the API are conducted via the HTTPS protocol (HyperText Transfer Protocol Secure), an encrypted and secure communication protocol.
The cloud service used, Google Cloud, holds the necessary certifications to allow the hosting of health data in France (HDS) and ensures compliance with the European RGPD guidelines on data protection as well as adherence to the General Security Policy for Health Information Systems (PGSSI-S).

The analysis data for each patient are accessible to the investigating physicians at the centers where the patients were enrolled. These data are not stored permanently on the patient's phone.

15 CONFIDENTIALITY

All information (oral or written) and unpublished documents provided to the investigators, including this protocol and the collected data, are the exclusive property of SUNRISE SA. This information may not be transferred or disclosed to any unauthorized third party by the investigators and/or any other person working under their responsibility without the prior written consent of SUNRISE SA.
Investigators must treat all information acquired or inferred during the study as confidential and take all necessary measures to prevent any breach of confidentiality, except for information that must be disclosed in accordance with the law.

16 CONTROL AND QUALITY ASSURANCE

16.1 INSTRUCTIONS FOR DATA COLLECTION

The individuals responsible for data collection in the electronic case report form (eCRF) will be clearly identified in the task delegation form.
Erroneous data found in the case report forms will be clearly crossed out, and the new data will be recorded next to the crossed-out information, accompanied by the initials, date, and, if applicable, a justification provided by the investigator or authorized person who made the correction.

16.2 QUALITY CONTROL/MONITORING OF DATA

In accordance with applicable regulations, ISO 14155, and the study monitoring guide, the clinical research associates (CRAs) appointed by the sponsor will contact each site before the study begins to train the principal investigator and their research team on the clinical investigation protocol, study

requirements, and their responsibilities in order to meet regulatory, ethical, and sponsor requirements.

The monitor must be independent from the participating site and is not authorized to enter or modify data in source documents or the eCRF. On-site or remote monitoring visits will be conducted periodically to verify, among other things, that:

- The data are authentic, accurate, and complete.
- The safety and rights of participants are protected.
- The study is conducted in accordance with the approved protocol, GCP (ISO 14155), and all applicable regulatory requirements.

The investigator agrees to grant the monitor access to all source documents in accordance with applicable legislative and regulatory provisions (Articles L.1121-3 and R.5121-13 of the Public Health Code) and to provide sufficient time, a dedicated space, and qualified staff for monitoring visits. The monitor will review the eCRF and perform source data verification (SDV) according to the monitoring guide. By SDV, we mean verifying eCRF entries by comparing them with the source data made available by the investigator for this purpose.

The monitor will assess and summarize the results of each monitoring visit in written reports, identifying any issues and specifying recommendations to resolve observed problems.

At the end of the study (or earlier if prematurely terminated), the monitor will conduct site close-out activities in accordance with applicable regulations, GCP, and the study monitoring guide.

16.3 AUDIT AND INSPECTION

An audit conducted at the sponsor's request or an inspection carried out by health authorities may take place at any time during or after the study (from protocol development to the publication of results and the archiving of data used or produced in the research). Its purpose is to ensure the quality of the research (compliance with the protocol and GCP), the validity of its results, and adherence to applicable laws and regulations.

Investigators must provide auditors/inspectors with direct access to source and medical data and any relevant documents related to the conduct of the clinical study. They must also allocate sufficient time

to discuss the study conduct, any findings identified, and implement corrective and/or preventive measures to address those findings. The investigator must inform the sponsor as soon as they become aware of the audit or inspection.

Investigators agree to comply with the requirements of the sponsor and the competent authority regarding any audit or inspection of the research.

17 RULES FOR STOPPING THE CLINICAL INVESTIGATION

The clinical investigation will be considered completed when the last visit of the last patient scheduled by the protocol has been conducted. The sponsor will issue a trial end declaration within 90 days following the end of the clinical investigation.

The sponsor and regulatory authorities reserve the right to temporarily suspend or terminate the clinical investigation at any time for reasons such as (but not limited to) safety concerns, ethical issues, or serious non-compliance. If the sponsor determines such action is necessary, the reasons will be discussed with the investigator.

If the clinical investigation is suspended or stopped for safety reasons, the sponsor will promptly inform all investigators and/or institutions participating in the study. If necessary, the sponsor will also promptly notify the competent regulatory authorities of the suspension or termination and the reasons for it. When required by applicable regulations, the investigator will inform the ethics committee as soon as possible and provide the reason(s) for the suspension or termination of the study.

The sponsor may also stop patient enrollment at a participating center at any time, for example, for the following reasons:

- Investigator’s inability to enroll patients according to the planned schedule,
- Lack of signed consent,
- Major protocol violations,
- Incomplete or incorrect data.

18 ETHICAL AND REGULATORY CONSIDERATIONS

Before the start of the study, the sponsor will obtain the favorable opinion of the Ethics Committee (CPP) and inform the appropriate competent authorities. The investigator will comply with all additional requirements imposed by the CPP or the competent authorities, as applicable.

The study will be conducted in accordance with all applicable regulatory requirements, and in accordance with the Declaration of Helsinki (amended in Fortaleza in 2013, full version at http://www.wma.net), and the recommendations of Good Clinical Practice (GCP - ISO 14155).
Information about the study will be published before the start of patient recruitment on the publicly accessible website https://clinicaltrials.gov/.

19 PROTOCOL DEVIATIONS

Except in emergency situations requiring the implementation of specific therapeutic actions, the investigator(s) agree(s) to fully comply with the protocol, particularly regarding the collection of consent and the notification and follow-up of serious adverse events.

The clinical investigation is conducted in accordance with this protocol. The investigator is not authorized to deviate from the protocol, except in emergency situations to protect the rights, safety, and well-being of patients. As soon as possible, any deviation or modification implemented and the reasons for it must be reported to the Ethics Committee (CPP) (in accordance with local regulations) and to the sponsor.

Furthermore, the investigator (or an authorized member of the study research team) must document all protocol deviations in a dedicated Excel spreadsheet. A deviation is considered major if it affects the rights, safety, and well-being of the patient, or the scientific integrity of the clinical investigation. In the case of a major deviation, it must be reported within two business days after becoming aware of it.

A protocol deviation may ultimately lead to the patient's withdrawal from the study if it poses a risk to them. This decision will be made after consultation between the sponsor and the investigator.

20 AMENDMENT TO THE PROTOCOL

Any substantial modification, meaning any change likely to have a significant impact on the protection of individuals, on the validity conditions and outcomes of the clinical investigation, on the quality and safety of the devices under investigation, on the interpretation of scientific documents supporting the conduct of the clinical investigation, or on the procedures for conducting it, must be subject to a written amendment submitted to the Ethics Committee (CPP) and the competent regulatory authorities, where applicable. Investigators must obtain a favorable opinion from the CPP and competent authorities, where applicable, prior to its implementation.
All modifications are validated by the sponsor before submission.

All protocol amendments must be communicated to all investigators participating in the clinical investigation. Investigators commit to comply with their content.
Any amendment that modifies patient management or the benefits, risks, and constraints of the clinical investigation requires a new information note and a new consent form, for which consent collection follows the same procedure as previously described.

21 INSURANCE

The company SUNRISE SA, sponsor of this clinical investigation, has taken out a civil liability insurance contract with CNA.

22 PRESERVATION OF DOCUMENTS AND RESEARCH-RELATED DATA

At the end of the study or following the interruption of the study/site, the following documents related to this clinical investigation will be archived in a safe and secure location by the participating centers for a period of 15 years in accordance with Good Clinical Practice:

- The protocol and any amendments to the protocol
- The case report forms
- The source documents of patients who have signed a consent form
- All other documents and correspondence related to the research
- The original signed informed consent forms of the participants

All these documents are under the responsibility of the investigator for the duration of the regulatory archiving period.

No relocation or destruction may be carried out without the promoter's agreement. At the end of the regulatory archiving period, the promoter will be consulted regarding destruction. All data, documents, and reports may be subject to audit or inspection.

23 STEERING COMMITTEE

The role of the steering committee is to ensure overall supervision of the study. The committee will review any questions or issues that may arise during the conduct of the study at the participating centers. In particular, the steering committee will closely monitor the recruitment rate, patient compliance with the trial, dropout rate, and the quality of data management. The steering committee will also be authorized to receive and discuss any proposals for ancillary studies and the use of the database.

It will be composed of at least four study investigators (including the coordinating investigator). Representatives of the sponsor, statisticians, health economists, etc., may participate in meetings if deemed appropriate.

The steering committee will meet at least once a year, although there may be periods when more frequent meetings are necessary.

24 RULES RELATING TO PUBLICATION

24.1 FINAL STUDY REPORT

The sponsor, in collaboration with the coordinating investigator, will prepare the final study report. The final clinical investigation report will be prepared in accordance with ISO 14155 standards whether the trial is completed or prematurely terminated. The sponsor and the coordinating investigator will sign the final report after review.

The final report or a summary will be submitted to the Ethics Committee (CPP) and the competent regulatory authorities in accordance with local regulations. The sponsor will also provide the investigator with a complete summary of the study results.

24.2 SCIENTIFIC COMMUNICATIONS

The study results will be made public. The principal investigator and the sponsor will mutually agree on the choice of publisher for the main results publication. International writing and publication guidelines (The Uniform Requirements for Manuscripts by ICMJE, April 2010) will be taken into account to determine the order of authors.

Any written or oral communication of the results from this clinical investigation must receive prior written approval from the sponsor.

24.3 COMMUNICATION OF RESULTS TO PATIENTS

In accordance with Law No. 2002-303 of March 4, 2002, patients may be informed, upon their request, of the overall results of the clinical investigation.

25 PROCEDURE FOR OBTAINING INFORMED CONSENT

Verbal and written information will be provided to patients detailing the clinical trial and what their participation entails. This information will specify:

- the nature of the clinical trial;
- the details of what will be expected from the patient;
- the implications and constraints of the protocol;
- the potential adverse effects/risks associated with participation in the trial.

It will be clearly stated that the patient is free to withdraw from the trial at any time and for any reason, without affecting the quality of care they receive and without any obligation to provide a reason for their withdrawal.

The patient will have sufficient time to ask the investigator questions about the clinical trial and, if desired, will be given a reasonable reflection period of 24 hours to decide whether to participate in the clinical trial.

When the subject agrees to participate in the clinical investigation, they will date and sign a consent form together with the investigator.

The different copies of the information sheet and the consent form are then distributed as follows:

- One copy of the information sheet and signed consent is given to the patient.
- One copy is kept by the investigator in a secure location inaccessible to third parties.

26 AGREEMENT AND FUNDING

The agreement between SUNRISE SA and the participating center must be established before any patient is enrolled in the clinical study and before any procedure related to the protocol is carried out.

The principal investigator must comply with all terms, conditions, and obligations described in the agreement. In case of any discrepancy between this clinical investigation protocol and the agreement, the terms of the protocol shall prevail regarding the conduct of the study. For any other matters unrelated to the conduct of the investigation, the terms of the agreement shall prevail.

Routine care is covered by Health Insurance. A flat rate of 300 euros is covered by Health Insurance for the Innovation Package (for patients in the Sunrise arm). SUNRISE SA, as the sponsor, covers the costs/overheads related to the SUNSAS study.

27 BIBLIOGRAPHY

1. Benjafield AV, Ayas NT, Eastwood PR, Heinzer R, Ip MSM, Morrell MJ, et al. Estimation of the global prevalence and burden of obstructive sleep apnoea: a literature-based analysis. Lancet Respir Med. août 2019;7(8):687-98.
2. Lévy P, Kohler M, McNicholas WT, Barbé F, McEvoy RD, Somers VK, et al. Obstructive sleep apnoea syndrome. Nat Rev Dis Primers. 25 2015;1:15015.
3. sahos_-_fiche_de_bon_usage.pdf [Internet]. [cité 3 sept 2019]. Disponible sur: https://www.has-sante.fr/upload/docs/application/pdf/2014-11/sahos_-_fiche_de_bon_usage.pdf
4. Tregear S, Reston J, Schoelles K, Phillips B. Obstructive Sleep Apnea and Risk of Motor Vehicle Crash: Systematic Review and Meta-Analysis. J Clin Sleep Med. 15 déc 2009;5(6):573-81.
5. Garbarino S, Guglielmi O, Sanna A, Mancardi GL, Magnavita N. Risk of Occupational Accidents in Workers with Obstructive Sleep Apnea: Systematic Review and Meta-analysis. Sleep. 1 juin 2016;39(6):1211-8.
6. CEPP-4824_INSPIRE_28_juin_2016_(4824)_avis.pdf [Internet]. [cité 3 sept 2019]. Disponible sur: https://www.has-sante.fr/upload/docs/evamed/CEPP-4824_INSPIRE_28_juin_2016_(4824)_avis.pdf
7. Société de Pneumologie de Langue Française, Société Française d'Anesthésie Réanimation, Société Française de Cardiologie, Société Française de Médecine du Travail, Société Française d'ORL, Société de Physiologie, et al. [Recommendations for clinical practice. Obstructive sleep apnea hypopnea syndrome in adults]. Rev Mal Respir. sept 2010;27(7):806-33.
8. The Cost of Fatigue [Internet]. Advanced Sleep Medicine Services, Inc. 2015 [cité 21 oct 2019]. Disponible sur: https://www.sleepdr.com/the-sleep-blog/the-cost-of-fatigue/
9. Évaluation clinique et économique des dispositifs médicaux et prestations associées pour prise en charge du syndrome d'apnées hypopnées obstructives du sommeil (SAHOS) [Internet]. Haute Autorité de Santé. [cité 20 août 2019]. Disponible sur: https://www.has-sante.fr/jcms/c_1761818/fr/evaluation-clinique-et-economique-des-dispositifs-medicaux-et-prestations-associees-pour-prise-en-charge-du-syndrome-d-apnees-hypopnees-obstructives-du-sommeil-sahos
10. Place et conditions de réalisation de la polysomnographie et de la polygraphie respiratoire dans les troubles du sommeil - Rapport d'évaluation [Internet]. Haute Autorité de Santé. [cité 30 août 2019]. Disponible sur: https://www.has-sante.fr/jcms/c_1056842/fr/place-et-conditions-de-realisation-de-la-polysomnographie-et-de-la-polygraphie-respiratoire-dans-les-troubles-du-sommeil-rapport-d-evaluation
11. Collop NA. Scoring variability between polysomnography technologists in different sleep laboratories. Sleep Med. janv 2002;3(1):43-7.

12. Arnardottir ES, Verbraecken J, Gonçalves M, Gjerstad MD, Grote L, Puertas FJ, et al. Variability in recording and scoring of respiratory events during sleep in Europe: a need for uniform standards. J Sleep Res. 2016;25(2):144-57.

13. Corral-Peñafiel J, Pepin J-L, Barbe F. Ambulatory monitoring in the diagnosis and management of obstructive sleep apnoea syndrome. Eur Respir Rev. 1 sept 2013;22(129):312-24.

14. Randerath W, Bassetti CL, Bonsignore MR, Farre R, Ferini-Strambi L, Grote L, et al. Challenges and perspectives in obstructive sleep apnoea: Report by an ad hoc working group of the Sleep Disordered Breathing Group of the European Respiratory Society and the European Sleep Research Society. Eur Respir J. 2018;52(3).

15. Gray EL, Barnes DJ. Beyond the thermistor: Novel technology for the ambulatory diagnosis of obstructive sleep apnoea. Respirology. 2017;22(3):418-9.

16. Escourrou P, Grote L, Penzel T, Mcnicholas WT, Verbraecken J, Tkacova R, et al. The diagnostic method has a strong influence on classification of obstructive sleep apnea. J Sleep Res. déc 2015;24(6):730-8.

17. Maury G, Senny F, Cambron L, Albert A, Seidel L, Poirrier R. Mandible behaviour interpretation during wakefulness, sleep and sleep-disordered breathing. J Sleep Res. déc 2014;23(6):709-16.

18. Martinot J-B, Senny F, Denison S, Cuthbert V, Gueulette E, Guénard H, et al. Mandibular movements identify respiratory effort in pediatric obstructive sleep apnea. J Clin Sleep Med. 15 avr 2015;11(5):567-74.

19. Miyamoto K, Ozbek MM, Lowe AA, Sjöholm TT, Love LL, Fleetham JA, et al. Mandibular posture during sleep in patients with obstructive sleep apnoea. Arch Oral Biol. août 1999;44(8):657-64.

20. Senny F, Destiné J, Poirrier R. Midsagittal jaw movement analysis for the scoring of sleep apneas and hypopneas. IEEE Trans Biomed Eng. janv 2008;55(1):87-95.

21. Senny F, Maury G, Cambron L, Leroux A, Destiné J, Poirrier R. The sleep/wake state scoring from mandible movement signal. Sleep Breath. juin 2012;16(2):535-42.

22. Pépin J-L, Letesson C, Le-Dong NN, Dedave A, Denison S, Cuthbert V, et al. Assessment of Mandibular Movement Monitoring with Machine Learning Analysis for the Diagnosis of Obstructive Sleep Apnea. JAMA Netw Open. 3 janv 2020;3(1):e1919657.

23. Bratton DJ, Gaisl T, Schlatzer C, Kohler M. Comparison of the effects of continuous positive airway pressure and mandibular advancement devices on sleepiness in patients with obstructive sleep apnoea: a network meta-analysis. Lancet Respir Med. nov 2015;3(11):869-78.

24. Crook S, Sievi NA, Bloch KE, Stradling JR, Frei A, Puhan MA, et al. Minimum important difference of the Epworth Sleepiness Scale in obstructive sleep apnoea: estimation from three randomised controlled trials. Thorax. 2019;74(4):390-6.

25. Heinzer R, Vat S, Marques-Vidal P, Marti-Soler H, Andries D, Tobback N, et al. Prevalence of sleep-disordered breathing in the general population: the HypnoLaus study. Lancet Respir Med. avr 2015;3(4):310-8.

26. American Academy of Sleep Medicine. *International Classification of Sleep Disorders*. 3rd ed. Darien, IL: American Academy of Sleep Medicine; 2014.

27. Bland JM, Altman DG. Applying the right statistics: analyses of measurement studies. Ultrasound Obstet Gynecol. 2003;22(1):85-93.
