# Supplementary material for: Time to diagnosis and treatment of obstructive sleep apnoea using mandibular jaw movement monitoring versus polysomnography: an open-label, multicentre, randomised, controlled trial
Source: Lancet Reg Health Eur. 2026 Mar 17;64:101637. doi: 10.1016/j.lanepe.2026.101637 (PMC13147807; doi:10.1016/j.lanepe.2026.101637)
Supplement: Statistical analysis plan [file mmc3.pdf]

Docusign Envelope ID: B2C41F77-2931-48C9-868E-D7E9279F8B09

SUNSAS - Statistical Analysis Plan

Validation of an integrated digital solution (Sunrise) for the automated analysis of mandibular jaw movements using artificial intelligence versus polysomnography for the diagnosis of obstructive sleep apnea: a national multicenter randomized controlled trial.

Acronym: SUNSAS.

N° ID/RCB: 2021-A01827-34.

Author

Name	Function	Date	Signature
Marc MANCEAU	Statistician	November 21, 2024	

Reviewers

Name	Function	Date	Signature
Pierre MARTINOT	CTO, Sunrise	November 19, 2024	Pierre Martinot
Jean-Louis PEPIN	Directeur Labo HP2	November 19, 2024	Jean Louis Pépin
Nathalie PREAUBERT	Health Economist	November 20, 2024	Nathalie Preaubert

Approver

Name	Function	Date	Signature
Matthieu ROUSTIT	Methodologist	November 20, 2024	Matthieu ROUSTIT

Change History

Version	Change summary	Date
V1.0	First edition of the document	18 NOV 2024

Docusign Envelope ID: B2C41F77-2931-48C9-868E-D7E9279F8B09

Table of Contents

1. Administrative Information 3
1.1. Staff Members 3
1.2. Statistical Analysis Process 4
2. Objectives of the Study 5
2.1. Study Design 5
2.2. Primary Objectives 5
2.3. Primary Endpoints 5
2.4. Secondary Objectives 5
2.5. Secondary Endpoints 6
3. Number of Patients Required 6
4. Populations of Analysis and Handling of Missing Data 7
4.1. Populations of Analysis 7
4.2. Missing Data Report 8
4.3. Imputation of Missing Data 9
5. Descriptive Analysis 10
6. Primary Objectives 11
6.1. Sequential Analysis Hierarchy 11
6.2. Non-inferiority on the ESS at 3 Months After Diagnostic Consultation 12
6.3. Superiority on the Time Between Inclusion and Diagnostic Consultation 13
6.4. Superiority on the Time Between Inclusion and Treatment Dispensation 13
6.5. Superiority on the ESS at 3 Months After Inclusion 14
7. Secondary Objectives 15
7.1. Superiority on SF36 and QSQ at 3 Months After Inclusion 15
7.2. Superiority on WPAI:SAS at 3 Months After Inclusion 17
7.3. Cost-effectiveness Analysis 17
7.4. Net Benefit for the Health Insurance of Sunrise's Diffusion in France 18
7.5. Non-inferiority on the Adherence to PAP at 3 Months After the First Use of Treatment 19
7.6. Diagnostic Accuracy of Sunrise 19
7.7. Inter-night Variability of the Number of Respiratory Events per Hour 20

1. Administrative Information

1.1. Staff Members

- Coordinating investigator:
  Pr Jean-Louis PEPIN
  Clinique Universitaire de Physiologie, Pôle Thorax et Vaisseaux,
  CHU Grenoble Alpes, 38043 Grenoble Cedex 09,
  Université Grenoble Alpes,
  Inserm U1042, Laboratoire HP2, Grenoble, France
  Tel: +33 4 76 76 87 66
  Mail: JPepin@chu-grenoble.fr

- Sponsor:
  Sunrise
  Pierre MARTINOT
  Chaussée de Marche 598/02, 5101 Namur, Belgique
  Tel: +32 81 26 11 26
  Mail: pierre@hellosunrise.com

- Sponsor representative:
  Dr Camille DUCKI
  Délégation à la Recherche Clinique et à l'Innovation,
  CHU Grenoble Alpes, Pavillon Dauphiné,
  CS 10217, 38043 Grenoble Cedex 09, France
  Chef de Projet Promoteur: Amjad UNEISI
  Tel: +33 4 76 76 81 08
  Mail: AUneisi@chu-grenoble.fr

- Clinical research organization (CRO):
  Stéphane RUCKLY
  ICUREsearch
  6B Avenue de Romans,
  38160 Saint-Marcellin, France
  Tel: +33 4 38 90 39 38
  Mail: stephane.ruckly@icuresearch.eu

- Study methodology:
  Pr Matthieu ROUSTIT
  Centre d'Investigation Clinique – Inserm CIC1406, CHU Grenoble Alpes,
  38043 Grenoble Cedex 09, Université Grenoble Alpes, Inserm U1042,
  Laboratoire HP2, Grenoble, France
  Tel: +33 4 76 76 62 36
  Mail: MRoustit@chu-grenoble.fr

Docusign Envelope ID: B2C41F77-2931-48C9-868E-D7E9279F8B09

- Data management:
  Rémi AIGUEBONNE
  CHU Grenoble Alpes, Pôle Thorax et Vaisseaux,
  38043 Grenoble Cedex 09, France
  Data Manager – Base de Données MARS
  Pôle Thorax & Vaisseaux – Laboratoire d'EFCR
  Tel: +33 4 76 76 84 81
  Mail: remi.aiguebonne@univ-grenoble-alpes.fr

- Methodology of the economic evaluation:
  Nathalie PREAUBERT
  Économiste de la santé
  Medconsult
  21 Quai Alphonse le Gallo,
  92100 Boulogne-Billancourt, France
  Tel: +33 6 89 10 86 66
  Mail: nathalie.preaubert@medconsult.fr

- Statistical analysis:
  Dr Marc MANCEAU
  Unité de Pharmacologie Clinique, Centre d'Investigation Clinique Inserm,
  CIC1406, CHU Grenoble Alpes
  Tel: +33 4 76 76 37 65
  Mail: mmanceau@chu-grenoble.fr

1.2. Statistical Analysis Process

The statistical analysis will be performed by Marc Manceau, supervised by Matthieu Roustit. Before conducting the analyses planned in this statistical analysis plan (SAP), the SUNSAS database will be locked in accordance to SOP DM-04 Database lock management after the data has been cleaned and checked.

The final lock cannot occur until the following points have been completed:

- The CRFs have been signed by the principal investigator at each site, indicating that data reported is accurate and complete.
- All queries from the data managers (DM) and medical reviewers (MR) have been closed.
- The data review meeting (DRM) report has been signed by the relevant parties.
- The data management plan (DMP) has been signed by the relevant parties.
- This statistical analysis plan (SAP) has been signed by the relevant parties.

Once the database has been frozen, the data will be transferred to the statisticians following the transfer procedure outlined in the data transfer agreement.

Statistical analyses will then be performed following this SAP and reported in the statistical analysis report (SAR).

Docusign Envelope ID: B2C41F77-2931-48C9-868E-D7E9279F8B09

The R software (version ≥ 4.3) will be used to perform all the analyses, together with standard packages for data manipulation, visualization and analysis, including the "tidyverse" mega-package. All the code necessary to reproduce the analyses will be made available in the SAR.

2. Objectives of the Study

2.1. Study Design

This is a multicenter, prospective, randomized, controlled study with two open-label arms:

- The "PSG" arm (standard of care): the diagnostic method used is the in-laboratory or in-home polysomnography (PSG).
- The "Sunrise" arm (experimental): the diagnostic method is the Sunrise home sleep test device.

2.2. Primary Objectives

1. a) To demonstrate the non-inferiority of Sunrise over the reference method (PSG) on daytime sleepiness at 3 months after the diagnostic consultation.
   AND
   b) To demonstrate the superiority of Sunrise over the reference method (PSG) on the time between the inclusion (randomization) and the diagnostic consultation.
2. To demonstrate the superiority of Sunrise over the reference method (PSG) on the time between the inclusion (randomization) and the treatment dispensation, specifically for patients who are prescribed treatment (positive airway pressure (PAP) or oral appliance).
3. To demonstrate the superiority of Sunrise over the reference method (PSG) on daytime sleepiness at 3 months post-inclusion (randomization).

2.3. Primary Endpoints

1. a) Variation in Epworth sleepiness scale (ESS) score from baseline to 3 months post-diagnosis.
   AND
   b) Time (in days) between inclusion (randomization) and diagnostic consultation.
2. Time (in days) between inclusion (randomization) and treatment dispensation of PAP or oral appliance.
3. Variation in ESS score from baseline to 3 months post-inclusion (randomization).

2.4. Secondary Objectives

1. To demonstrate the superiority of Sunrise over the reference method (PSG) on the quality of life at 3 months post-inclusion (randomization).

2. To demonstrate the superiority of Sunrise over the reference method (PSG) on work productivity at 3 months post-inclusion (randomization).
3. To assess the economic impact of Sunrise, through a cost-effectiveness analysis expressed in cost per quality-adjusted life-year (QALY) gained at 3 months after the diagnostic consultation, compared to the reference method (PSG), from the payer's perspective.
4. To estimate the organizational and budgetary impact of deploying Sunrise in the French healthcare system over 3 years for the French health insurance (*only if the cost-effectiveness analysis is in favor of Sunrise*).
5. To demonstrate the non-inferiority of Sunrise over the reference method (PSG) on treatment adherence, specifically for patients who are treated by PAP, at 3 months after having started the treatment.
6. To confirm the diagnostic accuracy of Sunrise compared to the reference method (PSG) in the PSG arm.
7. To measure the within-subject inter-night variability of the OSA severity index with Sunrise.

2.5. Secondary Endpoints

1. Variation in short-form 36 (SF36) and Quebec sleep questionnaire (QSQ) scores from baseline to 3 months post-inclusion (randomization).
2. Variation in work productivity and activity impairment: sleep apnea syndrome (WPAI:SAS) score from baseline to 3 months post-inclusion (randomization).
3. Incremental cost-effectiveness ratio (ICER) of Sunrise compared to the reference method (PSG), expressed as the incremental cost (in €) per QALY gained 3 months post-diagnosis.
4. Net benefit (in €) for the public health insurance from the dissemination of Sunrise in the French healthcare system over 3 years.
5. Mean adherence to PAP over 30 days evaluated between D60 and D90 after PAP treatment initiation.
6. Diagnostic accuracy: sensitivity and specificity of Sunrise for detecting OSA at the recommended thresholds of 5 and 15 respiratory events per hour *(for patients included in the PSG arm only)*.
7. Inter-night variability of the number of respiratory events per hour measured with Sunrise on multiple nights at home (*for patients included in the Sunrise arm only*).

3. Number of Patients Required

The estimated sample size was calculated before the start of the study and is presented below as it appears in the protocol.

Because no previous data was available on the time between inclusion and diagnostic consultation (i.e., primary objective #1b), the sample size of the study was estimated based

Docusign Envelope ID: B2C41F77-2931-48C9-868E-D7E9279F8B09

on the first level of the hierarchy only (i.e., primary objective #1a) considering the following:

- Because the smallest clinically meaningful difference for ESS was estimated to be around 2.5 points (2 points in the smallest estimations)[1,2], we fixed a non-inferiority margin at 1 ESS point for the difference between 3 months post-diagnosis and baseline.
- We considered a standard error for the difference of 4.6[1,3].
- We fixed the significance level of the one-sided test at a value $\alpha = 0.025$.
- And we aimed at having a power equal to $1 - \beta = 0.85$.

Under this scenario, 381 patients per arm were necessary to show the non-inferiority of Sunrise over PSG, using a unilateral T test (PASS v15, NCSS LLC, Kaysville, Utah, USA).

Because we anticipated a dropout proportion of 10%, we aimed at including 848 patients in the study.

This sample allows to show an effect size, expressed as Cohen's D, of 0.23 and 0.26 with a false positive rate of 0.05 and 0.025, respectively, and a nominal power of 90%, using a bilateral T test on the other outcomes of the hierarchy. On the ESS, keeping the same standard error as considered above, this effect size would correspond to a difference of 1.2 points between the two arms.

References:

1. Crook S, Sievi NA, Bloch KE, Stradling JR, Frei A, Puhan MA, et al. Minimum important difference of the Epworth Sleepiness Scale in obstructive sleep apnoea: estimation from three randomised controlled trials. Thorax. 2019;74(4):390-6.
2. Patel S, Kon S, Nolan CM, Barker RE, Simonds AK, Morrell MJ, Man WDC. The Epworth Sleepiness Scale: Minimum Clinically Important Difference in Obstructive Sleep Apnea. Am J Respir Crit Care Med. 2018 Apr 1;197(7):961-963.
3. Ballester E, Badia JR, Hernández L, Carrasco E, de Pablo J, Fornas C, et al. Evidence of the effectiveness of continuous positive airway pressure in the treatment of sleep apnea/hypopnea syndrome. Am J Respir Crit Care Med. févr 1999;159(2):495-501.

4. Populations of Analysis and Handling of Missing Data

4.1. Populations of Analysis

The analysis of the objectives based on a superiority test should follow the *intent to treat* (ITT) principle, meaning that the analysis is performed on all randomized individuals, and that they are analyzed in the initially randomized group. However, since some data will probably be missing in the main analysis (where no missing data replacement is performed), the main analysis will be performed on a *modified intent to treat* (mITT) population. The mITT population, including all randomized patients with available data for the criterion being analyzed, will therefore be criterion-specific.

Docusign Envelope ID: B2C41F77-2931-48C9-868E-D7E9279F8B09

On the contrary, the analysis of the objectives based on a non-inferiority test will be based on the *per protocol* (PP) principle, which is more conservative in this case. The PP population includes all randomized patients with available data for the criterion being analyzed (no missing data replacement is performed), and without major deviations, as defined in the data review meeting report. Since major deviations may affect specifically one criterion but not another, the PP population will also be criterion-specific.

Additionally, the following sensitivity analyses will be performed for the primary objectives:

- On a PP population for the objectives based on a superiority test.
- On a mITT population for the objectives based on a non-inferiority test.
- On the ITT population for the objectives which are not analyzed by a survival analysis (i.e., time-related) after replacing missing data using multiple imputation methods, provided that the assumption of missing at random (MAR) is credible, as described in section 4.3.

4.2. Missing Data Report

We will first report the proportion of missing data for all endpoints based on the ITT population.

Time-related endpoints will be handled with specific consideration for missing data. If an event has not occurred due to patient's early termination (e.g., lost to follow-up, withdrawal of consent, maximum follow-up period of 18 months reached), the date will be right-censored at the patient's premature end-of-study date. If an event has not occurred by the end of the study (e.g., patient still awaiting for oral appliance treatment), the date will be right-censored at the date of database archiving.

We will report the proportion of dates for which censoring needs to be considered. This approach will apply to the following time-related endpoints:

- Time (days), between inclusion and diagnostic consultation.
- Time (days), between inclusion and treatment dispensation.

For patients in the PSG arm, we will report the proportion of patients where the PSG report scored by the central expert and/or the Sunrise report are missing.

Lastly, for patients in the Sunrise arm, we will report the proportion of patients who have 1, 2 or 3 nights of Sunrise recordings missing.

Table 1 below shows the missing data report in the two arms.

Docusign Envelope ID: B2C41F77-2931-48C9-868E-D7E9279F8B09

Table 1: Missing data report in the two study arms.

	PSG	Sunrise
ESS at baseline		
ESS at 3 months after diagnostic consultation		
ESS at 3 months after inclusion		
SF36 at baseline		
SF36 at 3 months after inclusion		
QSQ at baseline		
QSQ at 3 months after inclusion		
WPAI:SAS at baseline		
WPAI:SAS at 3 months after inclusion		
EQ-5D-5L at baseline		
EQ-5D-5L at 3 months after inclusion		
EQ-5D-5L at 3 months after diagnostic consultation		
PAP adherence data 3 months after first use of treatment		
Diagnostic consultation date		
Treatment dispensation date		
PSG report (central scoring)		NA
Sunrise report		NA
1 Sunrise report missing	NA	
2 Sunrise reports missing	NA	
3 Sunrise reports missing	NA	

4.3. Imputation of Missing Data

Missing data replacement will be considered only for the primary endpoints which are not analyzed by a survival analysis (i.e., time-related), and only as additional sensitivity analyses.

This will be performed following Rubin's multiple imputation strategy, using the "mice" R package.

More precisely, we propose to impute missing values of:

- ESS score at baseline.
- ESS score at 3 months after the diagnostic consultation.
- ESS score at 3 months after the inclusion.

This will be done by using a bootstrap imputation under a linear model fitted by taking into account other demographic covariates available for a sufficient number of patients (e.g., age, sex, cardiovascular history, sleep-related outcomes), as well as the arm status (Austin et al. 2021). In the "mice" R package, this method is called "norm.boot".

We will compute $m = 50$ datasets which will be used to compute the p-values of the two tests corresponding to primary objectives #1a and #3.

Reference :

Austin et al. 2021, Canadian Journal of Cardiology, Missing Data in Clinical Research: A Tutorial on Multiple Imputation.

5. Descriptive Analysis

The descriptive analysis will present the general characteristics of the ITT population, in both arms: PSG and Sunrise.

- Continuous variables will be summarized by their median and inter-quartile range.
- Qualitative variables will be summarized as raw numbers and proportions of each factor value.

Variables of the descriptive analysis are presented in Table 2 below (non-exhaustive).

Table 2: Descriptive analysis of baseline demographic variables in the two study arms.

	PSG	Sunrise
Sex		
Age		
Weight		
Height		
BMI		
Type 2 diabetes		
Hypertension		

Docusign Envelope ID: B2C41F77-2931-48C9-868E-D7E9279F8B09

LVEF		
NYHA functional class		
II		
III		
Medications (ATC)		
Insulins and analogues (A10A)		
Blood glucose lowering drugs, excluding insulins (A10B)		
Diuretics (C03)		
Beta blocking agents (C07)		
Calcium channel blockers (C08)		
Agents acting on the renin–angiotensin system (C09)		
Lipid modifying agents (C10)		
Baseline ESS score		
Baseline QSQ score		
Baseline SF36 score		
Baseline WPAI:SAS score		
Baseline EQ-5D-5L score		
AHI based on PSG (central scoring)		NA
AHI based on Sunrise – first night	NA	
AHI based on Sunrise – second night	NA	
AHI based on Sunrise – third night	NA	

6. Primary Objectives

6.1. Sequential Analysis Hierarchy

Four distinct primary objectives will be sequentially tested over three levels, following a hierarchy which had been pre-specified in the protocol.

To maintain control over the overall Type I error rate for all primary objectives, we will employ a hierarchical testing approach. Each subsequent level in the hierarchy will only be tested if the null hypothesis at the preceding level is rejected. At each level of the hierarchy, a one-sided significance level of 0.025 will be applied. Importantly, at the first level, both null hypotheses must be rejected before proceeding to test the second level.

Docusign Envelope ID: B2C41F77-2931-48C9-868E-D7E9279F8B09

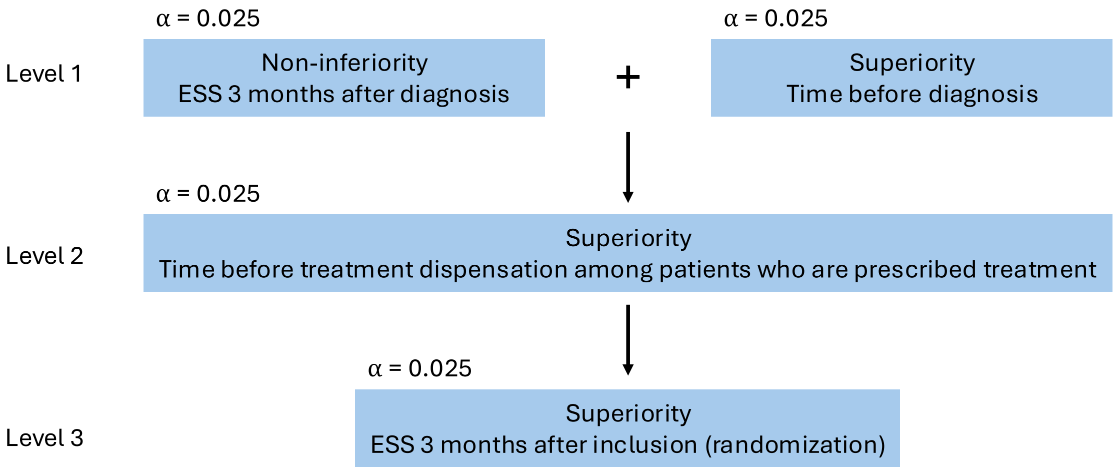

6.2. Non-inferiority on the ESS at 3 Months After Diagnostic Consultation

Outcome: Variation between the ESS score measured at 3 months after the diagnostic consultation and baseline:

$$\Delta = X_{diag\,+\,3M} - X_0$$

Arms: PSG (P) and Sunrise (S).

Estimand: Difference between the expectation of Δ among patients in P (Δ_P) and the expectation of Δ among patients in S (Δ_S):

$$E = \Delta_P - \Delta_S$$

Population: This non-inferiority analysis will be based on the PP population including all randomized patients with available data to analyze this endpoint, i.e., an ESS score available at both baseline and 3 months after the diagnostic consultation, and without major deviations affecting the principal objective #1a.

Statistical analysis: We will estimate our estimand based on the available data using the difference between the empirical means of Δ among patients in P and patients in S, for which data are available.

$$\overline{E} = \overline{\Delta_P} - \overline{\Delta_S} = \frac{1}{n_P} \sum_{i \in P} \Delta_i - \frac{1}{n_S} \sum_{i \in S} \Delta_i$$

A unilateral T test will then be performed, with a non-inferiority margin test of $\delta = 1$ ESS point, meaning that the following two hypotheses will be tested:

- H0: $E \geq \delta$
- H1: $E < \delta$

Significance level: This unilateral test will be performed with a significance level of 0.025.

Docusign Envelope ID: B2C41F77-2931-48C9-868E-D7E9279F8B09

Associated figures: A graphical comparison of the ESS distribution among patients in P and patients in S will be proposed, with (i) a figure superimposing the histograms of Δ values in both groups, (ii) a boxplot showing side by side the distribution of Δ values in both groups, (iii) a boxplot showing side by side the distribution of X_0 and $X_{diag\,+\,3M}$ in both groups.

6.3. Superiority on the Time Between Inclusion and Diagnostic Consultation

Outcome: Time between inclusion t_0 and diagnostic consultation t_{diag}:

$$D = t_{diag} - t_0$$

Arms: PSG (P) and Sunrise (S).

Estimand: We are interested in hazard functions $h(d)$, which denote the rate at which diagnosis occurs, conditional on having $D > d$. In this analysis, we assume that the hazard in the P arm, $h_P(d)$, and the hazard in the S arm, $h_S(d)$, are proportional to each other, meaning that there exists a constant r, called the hazard ratio, which satisfies:

$$\forall d, r = \frac{h_P(d)}{h_S(d)}$$

Population: This superiority analysis will be based on the mITT population. The date of inclusion is assumed to always be available. In the case of a missing date for diagnosis due to patient's early termination (e.g., lost to follow-up, withdrawal of consent, maximum follow-up period of 18 months reached), the date will be right-censored using the patient's premature end-of-study date t_1, meaning that the statistical analysis will consider that $D > t_1 - t_0$.

We will include estimates of the proportion of censoring in both study arms to evaluate whether the censoring is likely to be non-informative.

Statistical analysis: We will use the Cox proportional hazards model to compare the hazard in the P arm, $h_P(t)$, to the hazard in the S arm, $h_S(t)$. More precisely, the following two hypotheses will be tested:

- H0: $r \geq 1$
- H1: $r < 1$

Significance level: We will consider a unilateral 0.025 significance level.

Associated figure: A Kaplan-Meier graph showing the estimated survival curves in both arms will accompany this analysis, where *survival* refers to the time before getting a diagnosis.

6.4. Superiority on the Time Between Inclusion and Treatment Dispensation

Outcome: Time between inclusion t_0 and treatment dispensation t_{treat}:

Docusign Envelope ID: B2C41F77-2931-48C9-868E-D7E9279F8B09

$$D = t_{treat} - t_0$$

Arms: PSG (P) and Sunrise (S).

Estimand: We are interested in hazard functions $h(d)$, which denote the rate at which treatment dispensation occurs, conditional on having $D > d$. In this analysis, we assume that the hazard in the P arm, $h_P(d)$, and the hazard in the S arm, $h_S(d)$, are proportional to each other, meaning that there exists a constant r, called the hazard ratio, which satisfies:

$$\forall d, r = \frac{h_P(d)}{h_S(d)}$$

Population: This superiority analysis will be based on the mITT population and will specifically include patients who are prescribed treatment (PAP or oral appliance). If the treatment is either refused by the patient or contraindicated by a specialist, the patient will not be included in the analysis. If the information about whether the patient requires treatment is missing, we will assume that the treatment is not needed. The date of inclusion is assumed to always be available. In the case of a missing date of treatment dispensation due to patient's early termination (e.g., lost to follow-up, withdrawal of consent, maximum follow-up period of 18 months reached), the date will be right-censored using the patient's premature end-of-study date t_1, meaning that the statistical analysis will consider that $D > t_1 - t_0$. If treatment dispensation has not occurred by the end of the study (e.g., patient still awaiting for oral appliance treatment), the date will be right-censored at the date of database archiving.

We will include estimates of the proportion of censoring in both study arms to evaluate whether the censoring is likely to be non-informative.

Statistical analysis: We will use the Cox proportional hazards model to compare the hazard in the P arm, $h_P(t)$, to the hazard in the S arm, $h_S(t)$. More precisely, the following two hypotheses will be tested:

- H0: $r \geq 1$
- H1: $r < 1$

Significance level: We will consider a unilateral 0.025 significance level.

Associated figure: A Kaplan-Meier graph showing the estimated survival curves in both arms will accompany this analysis, where *survival* refers to the time before treatment dispensation.

6.5. Superiority on the ESS at 3 Months After Inclusion

Outcome: Variation between the ESS score measured at 3 months after the inclusion and baseline:

$$\Delta = X_{Incl + 3M} - X_0$$

Arms: PSG (P) and Sunrise (S).

Estimand: In this analysis, it is assumed that Δ depends both on the arm and on the baseline value X_0. More precisely, we model Δ as:

$$\Delta = a + bX_0 + c\delta_S + \epsilon$$

Where a is the intercept, b is the coefficient representing the expected increase in a unit ESS at 3 months for each unit ESS at baseline, c is the additional effect on ESS at 3 months for patients in the S group, and $\epsilon \sim N(0, \sigma^2)$ is the inter-individual variation.

Based on this model formulation, our estimand of interest is c.

Population: This superiority analysis will be based on the mITT population including all randomized patients with available data to analyze this endpoint, i.e., an ESS score available at both baseline and 3 months after the inclusion.

Statistical analysis: We will fit the above-mentioned model to the data, i.e., perform an ANCOVA (Analysis of Covariance) aimed at estimating c and testing:

- H0: $c \geq 0$
- H1: $c < 0$

A transformation of the data might be considered if the residuals cannot be assumed to follow a Gaussian distribution.

Significance level: This unilateral test will be performed with a significance level of 0.025.

Associated figures: A graphical comparison of the ESS distribution among patients in P and patients in S will be proposed, with (i) a figure superimposing the histograms of Δ values in both groups, (ii) a boxplot showing side by side the distribution of Δ values in both groups, (iii) a boxplot showing side by side the distribution of X_0 and X_{3M} in both groups.

7. Secondary Objectives

7.1. Superiority on SF36 and QSQ at 3 Months After Inclusion

Outcome: Variation between the questionnaire scores measured at 3 months after the inclusion and baseline:

$$\Delta = X_{Incl+3M} - X_0$$

Arms: PSG (P) and Sunrise (S).

Estimand: In this analysis, it is assumed that Δ depends both on the arm and on the baseline value X_0. More precisely, we model Δ as:

$$\Delta = a + bX_0 + c\delta_S + \epsilon$$

Docusign Envelope ID: B2C41F77-2931-48C9-868E-D7E9279F8B09

Where a is the intercept, b is the coefficient representing the expected increase in a unit score at 3 months for each unit score at baseline, c is the additional effect on the score at 3 months for patients in the S group, and $\epsilon \sim N(0, \sigma^2)$ is the inter-individual variation.

Based on this model formulation, our estimand of interest is c.

Population: This superiority analysis will be based on the mITT population including all randomized patients with available data to analyze this endpoint, i.e., questionnaire scores available at both baseline and 3 months after the inclusion.

Statistical analysis: We will fit the above-mentioned model to the data, i.e., perform an ANCOVA (Analysis of Covariance) aiming at estimating c and testing:

- H0: $c \leq 0$
- H1: $c > 0$

A transformation of the data might be considered if the residuals cannot be assumed to follow a Gaussian distribution.

Precise outcomes: Note that the SF36 questionnaire has one score per subscale (8 in total):

- Physical functioning (10 items).
- Role limitations due to physical health (4 items).
- Role limitations due to emotional problems (4 items).
- Energy/fatigue (4 items).
- Emotional well-being (5 items).
- Social functioning (2 items).
- Pain (2 items).
- General health (5 items).

We will thus perform separate tests for each of the eight SF36 domains individually, as well as for the overall Physical Component Summary (PCS) and Mental Component Summary (MCS) scores, to capture both detailed domain-specific effects and broader physical and mental health outcomes.

The QSQ questionnaire consists of 32 items grouped into five domains:

- Daytime sleepiness
- Diurnal symptoms
- Nocturnal symptoms
- Emotions
- Social interactions

Mean scores for each domain and a total score will be calculated. If an item is not answered, it is common to adjust the domain average based on the number of items answered. However, if more than 20% of the items in a domain are missing, that domain score is considered unreliable.

Significance level: This unilateral test will be performed with a significance level of 0.025.

Associated figures: A graphical comparison of the questionnaire scores distributions among patients in P and patients in S will be proposed, with (i) a figure superimposing the histograms of Δ values in both groups, (ii) a boxplot showing side by side the distribution of Δ values in both groups, (iii) a boxplot showing side by side the distribution of score values in both groups and at both times.

7.2. Superiority on WPAI:SAS at 3 Months After Inclusion

The same analysis performed for the previous secondary endpoints will be applied to this endpoint as well.

Based on the WPAI:SAS questionnaire, four scores will be computed:

- Work time missed (absenteeism).
- Impairment while working (presenteeism).
- Overall work impairment (work productivity loss).
- Impairment in regular activities (activity impairment).

Each of these scores is expressed as a percentage, with higher percentages indicating greater impairment and loss of productivity. A separate test will be performed for each of these scores.

7.3. Cost-effectiveness Analysis

Population: This superiority analysis will be based on the mITT population including all randomized patients with available data, i.e., patient diary data and EQ-5D-5L scores available at baseline, 3 months after the inclusion and 3 months after the diagnostic consultation.

Outcomes: Four outcomes of interest measured for each patient are key for the cost-effectiveness analysis:

1. The total cost of care during the study participation, C, expressed in €.
2. The utility U_0 measured at baseline.
3. The utility U_{3M} measured at 3 months after the inclusion.
4. The utility $U_{diag+3M}$ measured at 3 months after the diagnosis.

Utilities are measured through the EQ-5D-5L form, with utility scores validated in the French population.

Utilities will be used to compute the so-called quality-adjusted life-year (QALY), denoted as Q, for a given individual as:

$$Q \times (t_0 - t_{diag+3M}) = \frac{U_0 + U_{3M}}{2} \times (t_{3M} - t_0) + \frac{U_{3M} + U_{diag+3M}}{2} \times (t_{diag+3M} - t_{3M})$$

Arms: PSG (P) and Sunrise (S).

Estimands: The following estimands are of interest:

1. The difference between the expected cost in S, C_S, and the expected cost in P, C_P:

$$\backslash DeltaC = C_S - C_P$$

2. The incremental cost-effectiveness ratio (ICER), which is the ratio between $\backslash DeltaC$ and $\backslash DeltaQ$, where $\backslash DeltaQ$ refers to the difference between the expected utility in S and the expected utility in P:

$$ICER = \frac{\backslash DeltaC}{\backslash DeltaQ} = \frac{C_S - C_P}{Q_S - Q_P}$$

3. For any λ value representing the payer's willingness to pay for an additional QALY, the incremental net benefit (INB) is defined as:

$$b(\lambda) = \lambda \times \backslash DeltaQ - \backslash DeltaC$$

Statistical analysis: First, $\backslash DeltaC$ will be estimated based on the difference between the empirical means of costs in both arms.

Second, a raw estimation of the ICER will be computed, along with a 95% bootstrap confidence interval.

Third, $b(\lambda) = \lambda \times \backslash DeltaQ - \backslash DeltaC$ will be estimated, where λ is the maximal value the payer is willing to pay for an additional QALY, adjusted for stratification factors of the randomization as well as for other potential confusion factors, using linear regression analysis.

The cost-effectiveness probability (PE) of Sunrise's device as compared to PSG will be computed based on the INB distribution for a given value of λ. We will plot the acceptability curve of the differential cost-effectiveness ratio, showing PE as a function of λ, for values of λ between 0 and 100,000€.

Significance level: 95% confidence intervals will be computed around point estimates.

Additional figures: A figure showing the histograms of costs in both groups will be produced, along with a boxplot summarizing the same distributions.

Once $\backslash DeltaC$ and $\backslash DeltaQ$ have been estimated, they will be used to estimate and plot the curve showing the estimated INB $\hat{b}(\lambda)$ as a function of λ, for values of λ between 0 and 100,000€.

7.4. Net Benefit for the Health Insurance of Sunrise's Diffusion in France

Estimand: The net benefit for Health Insurance from the diffusion of the Sunrise diagnostic device within the French health system after three years.

Statistical analysis: This objective will be based on a deterministic estimation of costs, without any additional statistical analyses beyond the previous estimation of \$DeltaC$.

7.5. Non-inferiority on the Adherence to PAP at 3 Months After the First Use of Treatment

Outcomes: PAP adherence will be measured for each patient based on the mean duration of device use per night, A_i, over the 30-day time window starting at day 60 after the first treatment use, expressed in minutes per night.

Arms: PSG (P) and Sunrise (S).

Estimand: The difference between the expected PAP use duration in the P arm, A_P, and the expected PAP use duration in the S arm, A_S:

$$E = A_P - A_S$$

Population: This non-inferiority analysis will be based on the PP population including all randomized patients who were prescribed PAP, with available PAP adherence data in the targeted window and without major deviations affecting the secondary objective #5.

Statistical analysis: We will estimate the estimand using the difference between the empirical means of A among patients in the P and S arms, for whom data are available:

$$\overline{E} = \overline{A_P} - \overline{A_S} = \frac{1}{n_P} \sum_{i \in P} A_i - \frac{1}{n_S} \sum_{i \in S} A_i$$

A unilateral T test will then be performed with a non-inferiority margin $\delta = 30$ minutes (Malhotra et al., 2023). Therefore, the following two hypotheses will be tested:

- H0: $E \geq \delta$
- H1: $E < \delta$

Significance level: This unilateral test will be performed with a significance level of 0.025.

Associated figures: A figure visually comparing the histograms of A in both groups will be produced, along with a boxplot showing side by side distributions in both groups.

Reference:

Malhotra A, Sterling KL, Cistulli PA, Pépin JL, Chen J, Woodford C, Alpert N, More S, Nunez CM, Benjafield AV. Dose-Response Relationship between Obstructive Sleep Apnea Therapy Adherence and Healthcare Utilization. Ann Am Thorac Soc. 2023 Jun;20(6):891-897.

7.6. Diagnostic Accuracy of Sunrise

Outcomes: Two measures of interest are available for each patient, concerning the same night:

1. X_i, the number of respiratory events per hour measured by PSG (central scoring).
2. Y_i, the number of respiratory events per hour measured by Sunrise.

Arm: PSG only.

Estimands: The sensitivity (Se) and specificity (Sp) of Y as compared to the gold standard X to detect:

- Patients with more than $r_1 = 5$ respiratory events per hour.
- Patients with more than $r_2 = 15$ respiratory events per hour.

$$Se(r) = P(Y_i > r \mid X_i > r)$$

$$Sp(r) = P(Y_i < r \mid X_i < r)$$

Population: This analysis will include all patients randomized in the PSG arm for whom both measures (X_i and Y_i) are available.

Statistical analysis: We will estimate $Se(r_1), Sp(r_1), Se(r_2), Sp(r_2)$ based on the empirical proportions of these events in our sample. For each of these four quantities, a 95% confidence interval will also be estimated.

Associated tables and figures: For each of these diagnostic tasks, a 2 x 2 contingency table of raw counts showing the diagnosis of interest against the gold standard centralized PSG diagnosis will be produced.

Receiver operating characteristic (ROC) curves will also be computed and displayed, representing $Se(r)$ as a function of $\left(1 - Sp(r)\right)$ for all possible values of r.

We will additionally follow Bland & Altman's method for quantifying the agreement between X and Y, consisting of:

- Computing $\Delta_i = Y_i - X_i$ and $M_i = \frac{Y_i + X_i}{2}$.
- Plotting the histogram of (Δ_i) values.
- Plotting the scatterplot of Δ_i against M_i.
- Estimating the prediction interval of Δ values.

7.7. Inter-night Variability of the Number of Respiratory Events per Hour

Outcome: The number of respiratory events per hour measured by Sunrise.

Arm: Sunrise only.

Estimands: We are interested in two estimands used to assess the variability of a series of measures. Both quantities are based on a mixed-effects linear model, with the following equation modeling measure j on individual i:

$$Y_{ij} = \mu + \alpha_i + \epsilon_{ij}$$

Docusign Envelope ID: B2C41F77-2931-48C9-868E-D7E9279F8B09

where $\alpha_i \sim N(0, \sigma_\alpha^2)$ are random effects shared for all measures of patient i, and $\epsilon_{ij} \sim N(0, \sigma_\epsilon^2)$ are random noise errors.

Our two target estimands are:

- The within-subject coefficient of variation: $wsCV = \frac{\sigma_\alpha}{\mu}$.
- The intra-class correlation coefficient: $ICC = \frac{\sigma_\alpha^2}{\sigma_\alpha^2 + \sigma_\epsilon^2}$.

Population: This analysis will include all patients randomized in the Sunrise arm with at least two nights of Sunrise recording available.

Statistical analysis: We will fit the mixed-effects linear model described above using R and estimate wsCV and the ICC using the R package "agRee".

Associated figure: Values of wsCV and ICC will be provided. Additionally, a plot showing the index values on the y-axis for all individuals on the x-axis will be produced for a visual assessment of the variability of the measure.

Docusign Envelope ID: B2C41F77-2931-48C9-868E-D7E9279F8B09

Table 3: Summary of estimands and analyses associated with the objectives of the study.

Objective	Outcome	Estimand	Population (main analysis)	Analysis
**Primary objectives**				
Non-inferiority on sleepiness	Variation of ESS at baseline and at M3 after diagnosis	Difference of ESS variation between arms	PP	T test, non-inferiority margin = 1, unilateral alpha = 0.025
Superiority on time before diagnosis	Time (d) before diagnosis	Hazard ratio between arms	mITT	Cox model + Kaplan-Meier
Superiority on time before treatment dispensation	Time (d) before treatment dispensation	Hazard ratio between arms	mITT, patients who are prescribed treatment	Cox model + Kaplan-Meier
Superiority on sleepiness	Variation of ESS at baseline and at M3 after inclusion	The effect of the arm in a linear model	mITT	ANCOVA, adjusting on baseline ESS
**Secondary objectives**				
Superiority on quality of life	Variation of QSQ and SF36 scores at baseline and at M3 after inclusion	The effect of the arm in a linear model	mITT	ANCOVA, adjusting on baseline score
Superiority on work productivity	Variation of WPAI:SAS score at baseline and at M3 after inclusion	The effect of the arm in a linear model	mITT	ANCOVA, adjusting on baseline score
Assess the ICER of Sunrise over PSG (superiority)	Incremental cost (€) per QALY gained at M3 after diagnosis	Cost difference, QALY difference, ICER, INB	mITT	Parameter estimation
Assess the budget impact of Sunrise diffusion	Net benefit for Health Insurance	NA	NA	Simulations
Non-inferiority on PAP adherence	PAP usage per night over the 30-day time window starting at day 60 after the first treatment use	Difference of mean use duration	PP, patients having initiated a PAP treatment	T test, non-inferiority margin = 30 min, unilateral alpha = 0.025
Evaluate Sunrise diagnostic accuracy	# respiratory events per sleep hour	Se, Sp	Patients in the PSG arm only	Proportion estimation (Se, Sp) + ROC curve + Bland Altman plot
Evaluate Sunrise within-subject variability	# respiratory events per sleep hour	wsCV, ICC	Patients in the Sunrise arm only	Mixed-effects linear model
