# Supplementary material for: Time to diagnosis and treatment of obstructive sleep apnoea using mandibular jaw movement monitoring versus polysomnography: an open-label, multicentre, randomised, controlled trial
Source: Lancet Reg Health Eur. 2026 Mar 17;64:101637. doi: 10.1016/j.lanepe.2026.101637 (PMC13147807; doi:10.1016/j.lanepe.2026.101637)
Supplement: SUNSAS study investigators [file mmc4.docx]

**SUNSAS study investigators**

| **First & middle names** | **Surname** |
| --- | --- |
| Sarah | Alexandre |
| Sébastien | Baillieul |
| Lucie | Barateau |
| Clara | Bianquis |
| Yvan | Bellanger |
| Hélène | Benzaquen |
| Laurent | Boyer |
| Nicolas | Carpentier |
| Elena | Charbonnier |
| Ari | Chaouat |
| Rita | Clin |
| Julien | Coelho |
| Ala | Covali |
| Yves | Dauvilliers |
| Claire | Denis |
| Marie | Destors |
| Marie Pia | d’Ortho |
| Antoine | Dumazet |
| Justine | Frija |
| Sacha | Gaillard |
| Thibaut | Gentina |
| Maëlle | Guellerin |
| Kelly | Guichard |
| Laurence | Hertert-Gandolfo |
| Benjamin | Huret |
| Antoine | Jaffiol |
| François | Jounieaux |
| Philippe | Lang |
| Claire | Launois |
| Damien | Léger |
| Smaranda | Leu-Semenescu |
| Rim | Leymarie |
| Cécile | Londner |
| Quentin | Lorber |
| Marie-Noëlle | Lothe-Cartier |
| Régis | Luraine |
| Guillaume | Marchand |
| Nicole | Meslier |
| Jean-Arthur | Micoulaud-Franchi |
| Pierre-Jean | Monteyrol |
| Pauline | Mulette |
| Marina | Ogier |
| Cécile | Olivier |
| Caroline | Pagniez |
| Albert | Pajon |
| Yasmina | Pascaud-Mansour |
| Laure | Peter-Derex |
| Pierre | Philip |
| Carole | Planès |
| Sandrine | Pontier-Marchandise |
| Vincent | Puel |
| Nathalie | Raymond |
| Bruno | Ribeiro-Baptista |
| Rodrigue | Ribereau |
| François | Ricordeau |
| Cécile | Ropars |
| Marc | Sapène |
| Kamila | Sedkaoui |
| Emeric | Stauffer |
| Jonathan | Taieb |
| Marion | Tailland |
| Annie | Tangtakoun |
| Pierre | Tankere |
| Wojciech | Trzepizur |
| Camille | Valery |
| Olivier | Varnet |
| Anne | Wittenberg |
