# Supplementary material for: Time to diagnosis and treatment of obstructive sleep apnoea using mandibular jaw movement monitoring versus polysomnography: an open-label, multicentre, randomised, controlled trial
Source: Lancet Reg Health Eur. 2026 Mar 17;64:101637. doi: 10.1016/j.lanepe.2026.101637 (PMC13147807; doi:10.1016/j.lanepe.2026.101637)
Supplement: French translation of the abstract [file mmc5.docx]

**French translation of the abstract**

**This translation in French was submitted by the authors and we reproduce it as supplied. It has not been peer reviewed. The editorial processes of the Lancet Regional Health - Europe have only been applied to the original abstract in English, which should serve as reference for this manuscript.**

**Cette traduction en français a été fournie par les auteurs et est reproduite telle que soumise. Elle n’a pas fait l’objet d’une évaluation par les pairs. Les processus éditoriaux du Lancet Regional Health - Europe ont uniquement été appliqués au résumé original en anglais, qui doit servir de référence pour ce manuscrit.**

**Délais de diagnostic et de mise sous traitement dans le syndrome d’apnées obstructives du sommeil avec le monitoring des mouvements mandibulaires comparé à la polysomnographie : essai randomisé contrôlé multicentrique en ouvert**

**Contexte** Le syndrome d’apnées obstructives du sommeil (SAOS) reste fréquemment sous-diagnostiqué, soulignant la nécessité d’alternatives diagnostiques pouvant être déployées à grande échelle. Cette étude visait à comparer un nouveau dispositif de diagnostic du SAOS à domicile (analyse des mouvements mandibulaires [MJM] assistée par intelligence artificielle [IA]) à la polysomnographie (PSG), en termes de délais pour accéder au diagnostic et au traitement, et de critères de jugement rapportés par les patients (patient-reported outcomes, PROs).

**Méthodes** Cette étude prospective, multicentrique, randomisée, contrôlée, en ouvert, a été menée en France (octobre 2021–octobre 2024). Des adultes âgés de 18 à 80 ans, adressés pour suspicion de SAOS, ont été randomisés (1:1) pour bénéficier d’un test diagnostic, soit via un enregistrement des MJM (dispositif Sunrise), soit via une PSG. Les critères de jugement principaux ont suivi une analyse hiérarchisée : (1) somnolence diurne (score de l’échelle de somnolence d’Epworth [ESS]) à 3 mois post-diagnostic et délai jusqu’au diagnostic ; (2) délai jusqu’à l’initiation du traitement ; et (3) somnolence diurne à 3 mois post-randomisation. Les critères secondaires incluaient la qualité de vie (SF-36, questionnaire du sommeil du Québec [QSQ]), la productivité au travail (questionnaire Work Productivity and Activity Impairment [WPAI]) et l’adhérence au traitement par pression positive continue (PPC) à 3 mois après le début du traitement.

**Résultats** Parmi 849 participants randomisés (58·7 % d’hommes, âge médian 50 ans, indice de masse corporelle 28·0 kg/m², indice d’apnées-hypopnées 15·2/h), 774 ont reçu un diagnostic : 133 avec une absence de SAOS, 239 avec un SAOS léger, 220 avec un SAOS modéré et 182 avec un SAOS sévère. Le délai médian jusqu’au diagnostic (15 vs 106 jours) et jusqu’à l’initiation du traitement (50 vs 124 jours) était significativement plus court dans le bras MJM par rapport au bras PSG (p<0·01 pour les deux groupes). Le diagnostic basé sur les MJM était non-inférieur à la PSG pour la réduction du score ESS à 3 mois après le diagnostic (–2·26 vs –2·29 ; différence [IC 95 %] : –0·85 à 0·79 ; p=0·01), et supérieur à 3 mois post-randomisation (différence : –1·51 [IC 95 % : –2·17 à –0·85] ; p<0·01). Les critères secondaires favorisaient également l’approche MJM.

**Interprétation** Une stratégie diagnostique du SAOS reposant sur l’analyse des MJM assistée par IA est non-inférieure à la PSG pour l’amélioration de la somnolence diurne à 3 mois post-diagnostic, tout en permettant un diagnostic et une mise sous traitement significativement plus précoces, résultant en une amélioration plus rapide de la somnolence diurne.

**Financement** Sunrise, avec le soutien du ministère français de la Santé dans le cadre du programme *Forfait Innovation*.
